# Supplementary material for: Enhanced P-TEFb activity compromises dentate gyrus neurogenesis in mice
Source: EMBO J. 2026 Mar 23;45(9):3102–23. doi: 10.1038/s44318-026-00752-w (PMC13144724; doi:10.1038/s44318-026-00752-w)
Supplement: Supplementary file 2 — Appendix [file 44318_2026_752_MOESM2_ESM.pdf]

Appendix for:

Enhanced P-TEFb activity compromises dentate gyrus neurogenesis in mice

Yin Fang[1, 3], Tong Qiu[1, 3], Ping Wang[2, 3], Shujun Bai[1], Min Wang[1], Chao Yang[1], Yan Wang[1], Peixuan Zhang[1], He Wang[2], Shanling Liu[2, *], Xue Xiao[1, *], Qintong Li[1, 4, *]

Table of contents:

Appendix Figure S1 – Page 2
Appendix Figure S2 – Page 3-4
Appendix Figure S3 – Page 5
Appendix Figure S4 – Page 6
Appendix Figure S5 – Page 7
Appendix Figure S6 – Page 8
Appendix Figure S7 – Page 9
Appendix Figure S8 – Page 10
Appendix Figure S9 – Page 11
Reference – Page 12

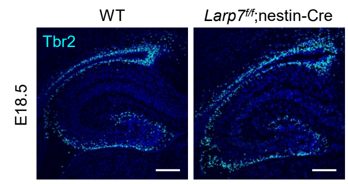

Appendix Figure S1. Tbr2⁺ intermediate progenitor cells along the dentate migratory stream at E18.5.

At E18.5, the number of Tbr2$^+$ intermediate progenitor cells along the dentate migratory stream were indistinguishable between wild-type and *Larp7*$^{f/f}$;nestin-Cre brains.

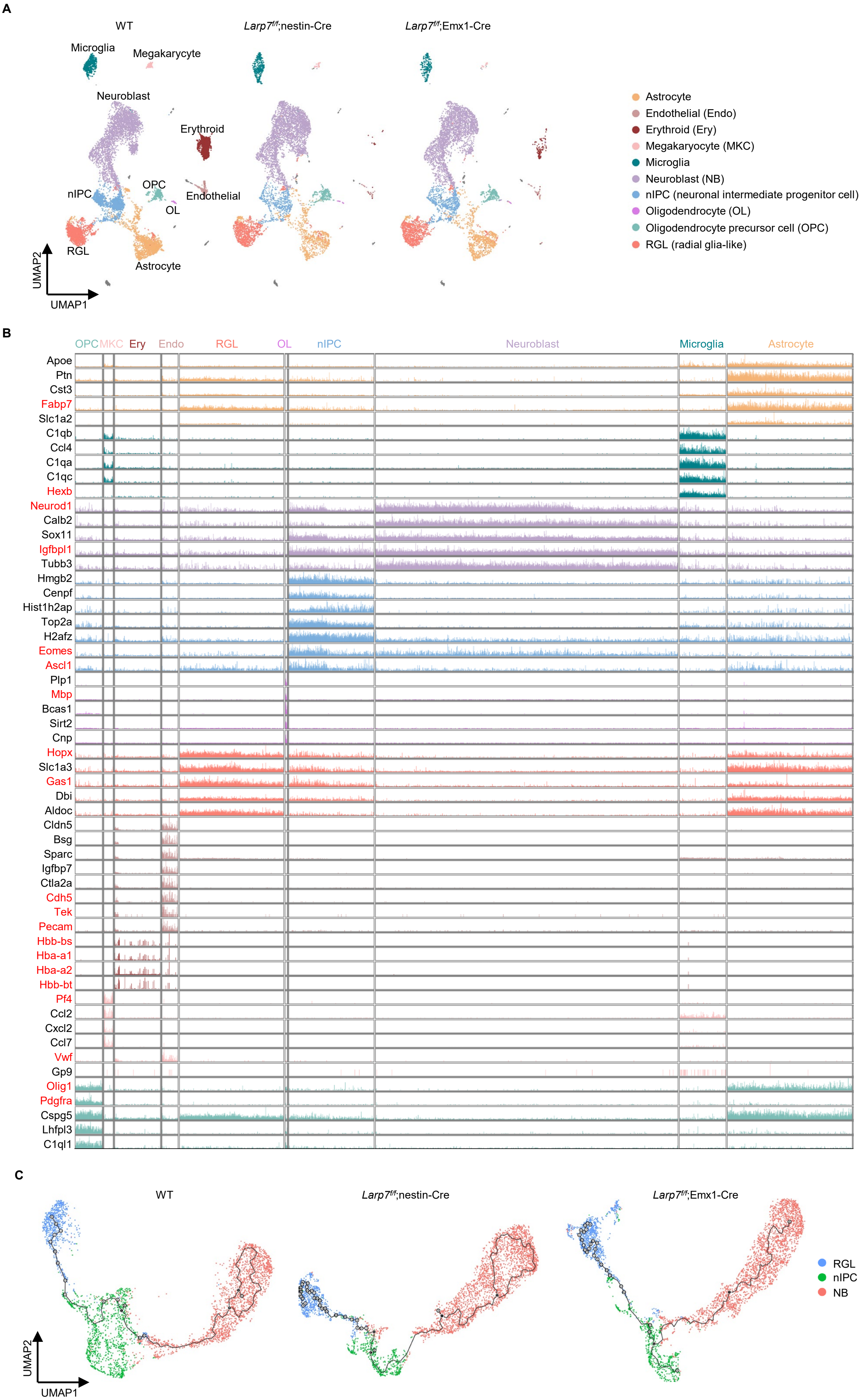
A
WT
Larp7f/f;nestin-Cre
Larp7f/f;Emx1-Cre
Microglia
Megakaryocyte
Neuroblast
Erythroid
OPC
nIPC
Endothelial
OL
RGL
Astrocyte
UMAP2
UMAP1
Astrocyte
Endothelial (Endo)
Erythroid (Ery)
Megakaryocyte (MKC)
Microglia
Neuroblast (NB)
nIPC (neuronal intermediate progenitor cell)
Oligodendrocyte (OL)
Oligodendrocyte precursor cell (OPC)
RGL (radial glia-like)
B
OPC MKC Ery Endo RGL OL nIPC Neuroblast Microglia Astrocyte
Apoe
Ptn
Cst3
Fabp7
Slc1a2
C1qb
Ccl4
C1qa
C1qc
Hexb
Neurod1
Calb2
Sox11
Igfbpl1
Tubb3
Hmgb2
Cenpf
Hist1h2ap
Top2a
H2afz
Eomes
Ascl1
Plp1
Mbp
Bcas1
Sirt2
Cnp
Hopx
Slc1a3
Gas1
Dbi
Aldoc
Cldn5
Bsg
Sparc
Igfbp7
Ctla2a
Cdh5
Tek
Pecam
Hbb-bs
Hba-a1
Hba-a2
Hbb-bt
Pf4
Ccl2
Cxcl2
Ccl7
Vwf
Gp9
Olig1
Pdgfra
Cspg5
Lhfpl3
C1ql1
C
WT
Larp7f/f;nestin-Cre
Larp7f/f;Emx1-Cre
RGL
nIPC
NB
UMAP2
UMAP1

Appendix Figure S2. Single-cell RNA sequencing analysis of P7 dentate gyri.

(A) UMAP presentation of all cell types, identified by single-cell sequencing, from microdissected dentate gyrus from P7 wild-type (WT), Larp7$^{f/f}$;nestin-Cre, and Larp7$^{f/f}$;Emx1-Cre mutant mice.

(B) Single-cell expression of enriched marker genes for each cell group identified by unbiased clustering (Seurat). Cluster-specific, differentially expressed genes (top 5 per cluster) were shown. Genes in red are established markers by previous studies. Annotated cell types are grouped by columns, and genes are organized by their associated cell types. Scale bars denote the expression level of indicated genes per cell.

(C) The differentiation trajectory inferred by Monocle 3.

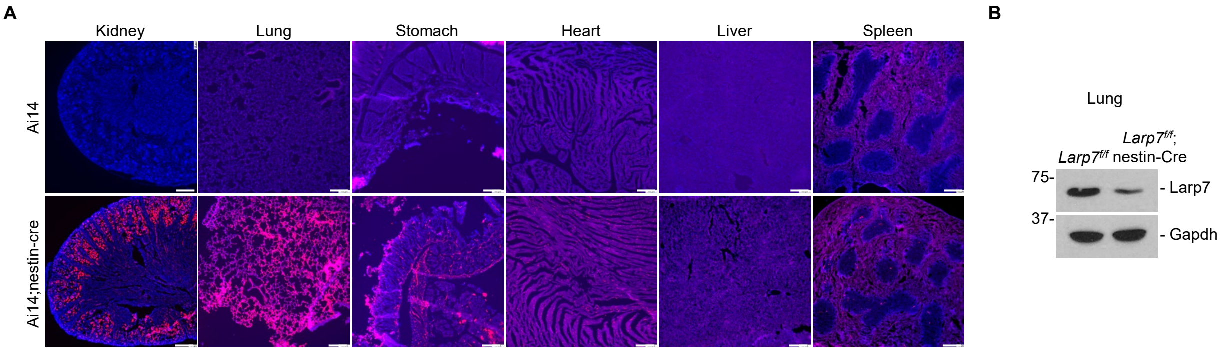

Appendix Figure S3. The analysis of the tissue distribution of nestin-Cre recombination activity.

(A) Representative images showing tdTomato expression in tissue sections from the kidney, lung, stomach, heart, liver, and spleen of Ai14 and Ai14;nestin-Cre mice. Ai14 is a Cre reporter tool strain that will express robust tdTomato fluorescence after Cre-mediated recombination[12]. In this case, tdTomato will exhibit red fluorescence in any cells in which nestin-Cre is expressed. Scale bar: 200 µm.

(B) Protein blot analysis of indicated proteins in the lung from Ai14 and Ai14;nestin-Cre mice, respectively.

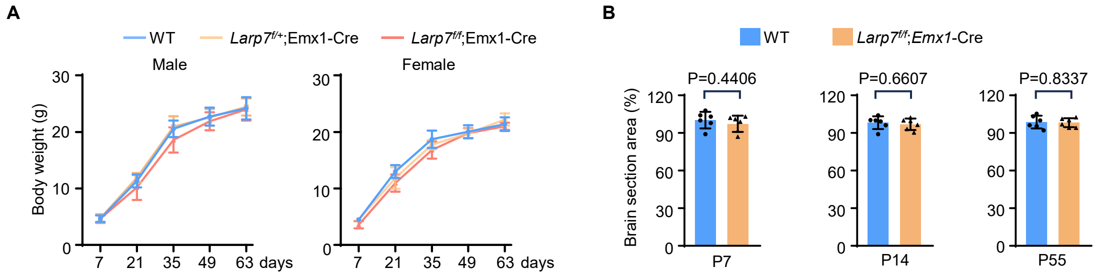

Appendix Figure S4. The body weight and brain size of wild-type mice are indistinguishable from those of Larp7f/f;Emx1-Cre mice.

(A) Body weight growth curve of wild-type (WT), heterozygotes (Larp7$^{f/+;}$Emx1-Cre), and homozygotes (Larp7$^{f/f}$;Emx1-Cre) from P7 to P63. Error bars, mean ± SEM. P-values, two-sided unpaired student's t test.

(B) Quantification of the brain size of wild-type (WT) and mutant (Larp7$^{f/f}$;Emx1-Cre) mice at P7 (left), P14 (middle) and P55 (right). The mean size of the whole brain in WT mice was set as 100% (n=6). Of note, unlike Larp7$^{f/f}$;nestin-Cre mice, the brain size of Larp7$^{f/f}$;Emx1-Cre mice was not reduced at P7 (n=6), P14 (n=6) or P55 (n=6). Error bars, mean ± SEM. P-values, two-sided unpaired student's t test.

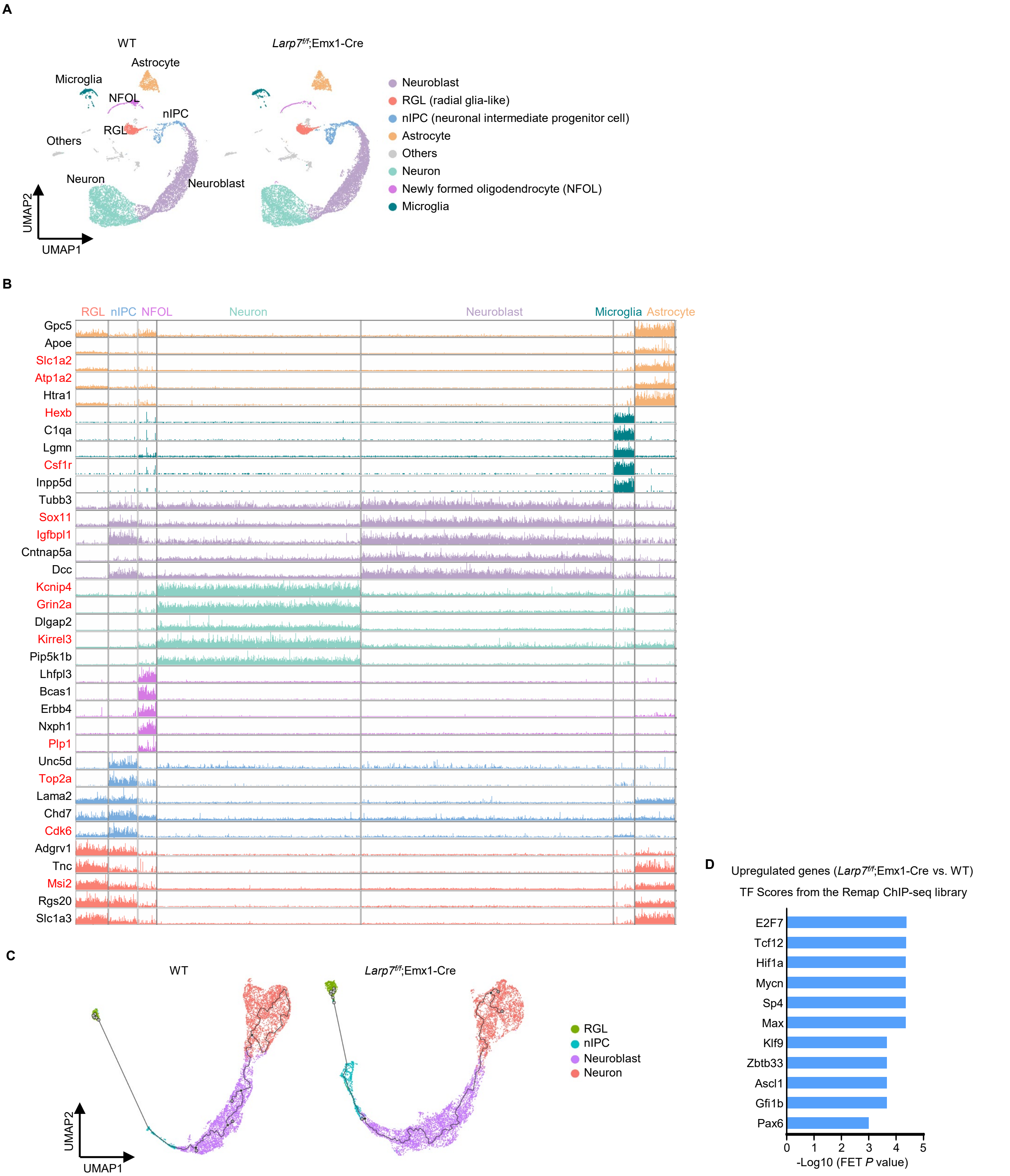

Appendix Figure S5. Single-cell RNA sequencing analysis of P14 dentate gyri.

(A) UMAP presentation of all cell types, identified by single-cell sequencing, from microdissected dentate gyrus from P14 wild-type (WT), and Larp7$^{f/f}$;Emx1-Cre mutant mice.

(B) Single-cell expression of enriched marker genes for each cell group identified by unbiased clustering (Seurat). Cluster-specific, differentially expressed genes (top 5 per cluster) were shown. Genes in red are established markers by previous studies. Annotated cell types are grouped by columns, and genes are organized by their associated cell types. Scale bars denote the expression level of indicated genes per cell.

(C) The differentiation trajectory inferred by Monocle 3.

(D) ChEA3 transcription factor (TF) analysis of upregulated genes in mutant nIPCs.

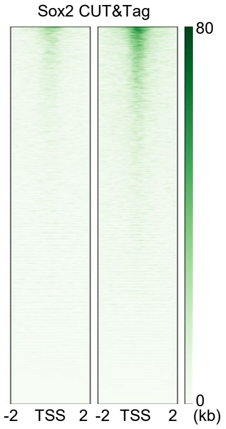

Appendix Figure S6. Heat-map representation of Sox2 CUT&Tag signals in wild-type and Larp7$^{f/f}$;Emx1-Cre neurospheres.

TSS, transcription start site.

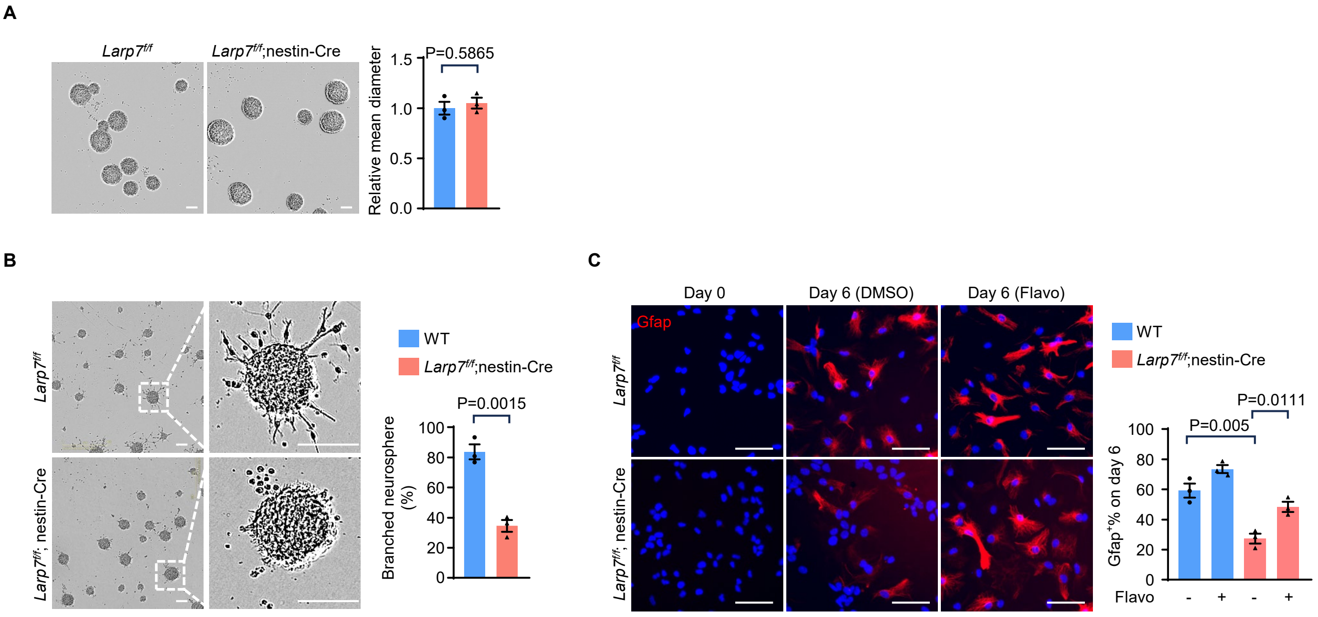

Appendix Figure S7. Neural stem and progenitor cells derived from Larp7$^{f/f}$;nestin-Cre P1 dentate gyrus exhibit enhanced self-renewal and compromised differentiation *in vitro*.

(A) Phase contrast images of self-renewing neurospheres, derived from the dentate gyrus of P1 wild-type (Larp7$^{f/f}$) and mutant (Larp7$^{f/f}$;nestin-Cre) mice, respectively. Representative results were shown (n=3). Error bars, mean ± SEM. P-values, two-sided unpaired student's t test. Scale bars: 100 μm.

(B) The self-renewal capacity of wild-type (Larp7$^{f/f}$) and mutant (Larp7$^{f/f}$;nestin-Cre) neurospheres under sub-optimal self-renewal culture condition. Representative results were shown (n=3). The percentage of neurospheres with a branched morphology, indicative of differentiation, was quantified. Error bars, mean ± SEM. P-values, two-sided unpaired student's t test. Scale bars: 100 μm.

(C) Differentiation of neurospheres for 6 days with or without P-TEFb inhibitor (flavopiridol). DMSO is the solvent of flavopiridol (Flavo). Representative images were shown (left panel) (n=3). Gfap immunofluorescence was used to quantify differentiated cells (right panel). Error bars, mean ± SEM. P-values, two-sided unpaired student's t test. Scale bars: 100 μm.

Phenotypic Category	Human *LARP7* Mutation Patients	Variant 1	Variant 2	*Larp7*$^{f/f}$;nestin-Cre Mice	*Larp7*$^{f/f}$;Emx1-Cre Mice *Hexim1*;Emx1-Cre Mice
**Inheritance**	Autosomal recessive[1]	/	/	Autosomal recessive	Autosomal recessive
**Height/Body length**	Short stature	c.1173T>A (p.Y391*)[2]	c.1173T>A (p.Y391*)	Reduced body length (fully penetrant)	No change
		c.1653_1654del (p.G553fs)[2]	c.1653_1654del (p.G553fs)		
		c.646+3_646+6del[2]	c.646+3_646+6del		
		c.1173T>A (p.Y391*)[2]	c.1653_1654del (p.G553fs)		
		c.213_214dup (p.S72fs)[3]	c.651_655del (p.(K219fs)		
		c.1070_1073del (p.R357fs)[4]	c.1070_1073del (p.R357fs)		
		c.1091_1094del (p.K364fs)[5]	c.1091_1094del (p.K364fs)		
		c.1669-1_1671del[6]	c.834dup (p.R279fs)		
		c.503_504dup (p.A169fs)[7]	c.503_504dup (p.A169fs)		
		c.892_895dup (p.S299fs)[8]	c.1087_1091del (p.H363fs)		
		c.1024_1030dup (p.T344fs)[4]	c.1024_1030dup (p.T344fs)		
**Weight**	Low body weight	c.1173T>A (p.Y391*)[2]	c.1173T>A (p.Y391*)	Reduced body weight (both sexes)	No change
		c.1653_1654del (p.G553fs)[2]	c.1653_1654del (p.G553fs)		
		c.646+3_646+6del[2]	c.646+3_646+6del		
		c.1173T>A (p.Y391*)[2]	c.1653_1654del (p.G553fs)		
		c.213_214dup (p.S72fs)[3]	c.651_655del (p.K219fs)		
		c.1091_1094del (p.K364fs)[5]	c.1091_1094del (p.K364fs)		
		c.1669-1_1671del[6]	c.834dup (p.R279fs)		
		c.503_504dup (p.A169fs)[7]	c.503_504dup (p.A169fs)		
		c.892_895dup (p.S299fs)[8]	c.1087_1091del (p.H363fs)		
		c.1024_1030dup (p.T344fs)[4]	c.1024_1030dup (p.T344fs)		
**Head**	Microcephaly	c.1173T>A (p.Y391*)[2]	c.1173T>A (p.Y391*)	Disproportionately reduced dentate gyrus size during neonatal period	No overall brain size reduction, but reduced dentate gyrus
		c.1653_1654del (p.G553fs)[2]	c.1653_1654del (p.G553fs)		
		c.646+3_646+6del[2]	c.646+3_646+6del		
		c.1173T>A (p.Y391*)[2]	c.1653_1654del (p.G553fs)		
		c.213_214dup (p.S72fs)[3]	c.651_655del (p.K219fs)		
		c.1070_1073del (p.R357fs)[4]	c.1070_1073del (p.R357fs)		
		c.1091_1094del (p.K364fs)[5]	c.1091_1094del (p.K364fs)		
		c.1669-1_1671del[6]	c.834dup (p.R279fs)		
		c.503_504dup (p.A169fs)[7]	c.503_504dup (p.A169fs)		
		c.892_895dup (p.S299fs)[8]	c.1087_1091del (p.H363fs)		
		c.1024_1030dup (p.T344fs)[4]	c.1024_1030dup (p.T344fs)		
**Central Nervous System**	Intellectual disability	c.832A>T (p.K278*)[9]	c.832A>T (p.K278*)	Impaired spatial learning and memory	Impaired spatial learning and memory
		c.1173T>A (p.Y391*)[2]	c.1173T>A (p.Y391*)		
		c.646+5G>C[10]	c.834dup (p.R279fs)		
		c.349del (p.E117fs)[11]	c.620_646+25del (p.P208_E216del)		
		c.1653_1654del (p.G553fs)[2]	c.1653_1654del (p.G553fs)		
		c.646+3_646+6del[2]	c.646+3_646+6del		
		c.1173T>A (p.Y391*)[2]	c.1653_1654del (p.G553fs)		
		c.827dup (p.K277fs)[10]	c.827dup (p.K277fs)		
		c.1024_1030dup (p.T344fs)[4,10]	c.1024_1030dup (p.T344fs)		
		c.1091_1094del (p.K364fs)[10]	c.1091_1094del (p.K364fs)		
		c.756_757del (p.R253fs)[10]	c.756_757del (p.R253fs)		
		c.503_504dup (p.A169fs)[7,10]	c.503_504dup (p.A169fs)		
		c.213_214dup (p.S72fs)[3]	c.651_655del (p.(K219fs)		
		c.1070_1073del (p.R357fs)[4]	c.1070_1073del (p.R357fs)		
		c.1091_1094del (p.K364fs)[5]	c.1091_1094del (p.K364fs)		
		c.1669-1_1671del[6]	c.834dup (p.R279fs)		
		c.892_895dup (p.S299fs)[8]	c.1087_1091del (p.(H363fs)		
**Behavioral Psychiatric Manifestations**	Severe anxiety	c.1173T>A (p.Y391*)[2]	c.1653_1654del (p.G553fs)	Significantly increased anxiety-related behaviors	Significantly increased anxiety-related behaviors
		c.213_214dup (p.S72fs)[3,10]	c.651_655del (p.(K219fs)		
		c.1091_1094del (p.K364fs)[10]	c.1091_1094del (p.K364fs)		
**Other Features**	/	/	/	Normal lifespan	Normal lifespan

Appendix Figure S8. Comparison of features in Alazami patients with phenotypes in *Larp7* and *Hexim1* knockout mice in the present study.

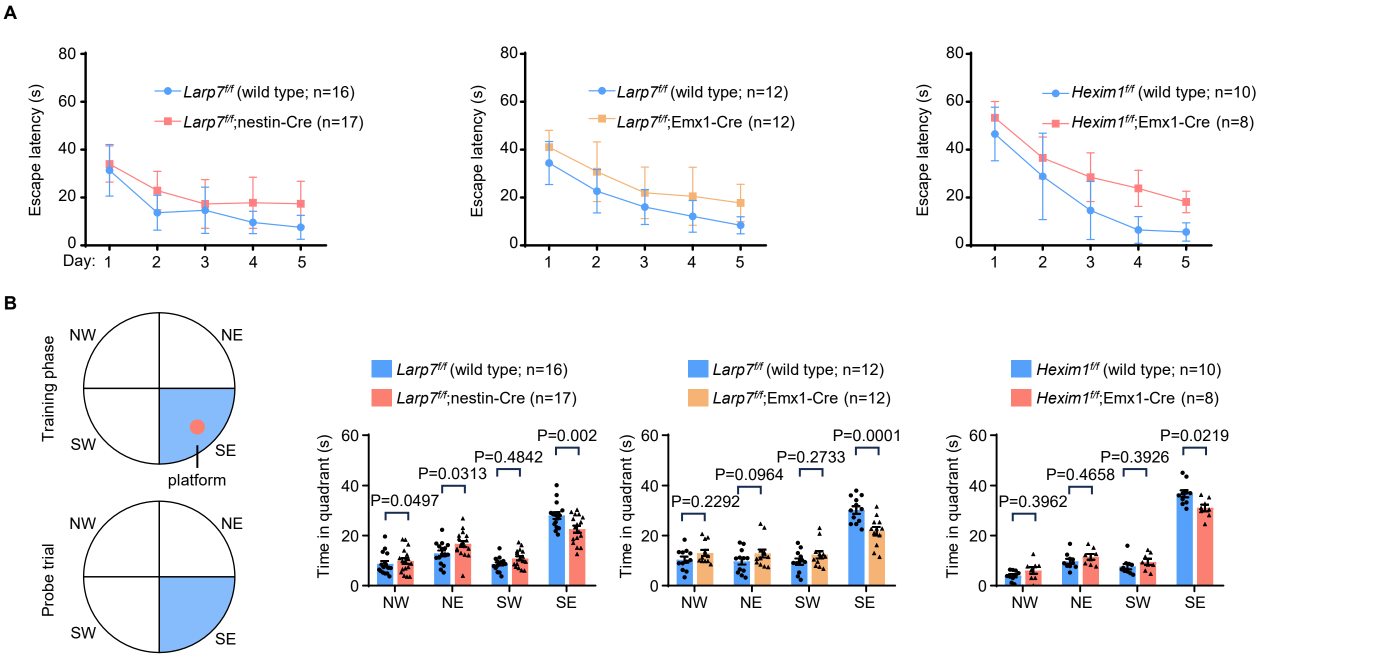

Appendix Figure S9. The training curves during the training phase, and the time in target quadrant during the probe trial of the Morris water maze test.

(A) The training curves for mice of indicated genotypes to locate the hidden platform during the 5-day training phase of the Morris water maze test.

(B) The time for mice of indicated genotypes to spend in target quadrant during the probe trial of the Morris water maze test.

Reference:

1. Alazami AM, Al-Owain M, Alzahrani F, et al. Loss of function mutation in LARP7, chaperone of 7SK ncRNA, causes a syndrome of facial dysmorphism, intellectual disability, and primordial dwarfism. *Hum Mutat*. Oct 2012;33(10):1429-34. doi:10.1002/humu.22175

2. Al-Hinai A, Al-Hashmi S, Ganesh A, et al. Further phenotypic delineation of Alazami syndrome. *American journal of medical genetics Part A*. Aug 2022;188(8):2485-2490. doi:10.1002/ajmg.a.62778

3. Ling TT, Sorrentino S. Compound heterozygous variants in the LARP7 gene as a cause of Alazami syndrome in a Caucasian female with significant failure to thrive, short stature, and developmental disability. *American journal of medical genetics Part A*. Jan 2016;170a(1):217-9. doi:10.1002/ajmg.a.37396

4. Hollink IH, Alfadhel M, Al-Wakeel AS, et al. Broadening the phenotypic spectrum of pathogenic LARP7 variants: two cases with intellectual disability, variable growth retardation and distinct facial features. *Journal of human genetics*. Mar 2016;61(3):229-33. doi:10.1038/jhg.2015.134

5. Buisine-Sbraggia A, Thevenon J, Yauy K, et al. Exome Sequencing Detects Uniparental Disomy of Chromosome 4 Revealing a LARP7 Pathogenic Variant Responsible for Alazami Syndrome: A Case Report. *American journal of medical genetics Part A*. Mar 2025;197(3):e63891. doi:10.1002/ajmg.a.63891

6. Patalan M, Leśniak A, Bernatowicz K, et al. Patient with Phenylketonuria and Intellectual Disability-Problem Not Always Caused Exclusively by Insufficient Metabolic Control (Coexistence of PKU and Alazami Syndrome). *Int J Environ Res Public Health*. Feb 24 2022;19(5)doi:10.3390/ijerph19052574

7. Imbert-Bouteille M, Mau Them FT, Thevenon J, et al. LARP7 variants and further delineation of the Alazami syndrome phenotypic spectrum among primordial dwarfisms: 2 sisters. *Eur J Med Genet*. Mar 2019;62(3):161-166. doi:10.1016/j.ejmg.2018.07.003

8. Ivanovski I, Caraffi SG, Magnani E, et al. Alazami syndrome: the first case of papillary thyroid carcinoma. *Journal of human genetics*. Jan 2020;65(2):133-141. doi:10.1038/s10038-019-0682-5

9. Elmas M, Yıldız H, Erdoğan M, Gogus B, Avcı K, Solak M. Comparison of clinical parameters with whole exome sequencing analysis results of autosomal recessive patients; a center experience. *Molecular biology reports*. Feb 2019;46(1):287-299. doi:10.1007/s11033-018-4470-7

10. Wojcik MH, Linnea K, Stoler JM, Rappaport L. Updating the neurodevelopmental profile of Alazami syndrome: Illustrating the role of developmental assessment in rare genetic disorders. *American journal of medical genetics Part A*. Aug 2019;179(8):1565-1569. doi:10.1002/ajmg.a.61189

11. Dateki S, Kitajima T, Kihara T, Watanabe S, Yoshiura KI, Moriuchi H. Novel compound heterozygous variants in the LARP7 gene in a patient with Alazami syndrome. *Human genome variation*. 2018;5:18014. doi:10.1038/hgv.2018.14

12. Madisen, L. *et al.* A robust and high-throughput Cre reporting and characterization system for the whole mouse brain. *Nat Neurosci* 13, 133-140 (2010).
