# Supplementary figures and images for: Enhanced P-TEFb activity compromises dentate gyrus neurogenesis in mice
Source: EMBO J. 2026 Mar 23;45(9):3102–23. doi: 10.1038/s44318-026-00752-w (PMC13144724; doi:10.1038/s44318-026-00752-w)

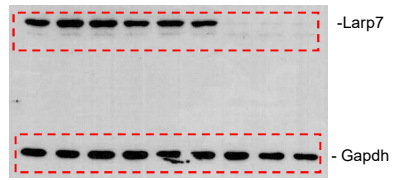

Supplement: Supplementary file 3 — Source data Fig. 1 [file 44318_2026_752_MOESM3_ESM.zip › Source Data for Figure 1/1B/western blot for 1B.pdf]

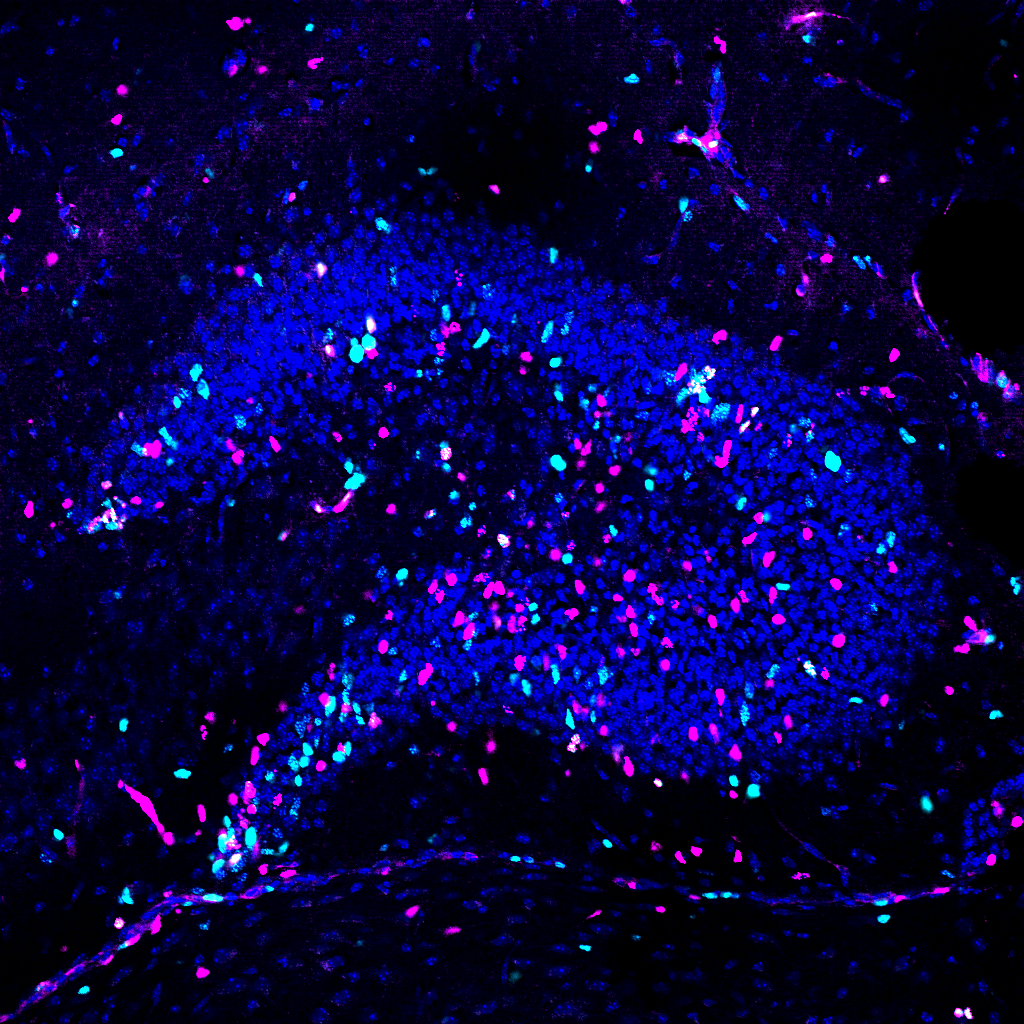

Supplement: Supplementary file 4 — Source data Fig. 2 [file 44318_2026_752_MOESM4_ESM.zip › Source Data for Figure 2/2C/Larp7ff;nestin-Cre.tif]

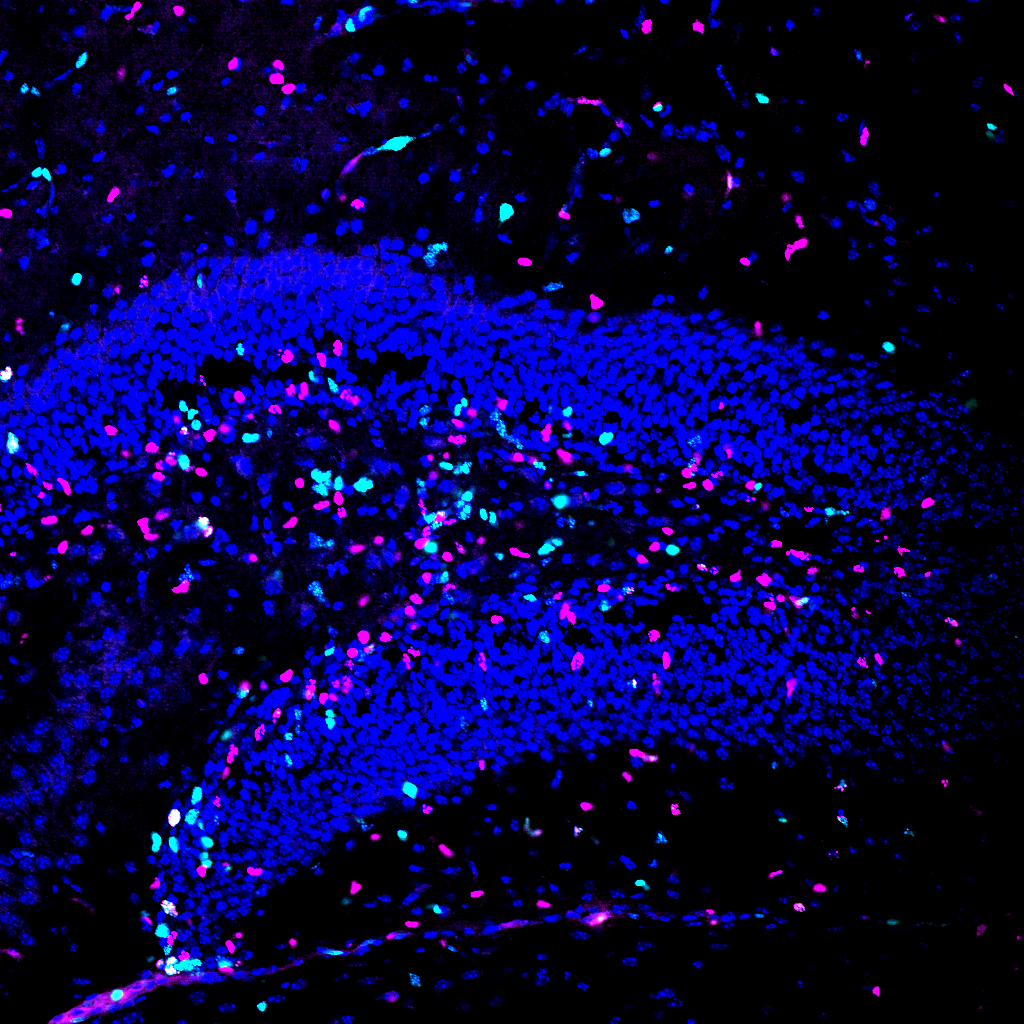

Supplement: Supplementary file 4 — Source data Fig. 2 [file 44318_2026_752_MOESM4_ESM.zip › Source Data for Figure 2/2C/WT.tif]

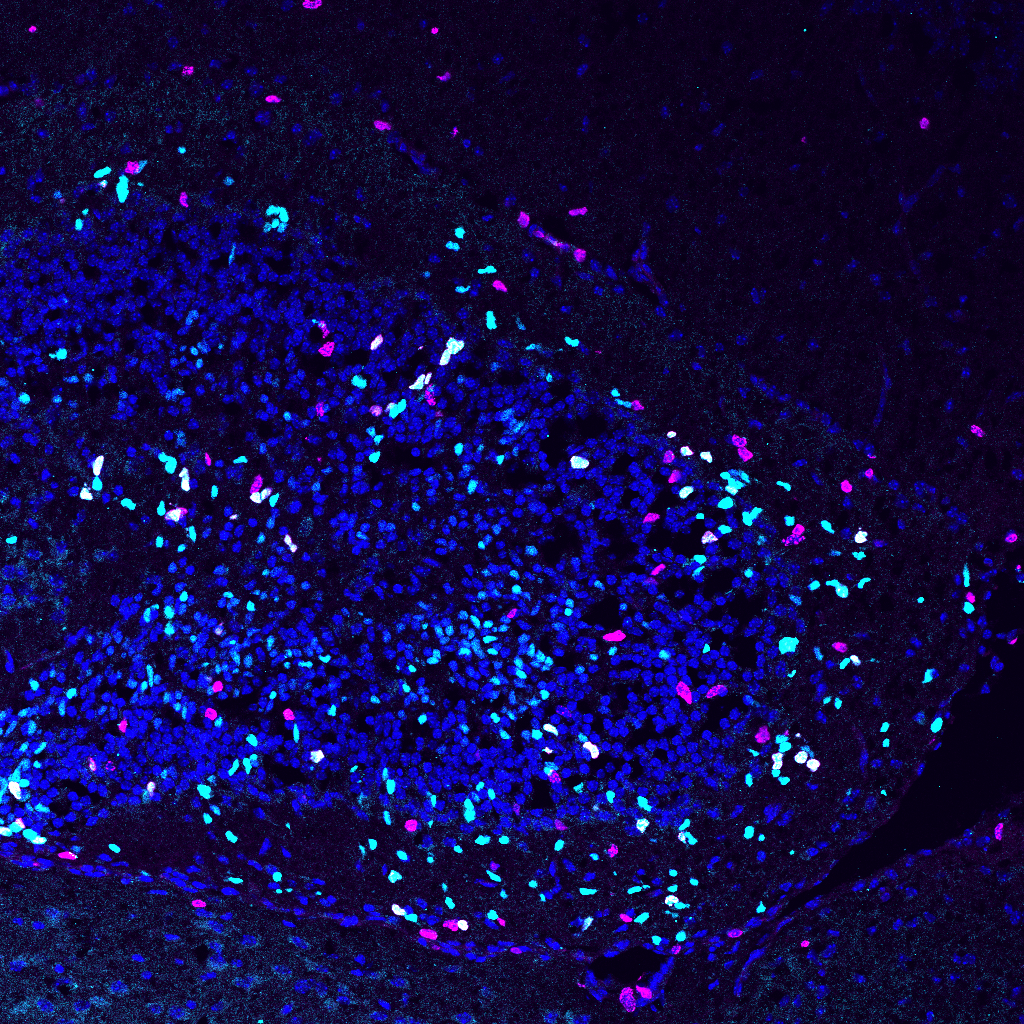

Supplement: Supplementary file 4 — Source data Fig. 2 [file 44318_2026_752_MOESM4_ESM.zip › Source Data for Figure 2/2D/Larp7ff;nestin-Cre.tif]

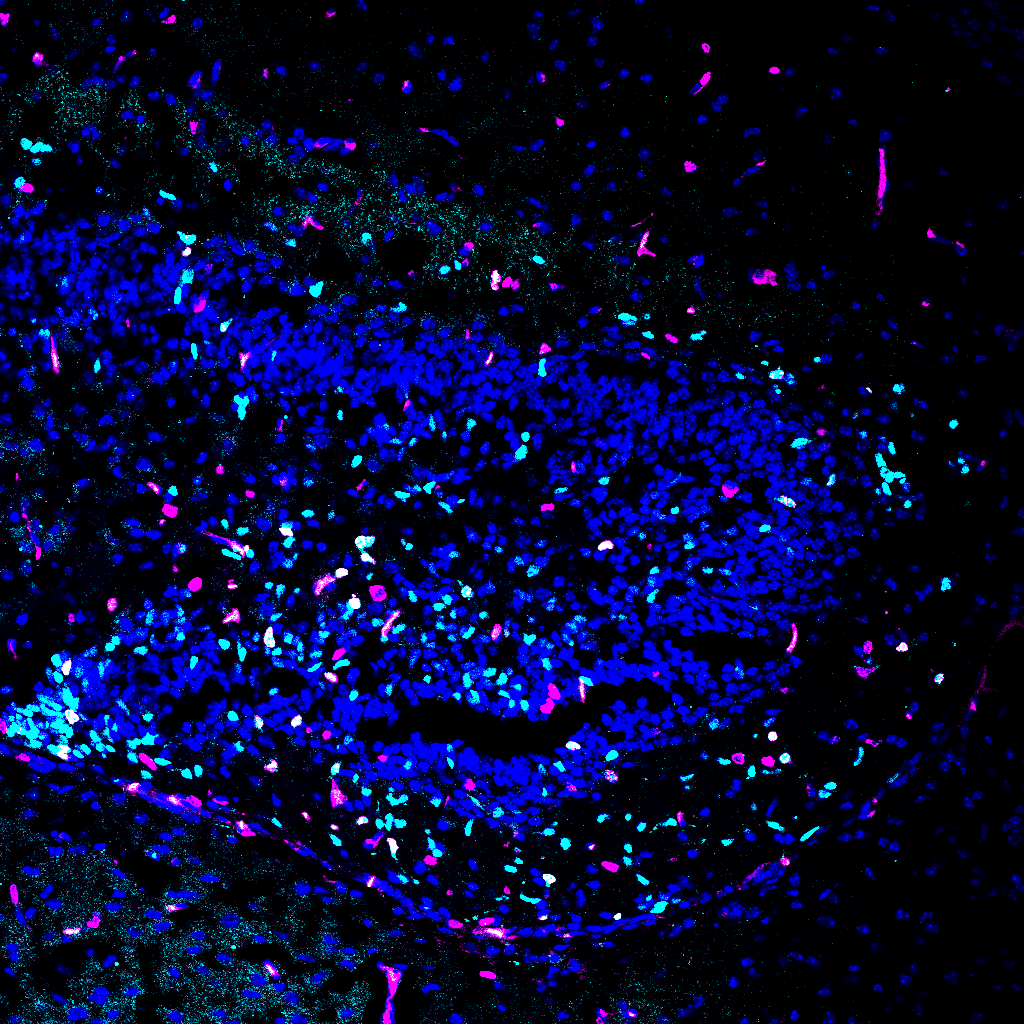

Supplement: Supplementary file 4 — Source data Fig. 2 [file 44318_2026_752_MOESM4_ESM.zip › Source Data for Figure 2/2D/WT.tif]

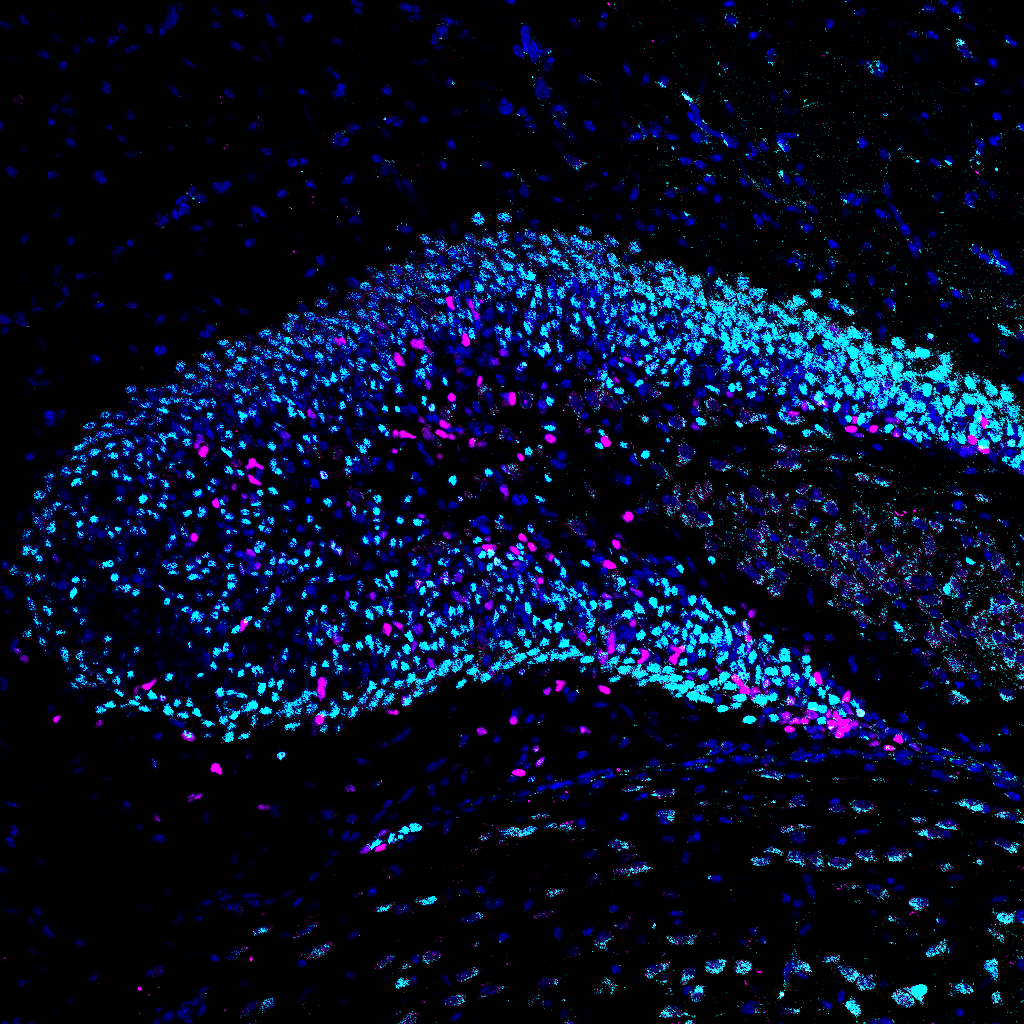

Supplement: Supplementary file 4 — Source data Fig. 2 [file 44318_2026_752_MOESM4_ESM.zip › Source Data for Figure 2/2E/Larp7ff;nestin-Cre.tif]

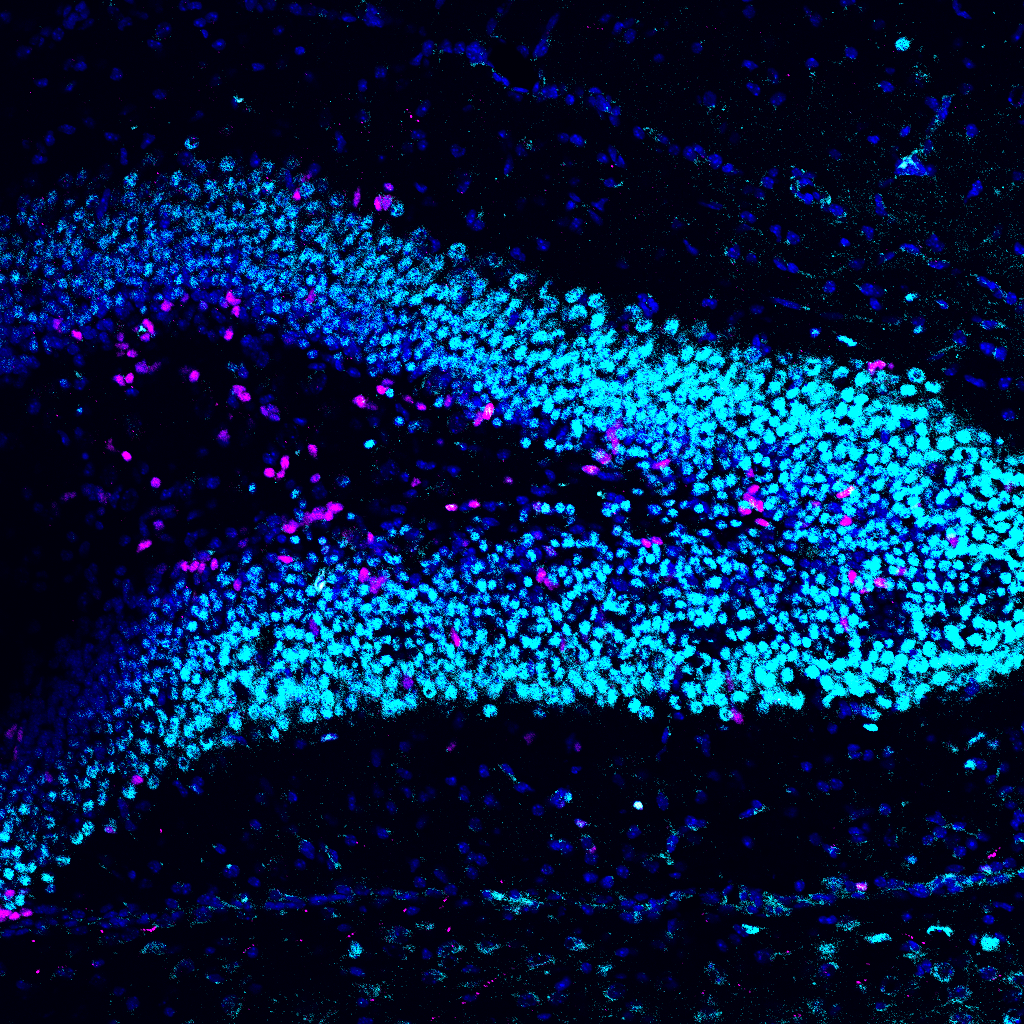

Supplement: Supplementary file 4 — Source data Fig. 2 [file 44318_2026_752_MOESM4_ESM.zip › Source Data for Figure 2/2E/WT.tif]

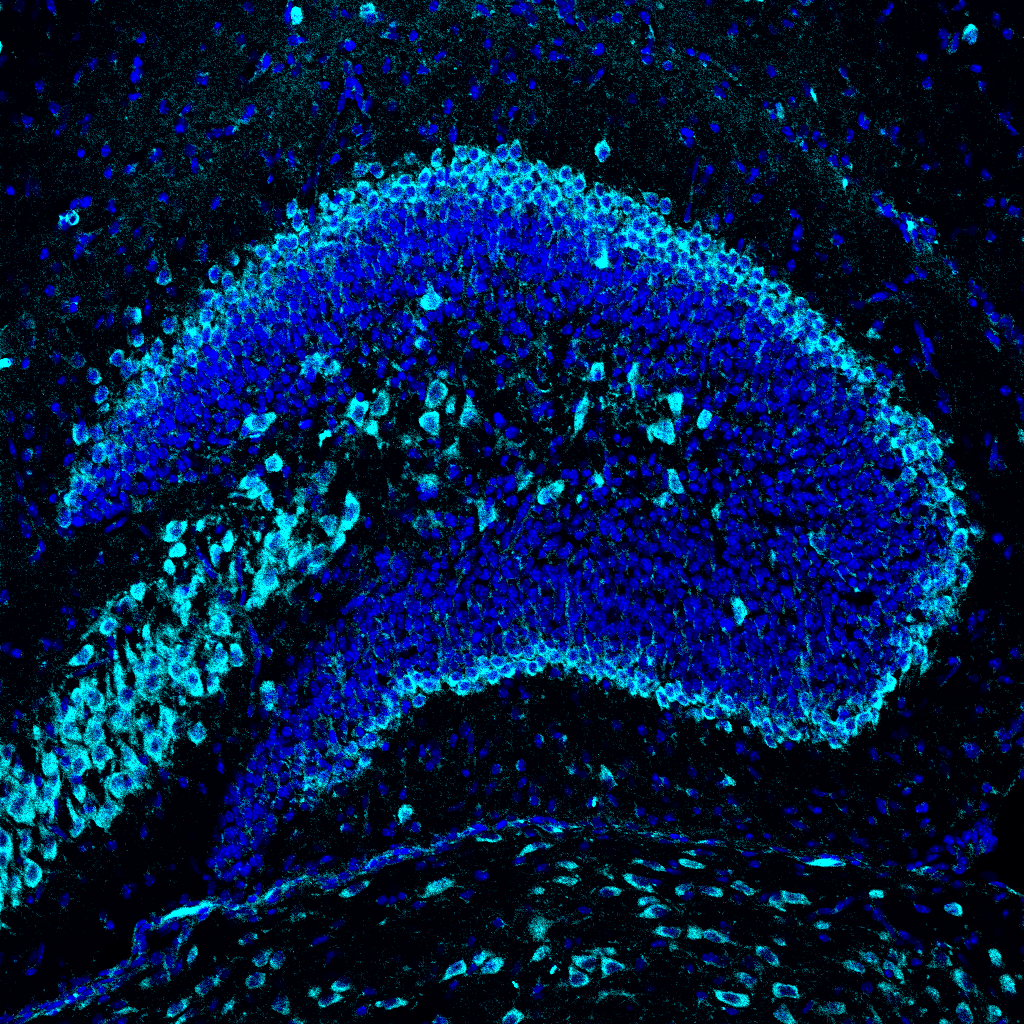

Supplement: Supplementary file 4 — Source data Fig. 2 [file 44318_2026_752_MOESM4_ESM.zip › Source Data for Figure 2/2F/Larp7ff;nestin-Cre.tif]

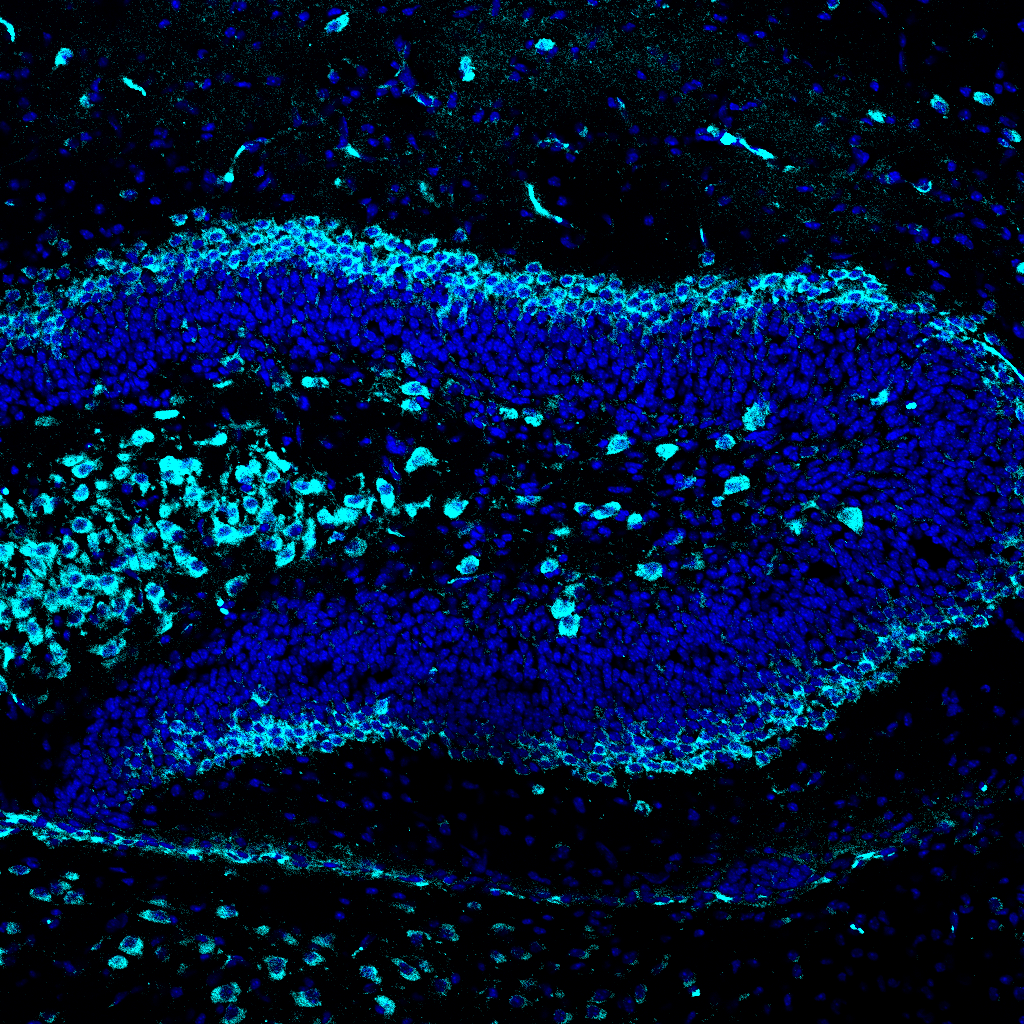

Supplement: Supplementary file 4 — Source data Fig. 2 [file 44318_2026_752_MOESM4_ESM.zip › Source Data for Figure 2/2F/WT.tif]

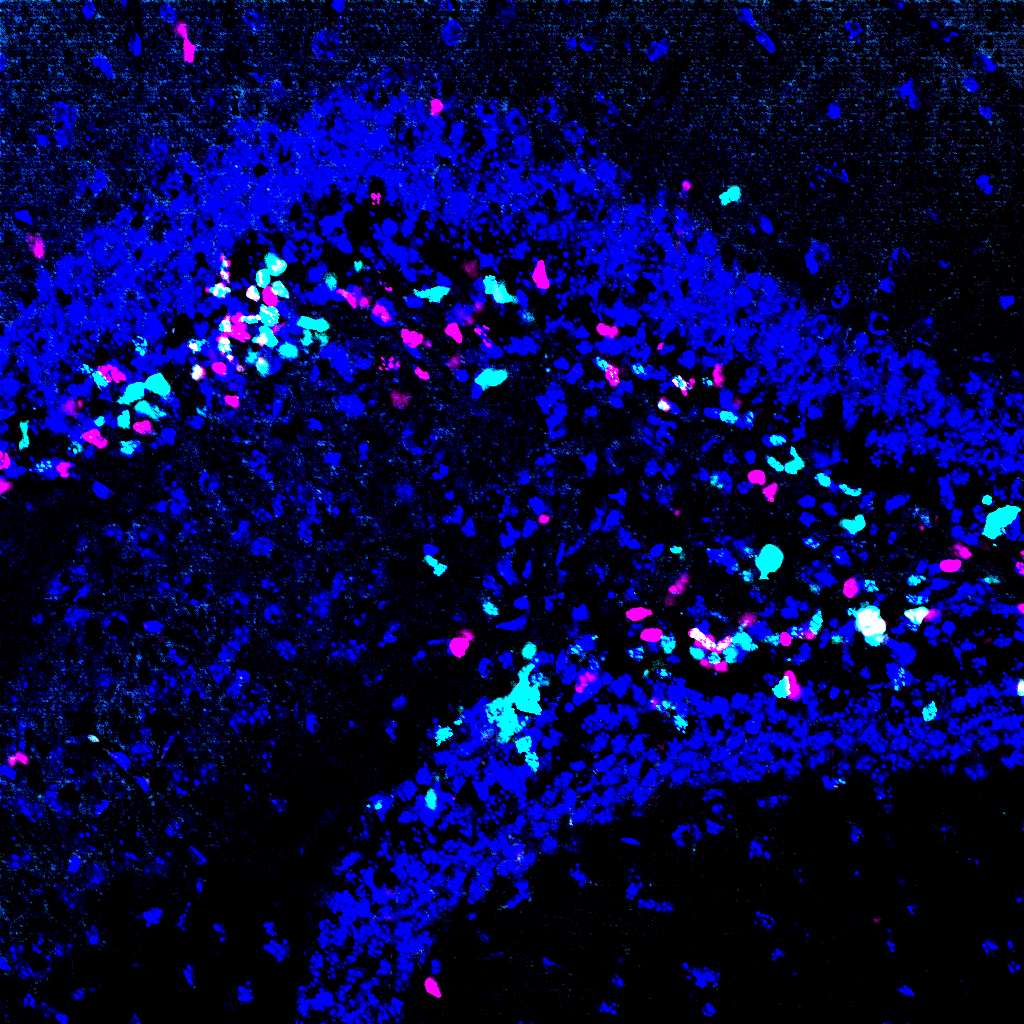

Supplement: Supplementary file 4 — Source data Fig. 2 [file 44318_2026_752_MOESM4_ESM.zip › Source Data for Figure 2/2G/Larp7ff;nestin-Cre.tif]

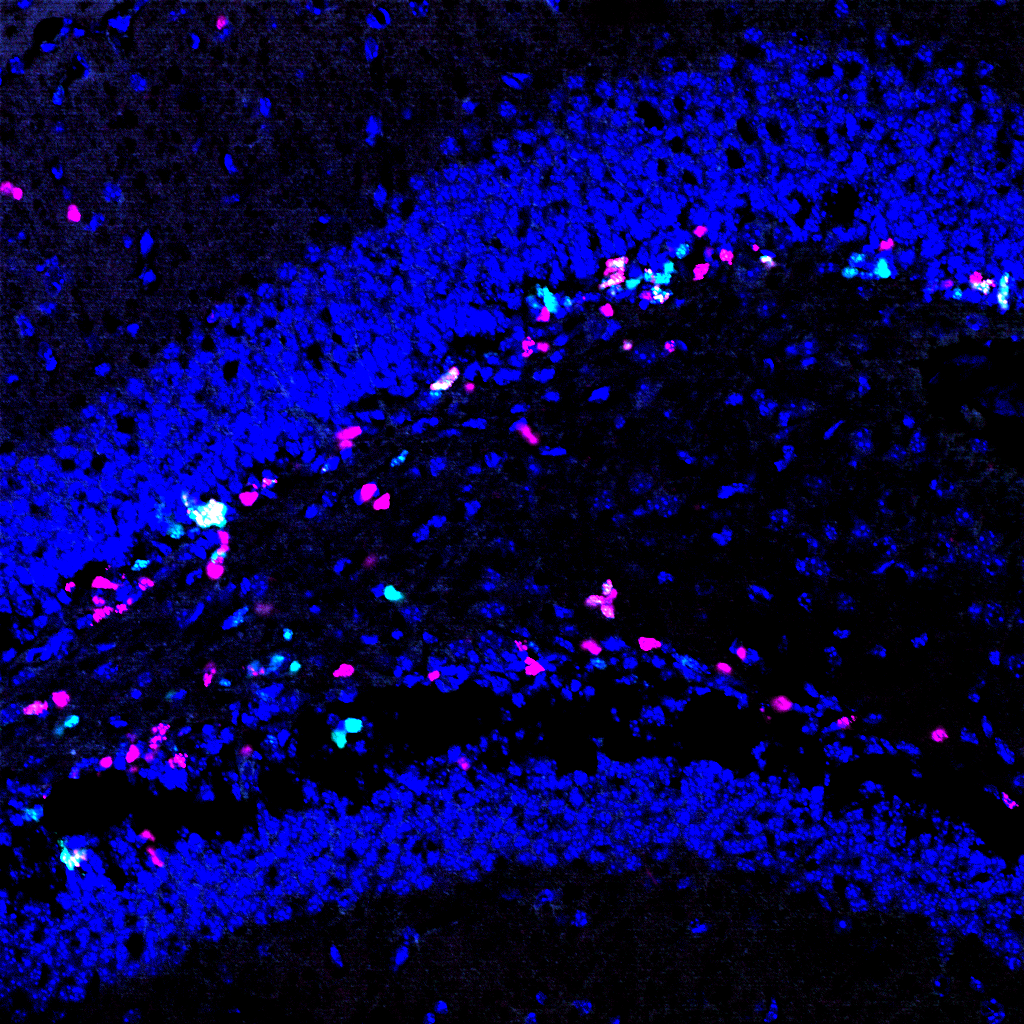

Supplement: Supplementary file 4 — Source data Fig. 2 [file 44318_2026_752_MOESM4_ESM.zip › Source Data for Figure 2/2G/WT.tif]

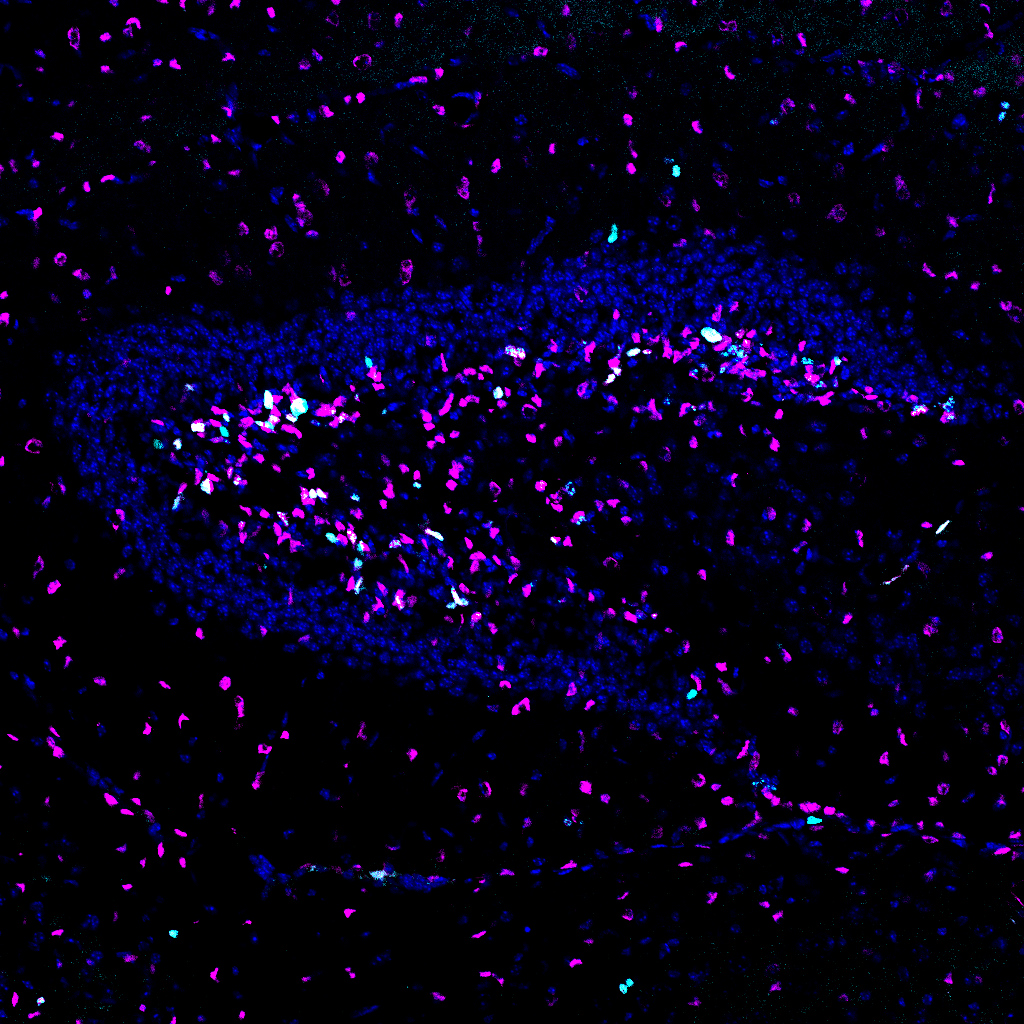

Supplement: Supplementary file 4 — Source data Fig. 2 [file 44318_2026_752_MOESM4_ESM.zip › Source Data for Figure 2/2H/Larp7ff;nestin-Cre.tif]

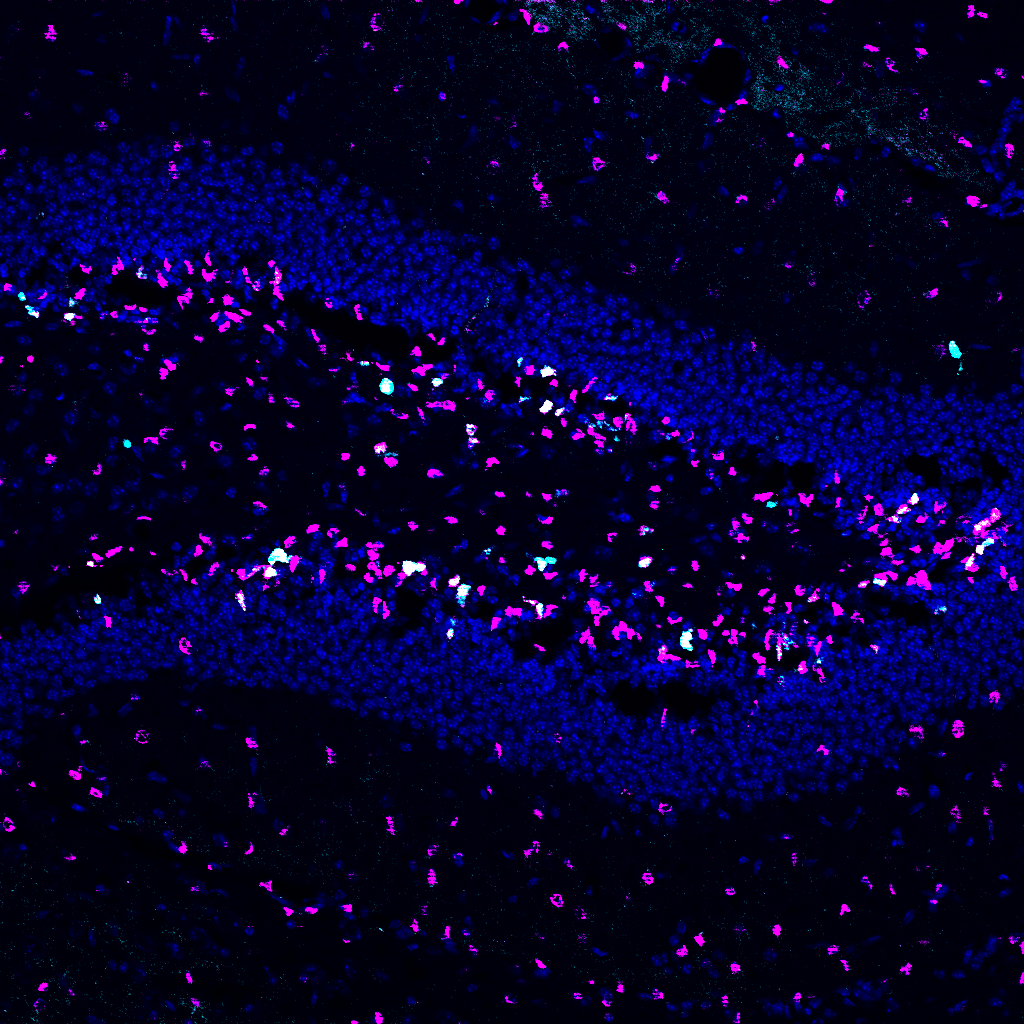

Supplement: Supplementary file 4 — Source data Fig. 2 [file 44318_2026_752_MOESM4_ESM.zip › Source Data for Figure 2/2H/WT.tif]

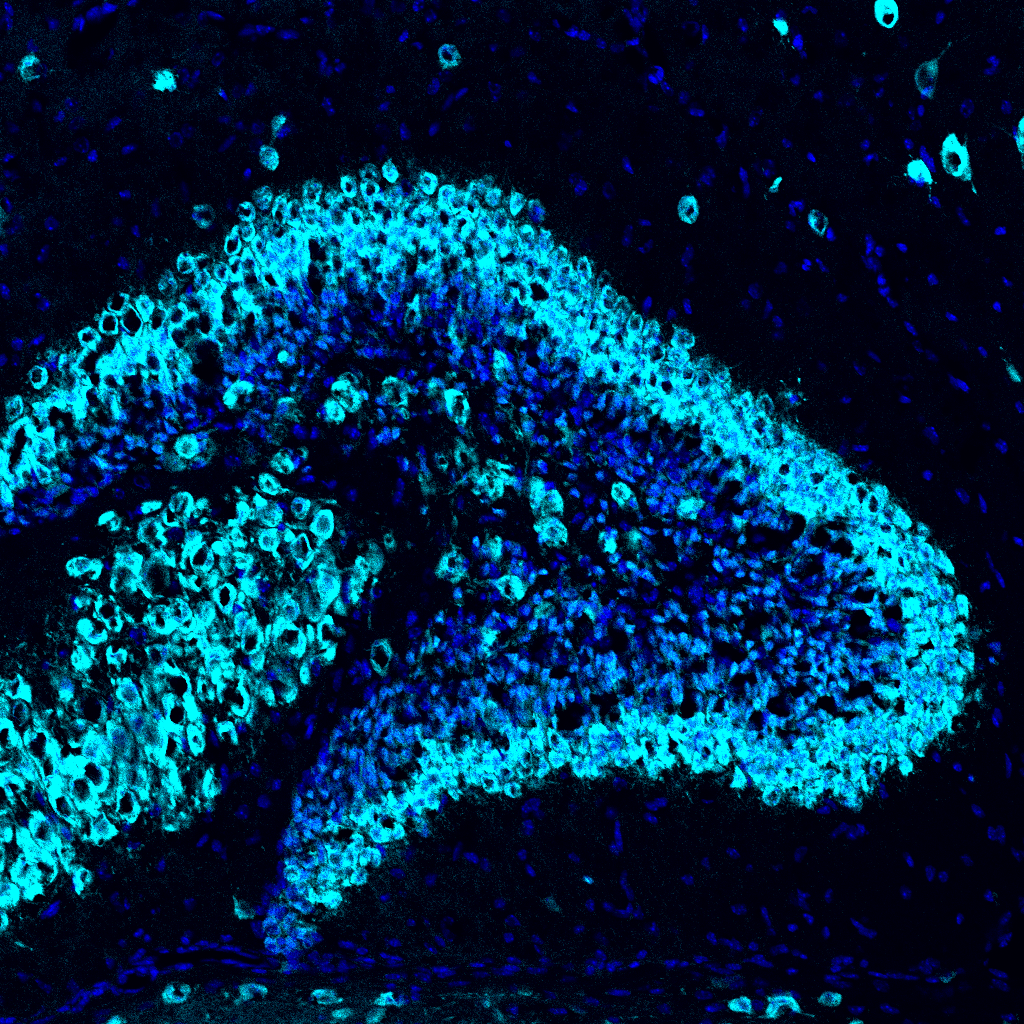

Supplement: Supplementary file 4 — Source data Fig. 2 [file 44318_2026_752_MOESM4_ESM.zip › Source Data for Figure 2/2I/Larp7ff;nestin-Cre.tif]

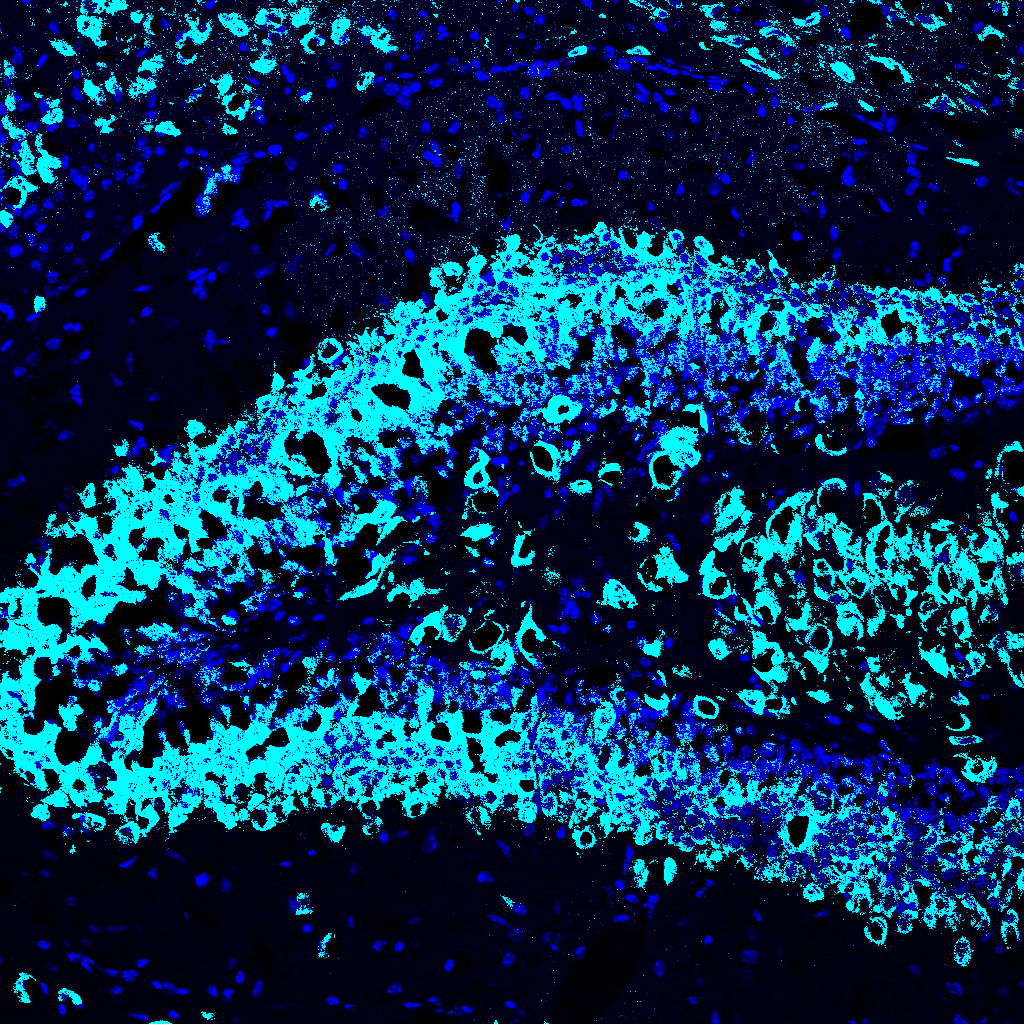

Supplement: Supplementary file 4 — Source data Fig. 2 [file 44318_2026_752_MOESM4_ESM.zip › Source Data for Figure 2/2I/WT.tif]

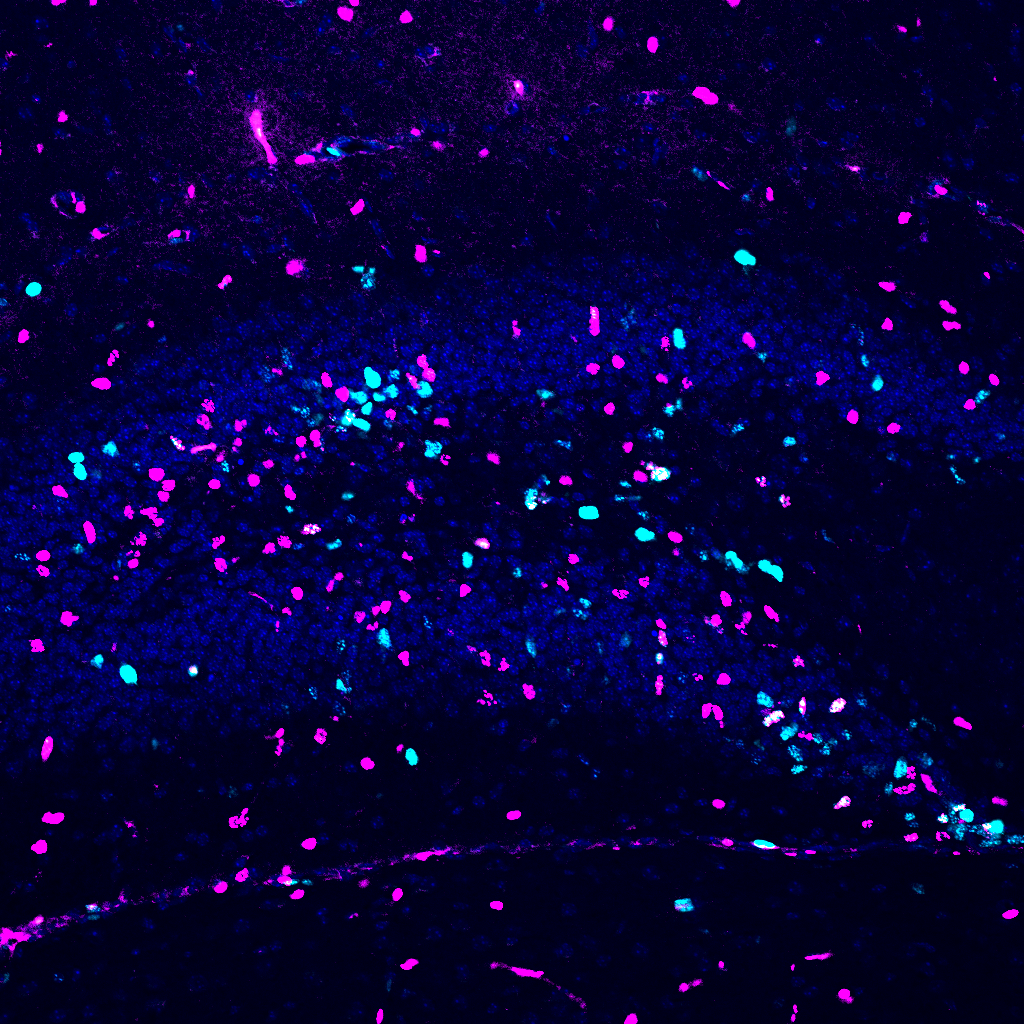

Supplement: Supplementary file 5 — Source data Fig. 3 [file 44318_2026_752_MOESM5_ESM.zip › Source Data for Figure 4/4D/Larp7ff;Emx1-Cre.tif]

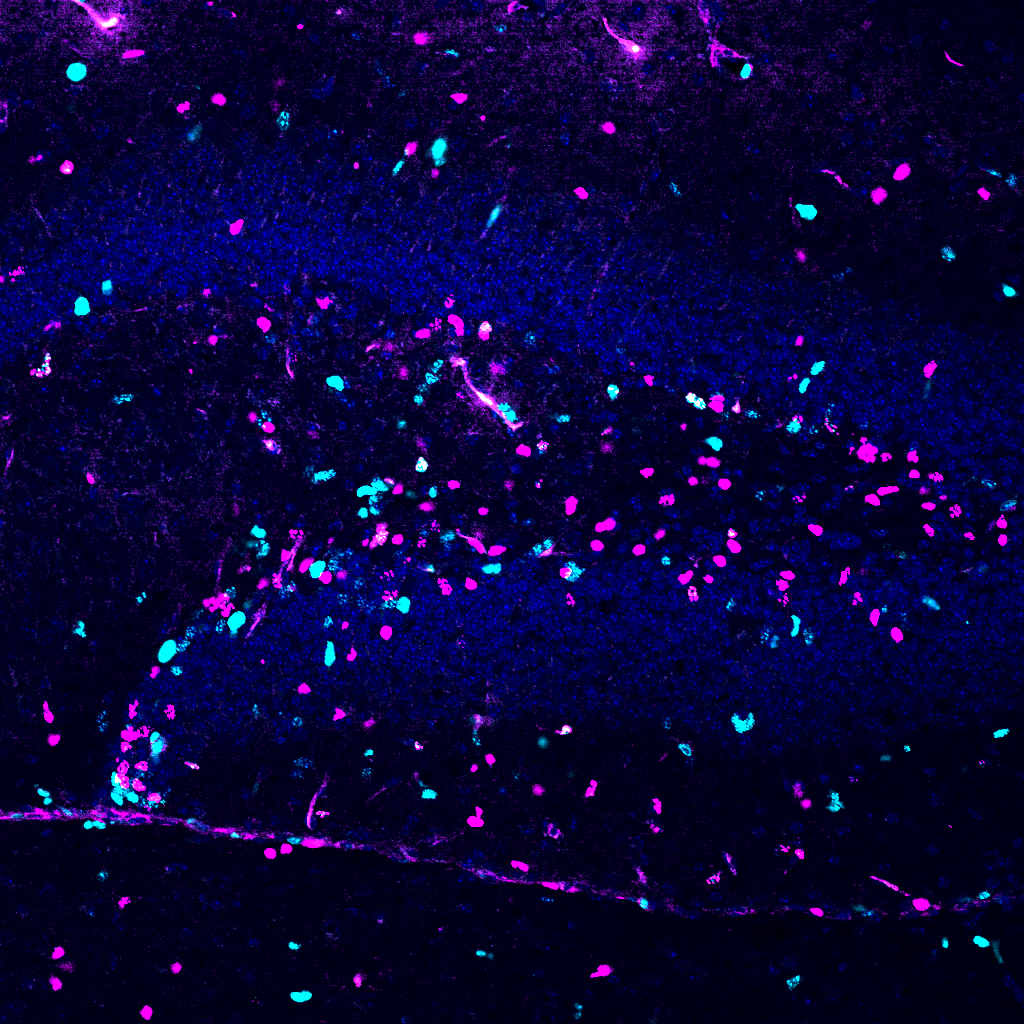

Supplement: Supplementary file 5 — Source data Fig. 3 [file 44318_2026_752_MOESM5_ESM.zip › Source Data for Figure 4/4D/WT.tif]

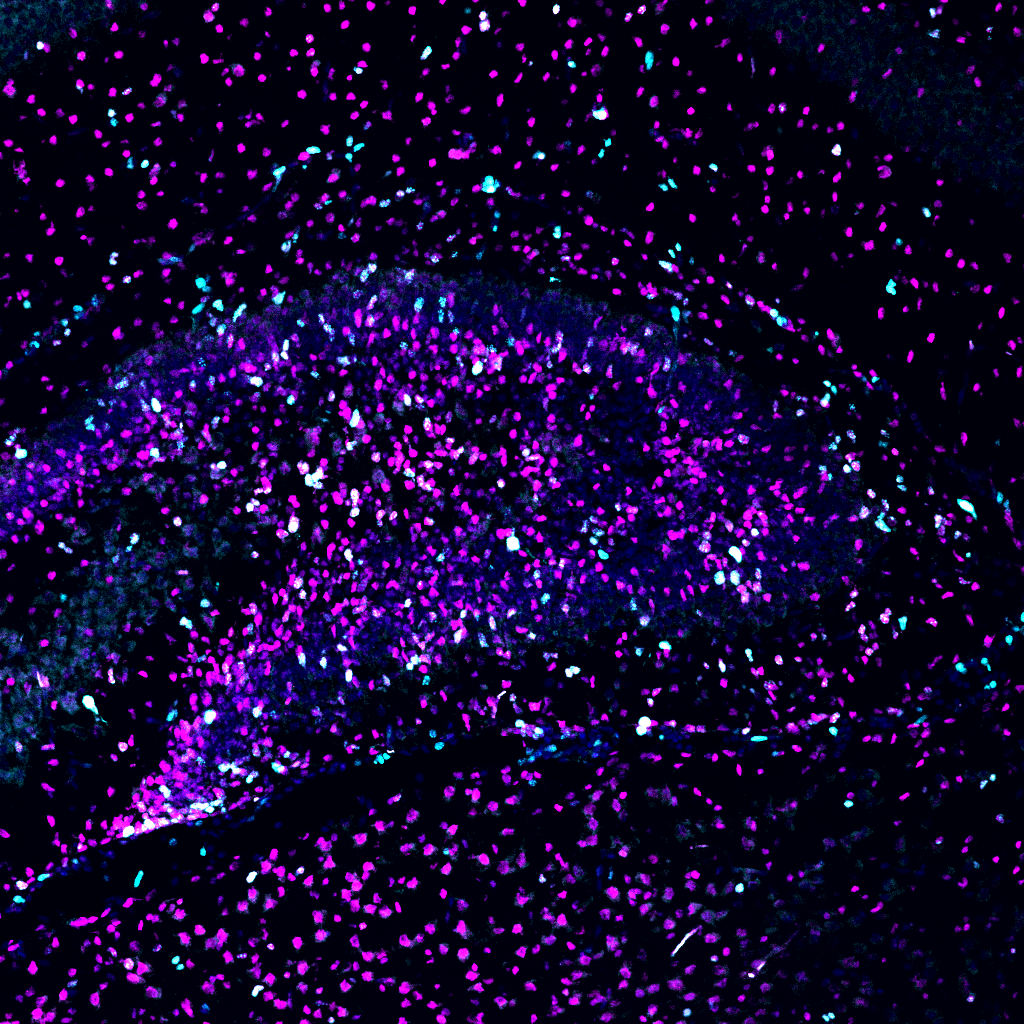

Supplement: Supplementary file 5 — Source data Fig. 3 [file 44318_2026_752_MOESM5_ESM.zip › Source Data for Figure 4/4E/Larp7ff;Emx1-Cre.tif]

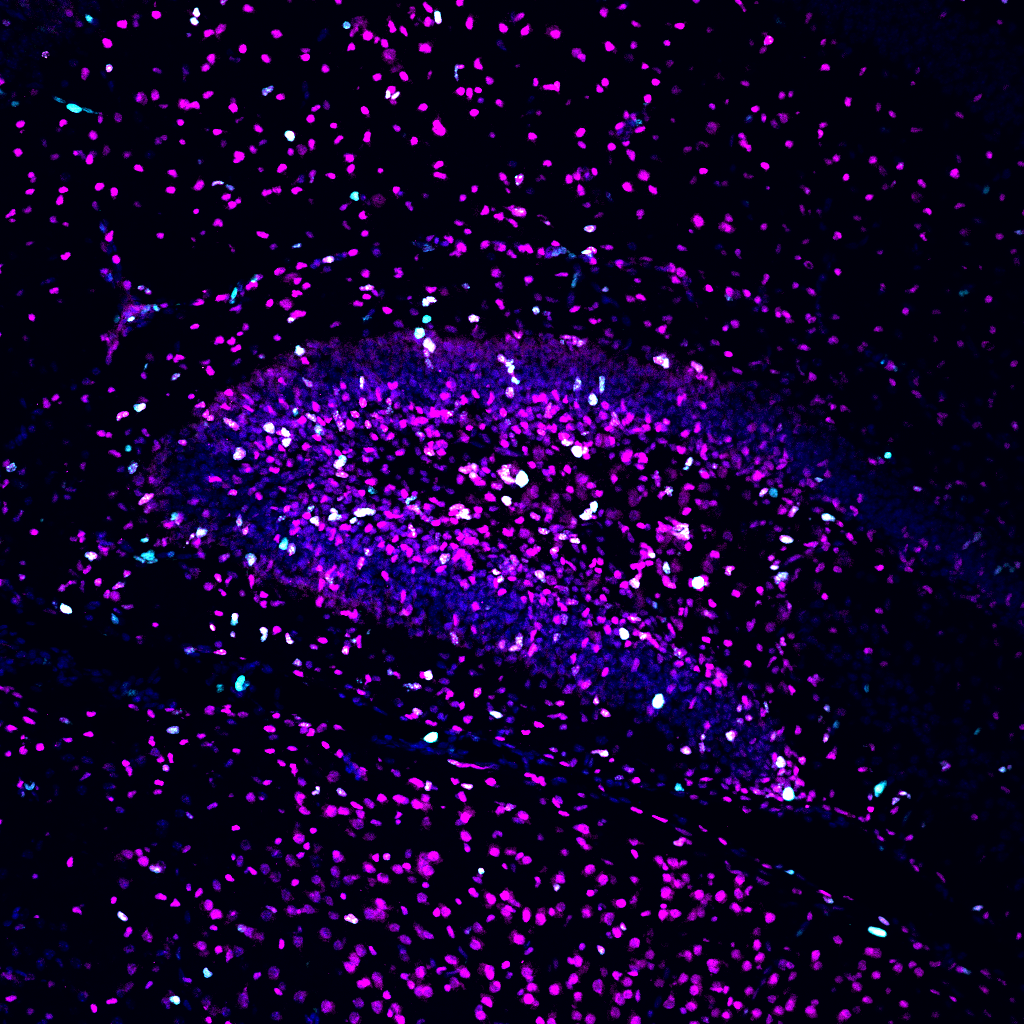

Supplement: Supplementary file 5 — Source data Fig. 3 [file 44318_2026_752_MOESM5_ESM.zip › Source Data for Figure 4/4E/WT.tif]

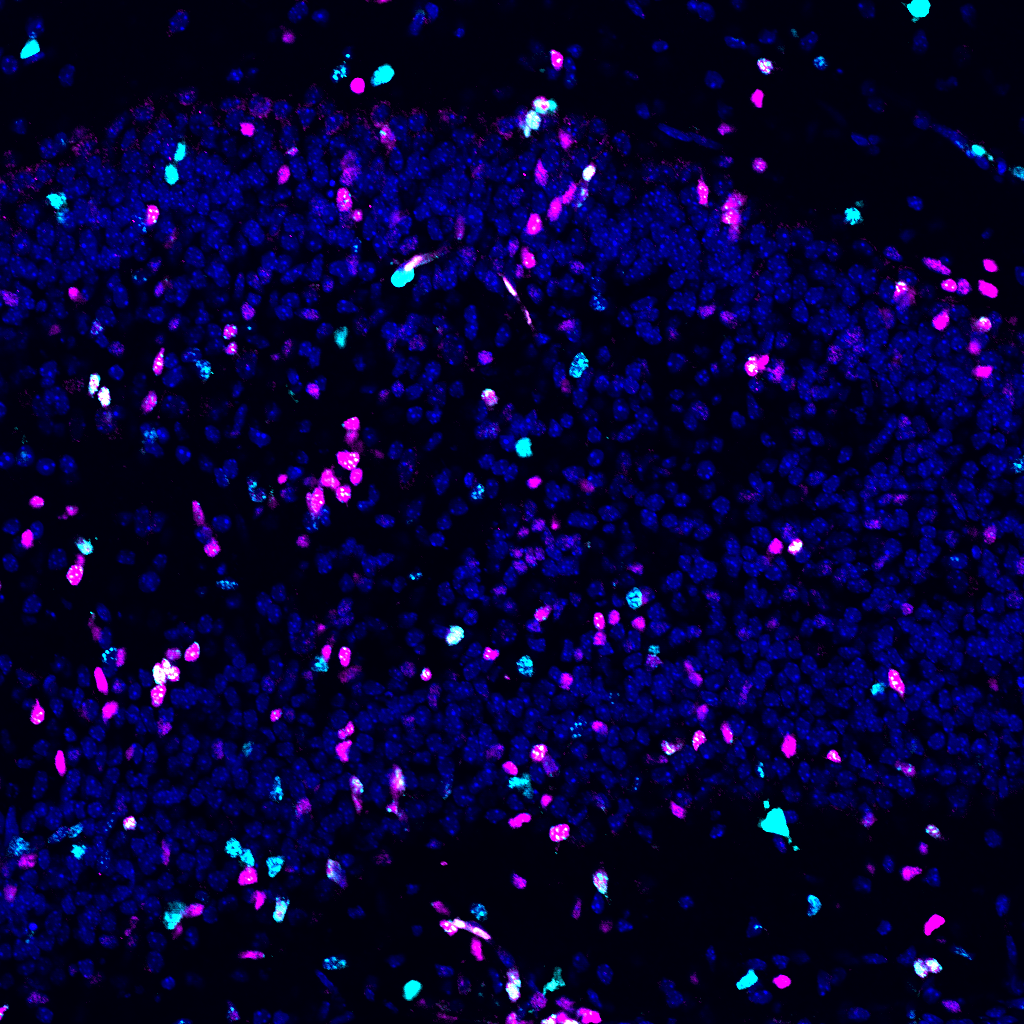

Supplement: Supplementary file 5 — Source data Fig. 3 [file 44318_2026_752_MOESM5_ESM.zip › Source Data for Figure 4/4F/Larp7ff;Emx1-Cre.tif]

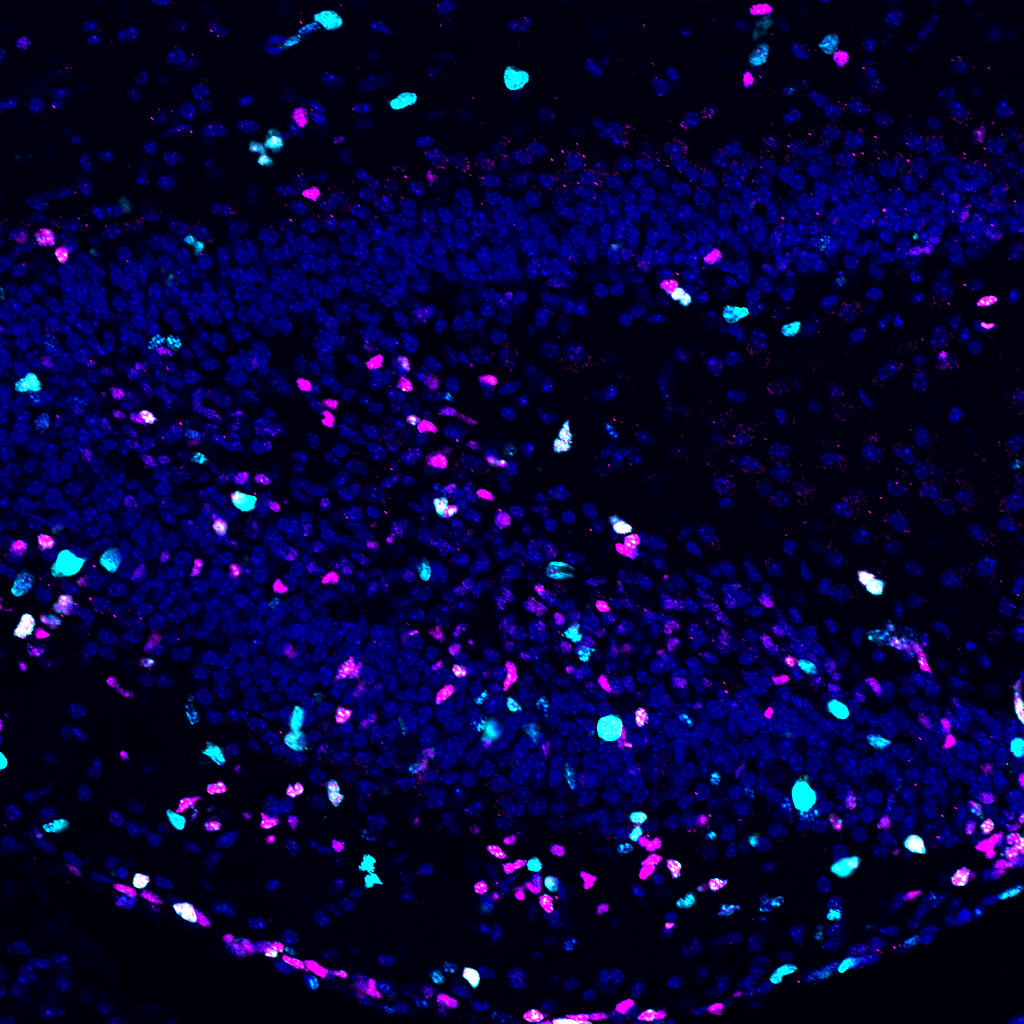

Supplement: Supplementary file 5 — Source data Fig. 3 [file 44318_2026_752_MOESM5_ESM.zip › Source Data for Figure 4/4F/WT.tif]

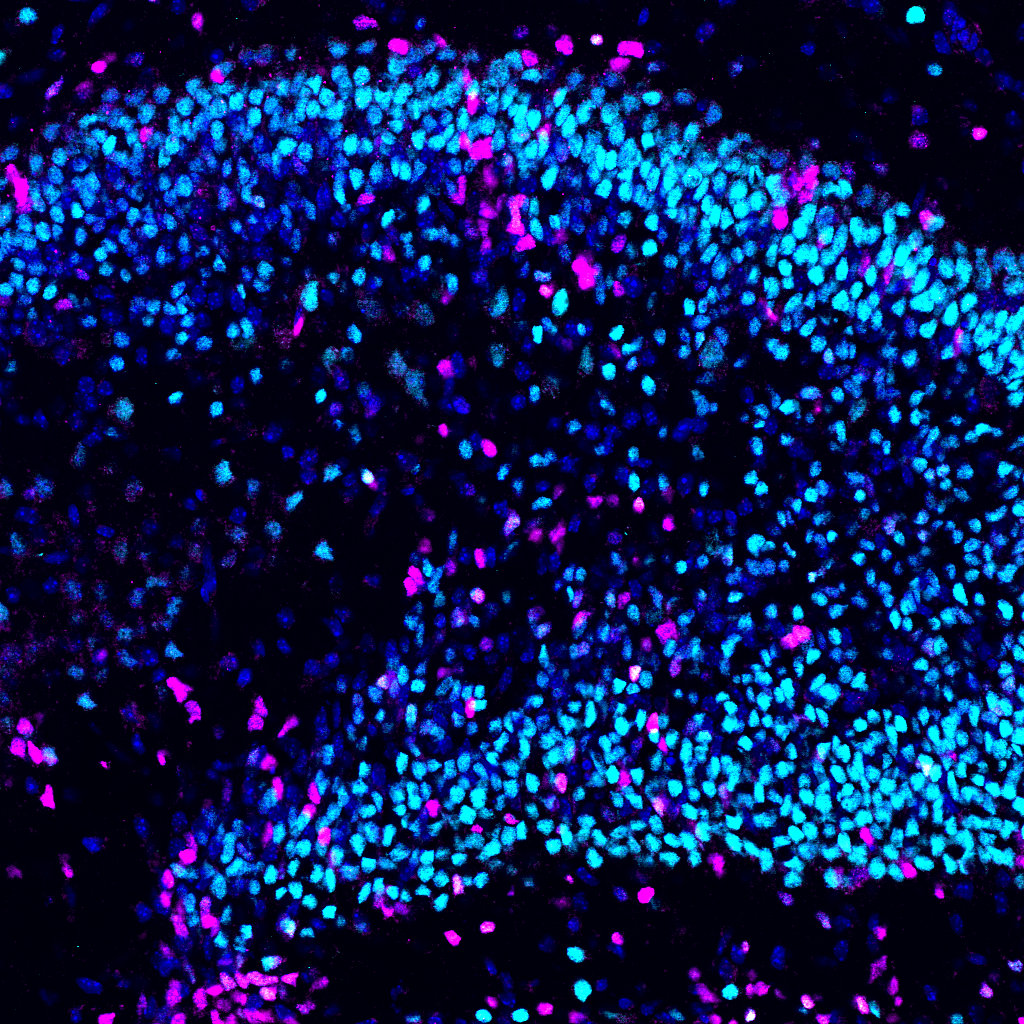

Supplement: Supplementary file 5 — Source data Fig. 3 [file 44318_2026_752_MOESM5_ESM.zip › Source Data for Figure 4/4G/Larp7ff;Emx1-Cre.tif]

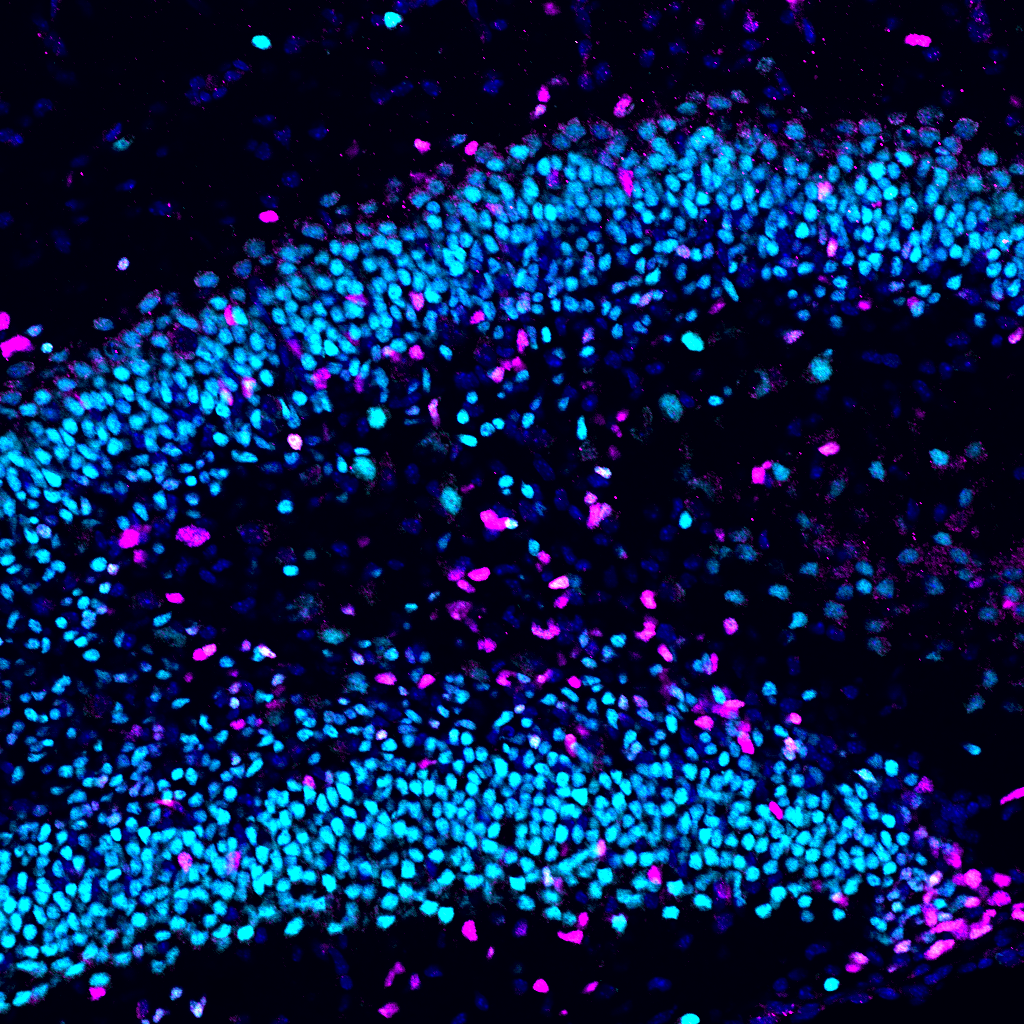

Supplement: Supplementary file 5 — Source data Fig. 3 [file 44318_2026_752_MOESM5_ESM.zip › Source Data for Figure 4/4G/WT.tif]

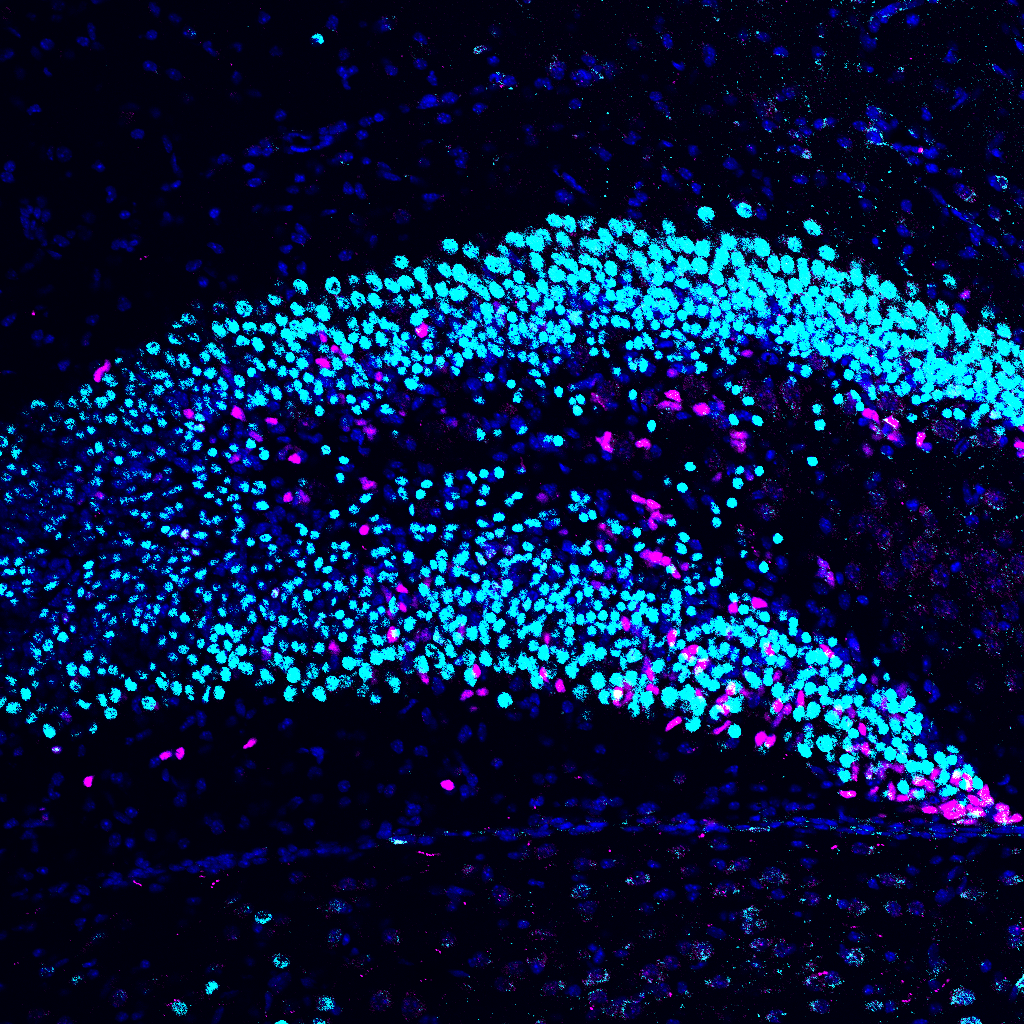

Supplement: Supplementary file 5 — Source data Fig. 3 [file 44318_2026_752_MOESM5_ESM.zip › Source Data for Figure 4/4H/Larp7ff;Emx1-Cre.tif]

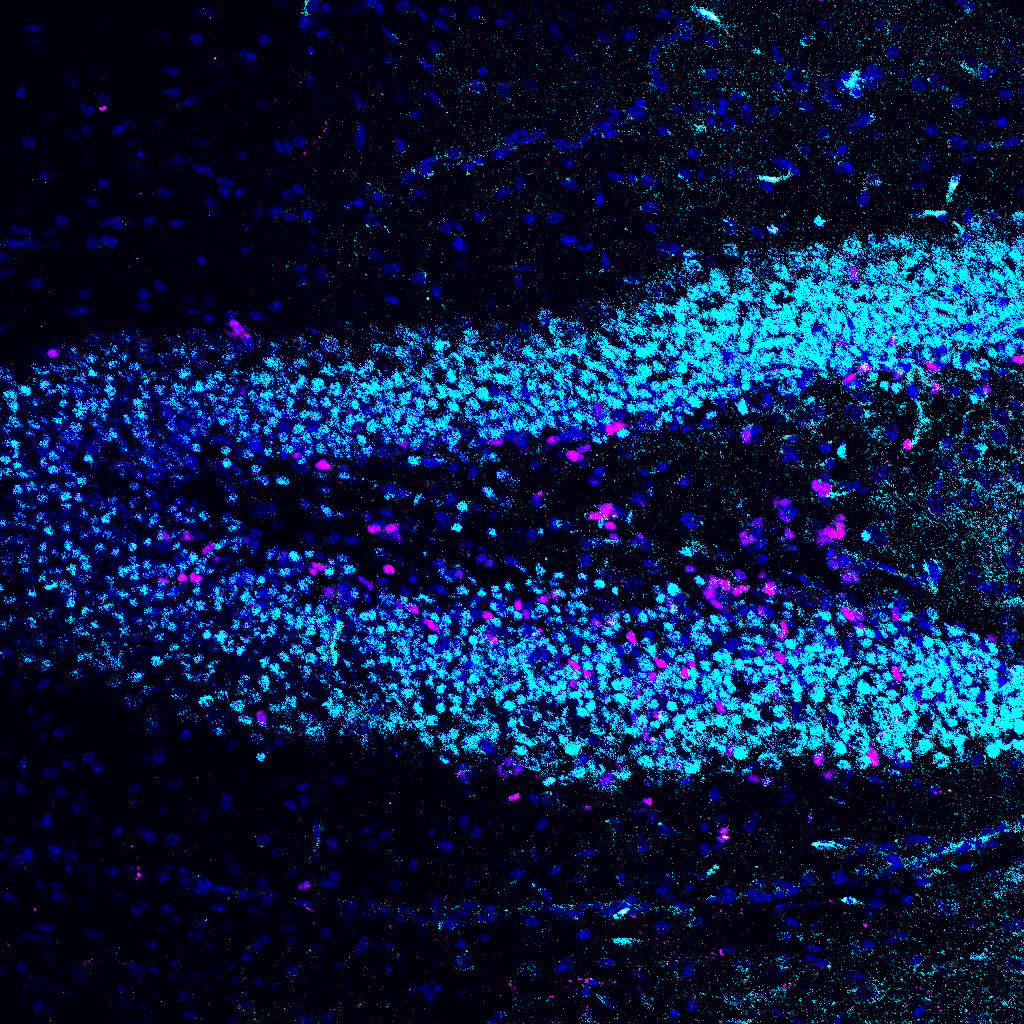

Supplement: Supplementary file 5 — Source data Fig. 3 [file 44318_2026_752_MOESM5_ESM.zip › Source Data for Figure 4/4H/WT.tif]

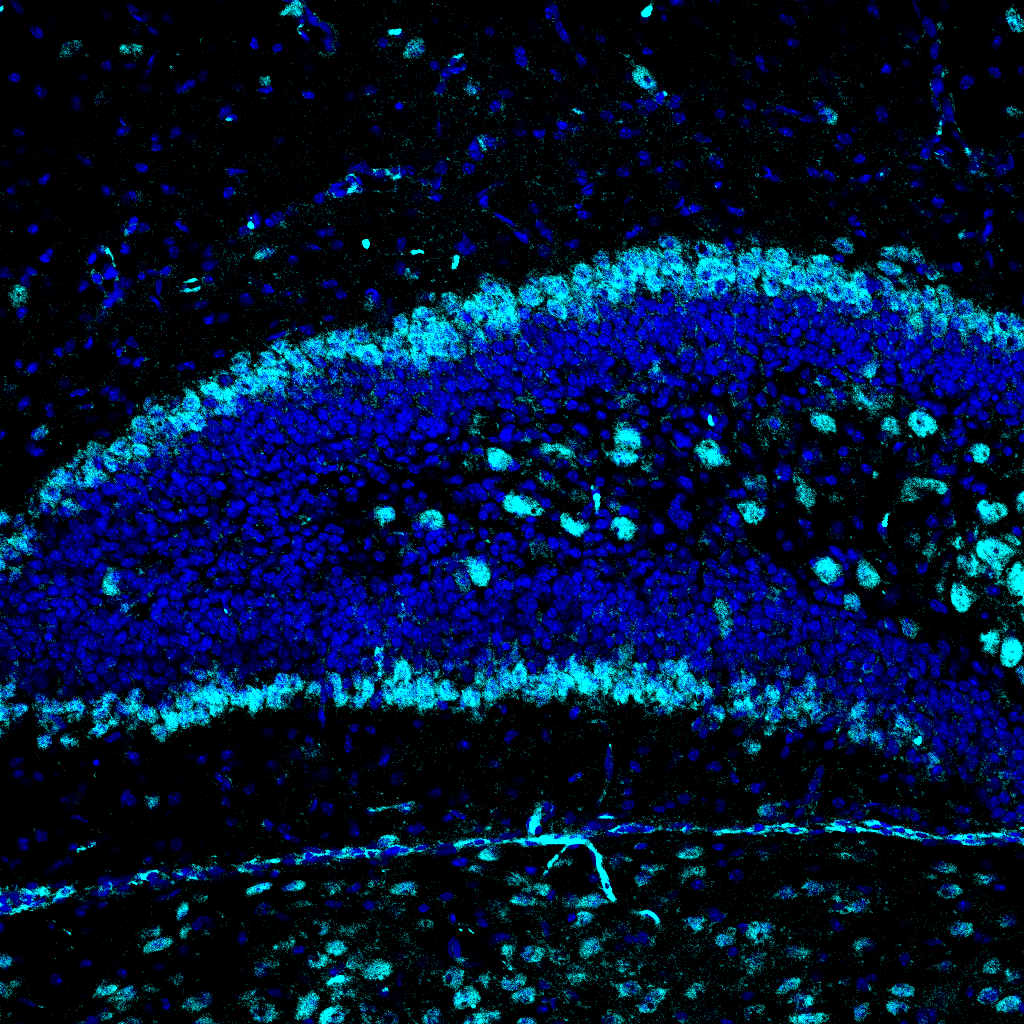

Supplement: Supplementary file 5 — Source data Fig. 3 [file 44318_2026_752_MOESM5_ESM.zip › Source Data for Figure 4/4I/Larp7ff;Emx1-Cre.tif]

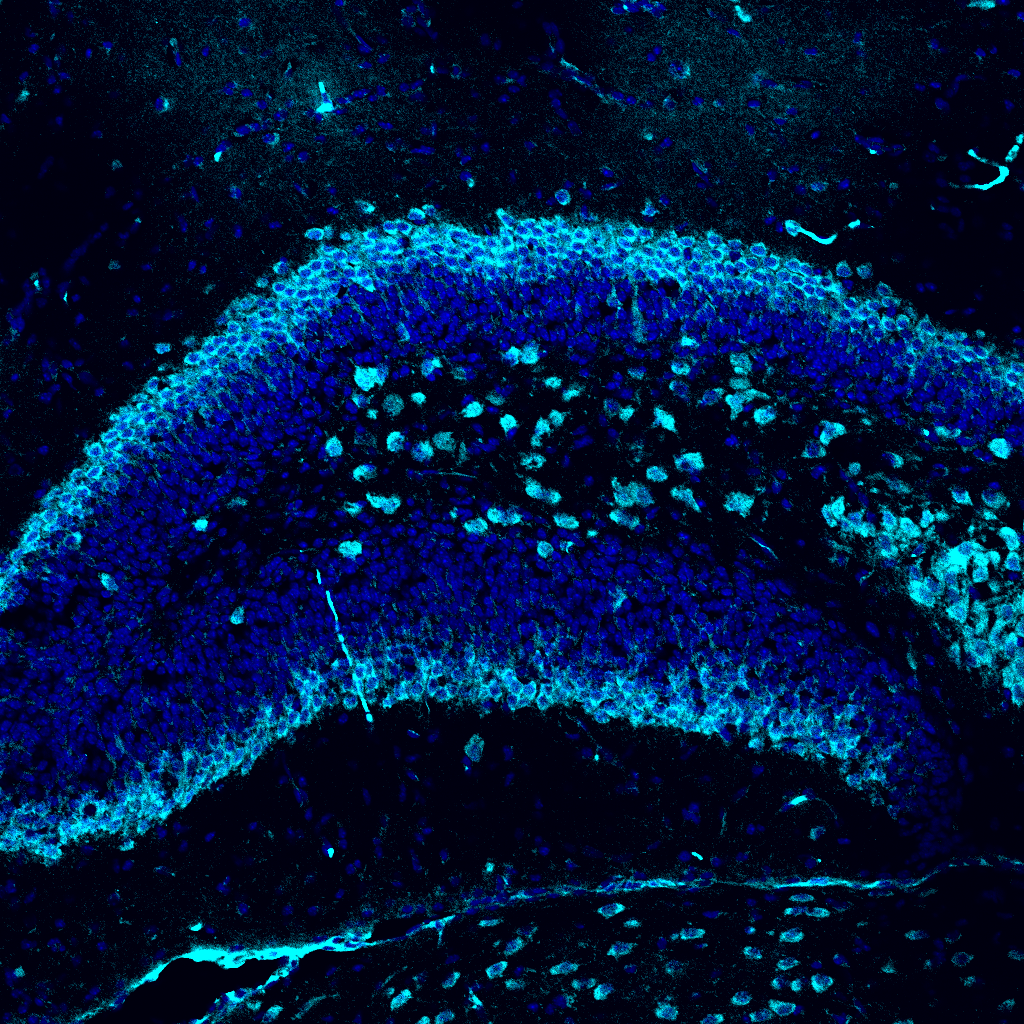

Supplement: Supplementary file 5 — Source data Fig. 3 [file 44318_2026_752_MOESM5_ESM.zip › Source Data for Figure 4/4I/WT.tif]

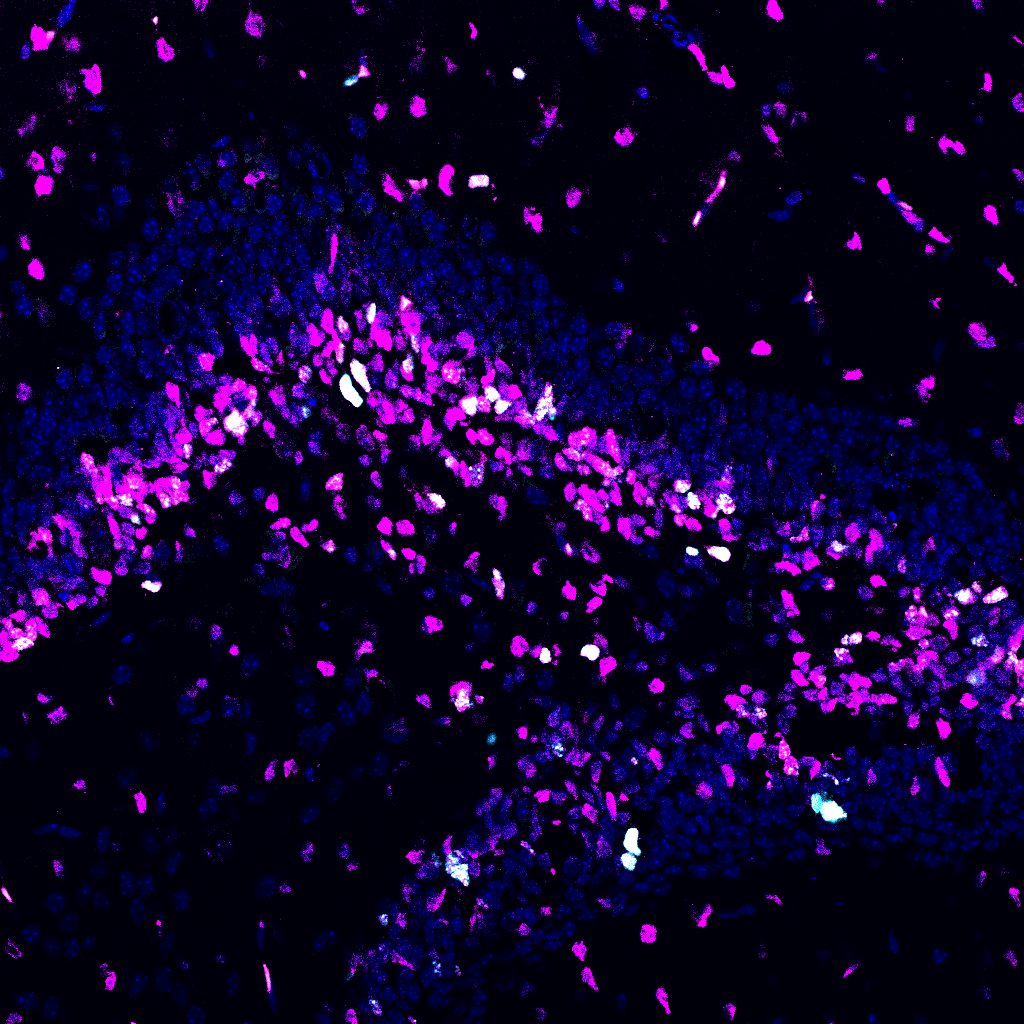

Supplement: Supplementary file 5 — Source data Fig. 3 [file 44318_2026_752_MOESM5_ESM.zip › Source Data for Figure 4/4J/Larp7ff;Emx1-Cre.tif]

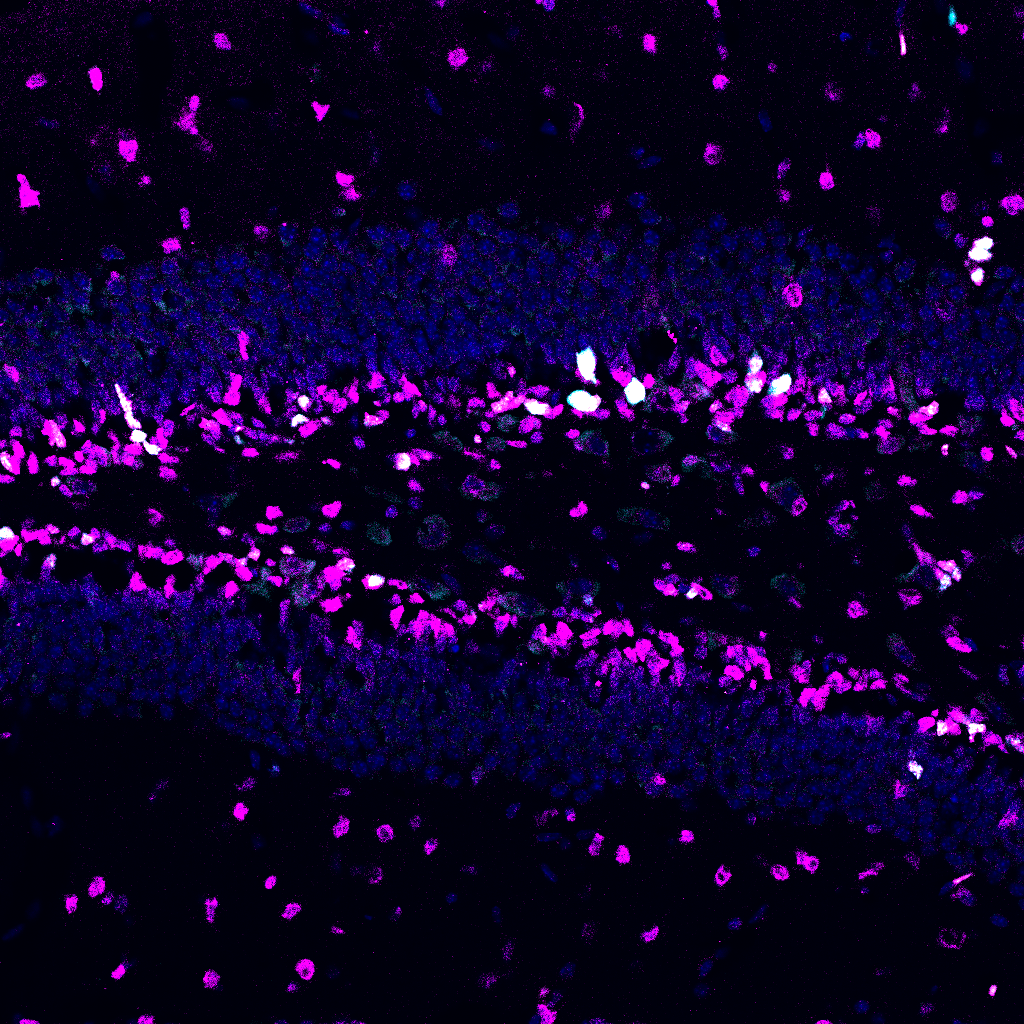

Supplement: Supplementary file 5 — Source data Fig. 3 [file 44318_2026_752_MOESM5_ESM.zip › Source Data for Figure 4/4J/WT.tif]

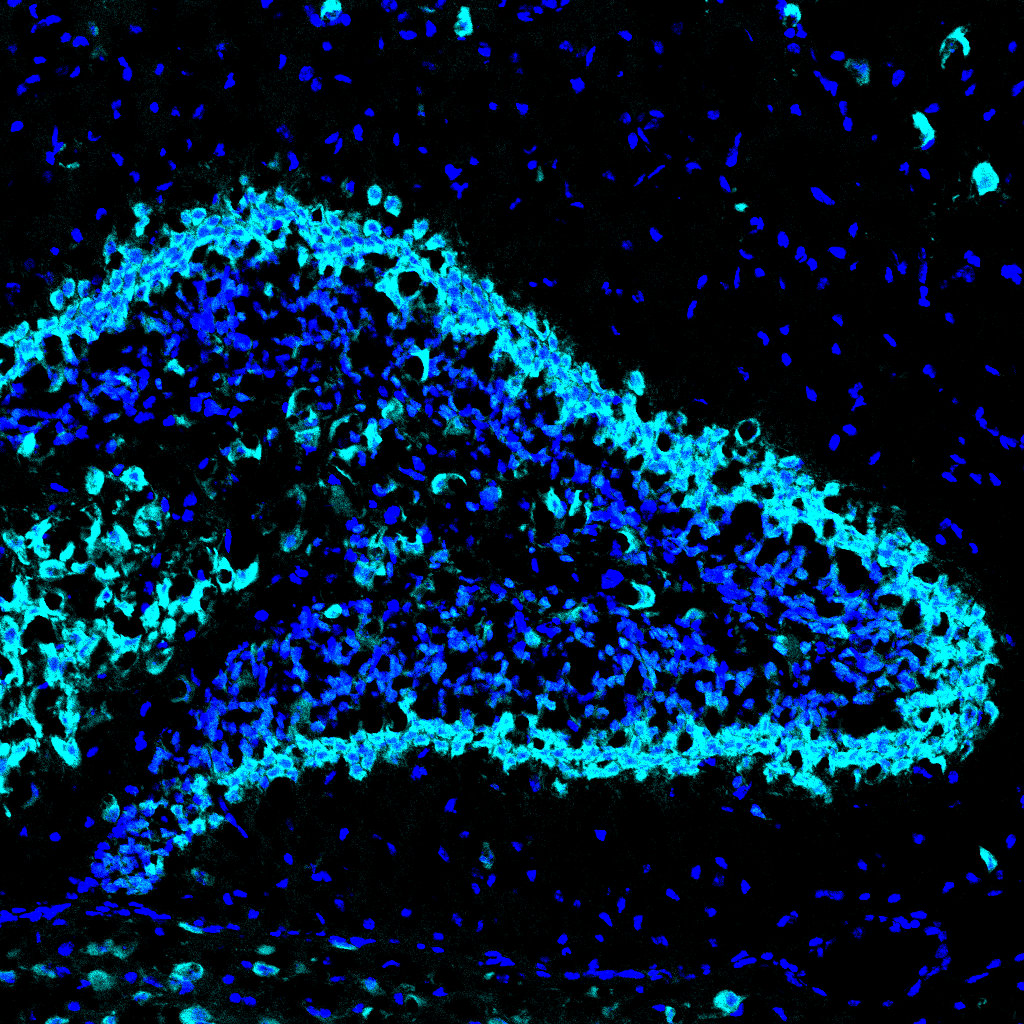

Supplement: Supplementary file 5 — Source data Fig. 3 [file 44318_2026_752_MOESM5_ESM.zip › Source Data for Figure 4/4K/Larp7ff;Emx1-Cre.tif]

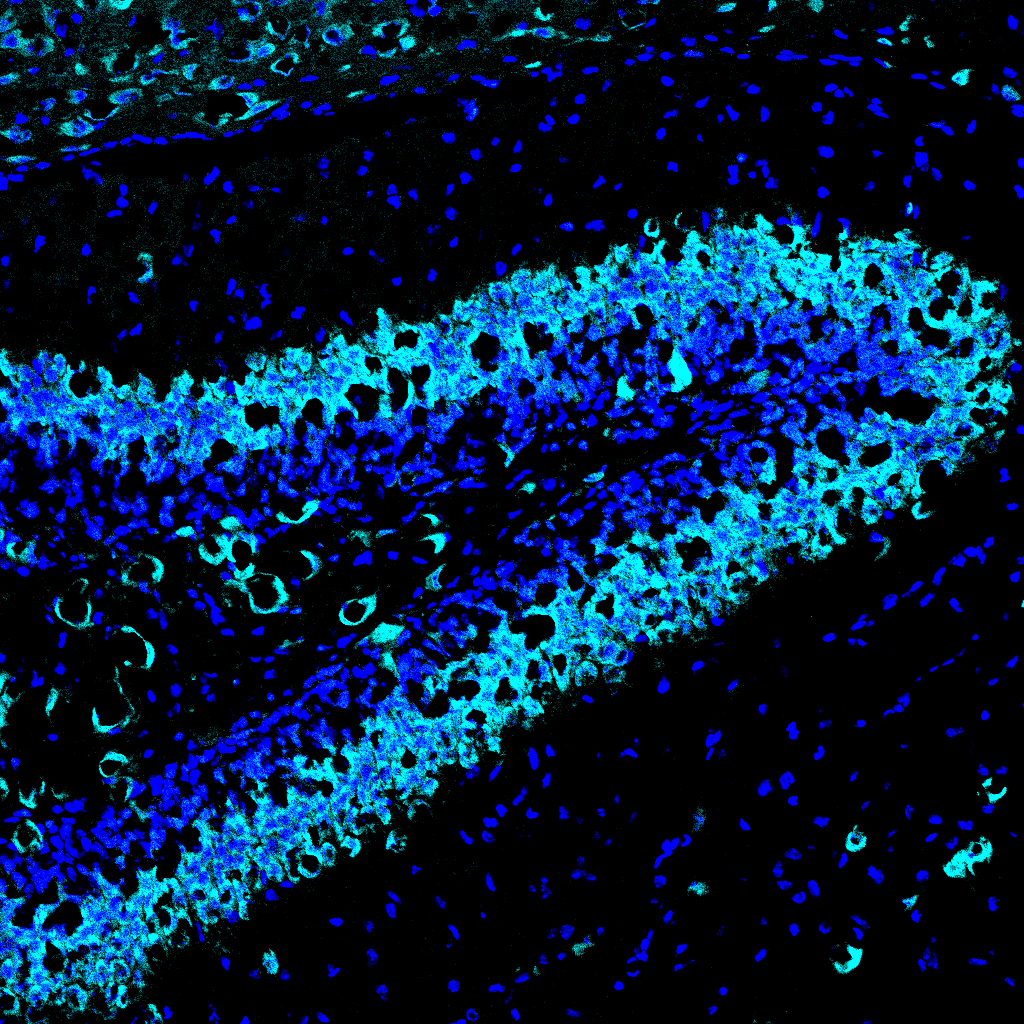

Supplement: Supplementary file 5 — Source data Fig. 3 [file 44318_2026_752_MOESM5_ESM.zip › Source Data for Figure 4/4K/WT.tif]

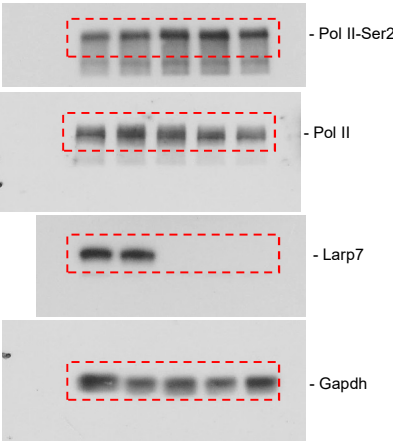

Supplement: Supplementary file 6 — Source data Fig. 6 [file 44318_2026_752_MOESM6_ESM.zip › Source Data for Figure 6/6B/western for Figure 6B.pdf]

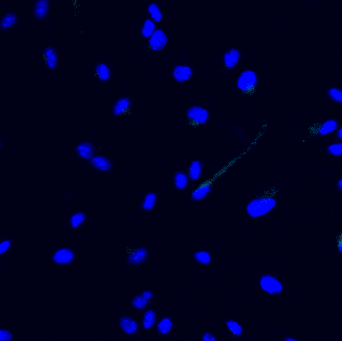

Supplement: Supplementary file 6 — Source data Fig. 6 [file 44318_2026_752_MOESM6_ESM.zip › Source Data for Figure 6/6G/KO -Day 0.tif]

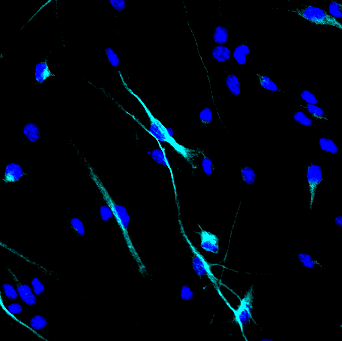

Supplement: Supplementary file 6 — Source data Fig. 6 [file 44318_2026_752_MOESM6_ESM.zip › Source Data for Figure 6/6G/KO -Day 2.tif]

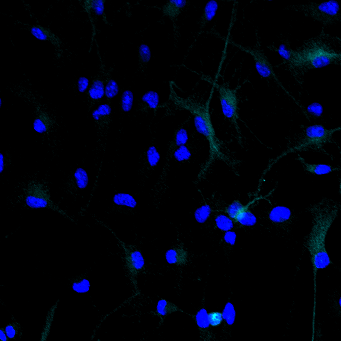

Supplement: Supplementary file 6 — Source data Fig. 6 [file 44318_2026_752_MOESM6_ESM.zip › Source Data for Figure 6/6G/WT-Day 0.tif]

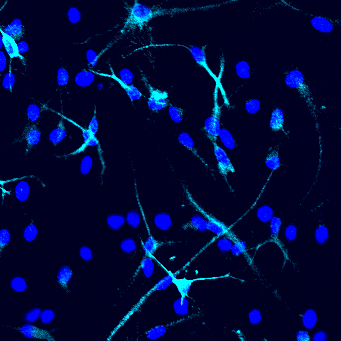

Supplement: Supplementary file 6 — Source data Fig. 6 [file 44318_2026_752_MOESM6_ESM.zip › Source Data for Figure 6/6G/WT-Day 2.tif]

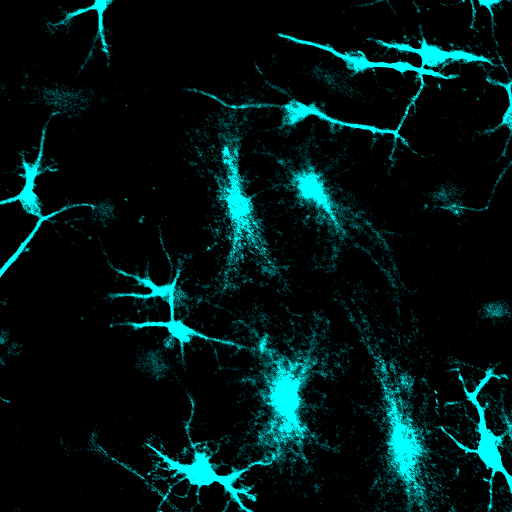

Supplement: Supplementary file 6 — Source data Fig. 6 [file 44318_2026_752_MOESM6_ESM.zip › Source Data for Figure 6/6J/KO-DMSO.tif]

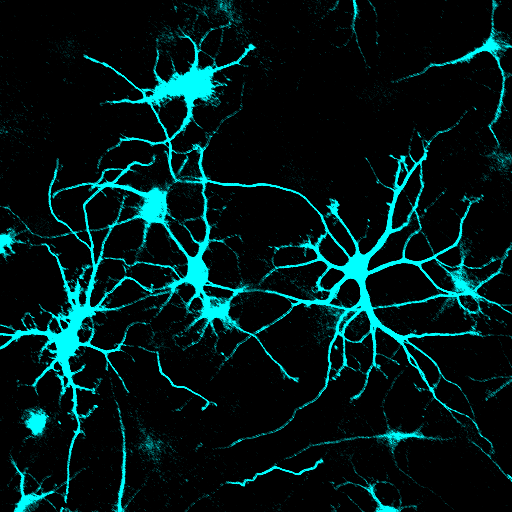

Supplement: Supplementary file 6 — Source data Fig. 6 [file 44318_2026_752_MOESM6_ESM.zip › Source Data for Figure 6/6J/KO-Flavo.tif]

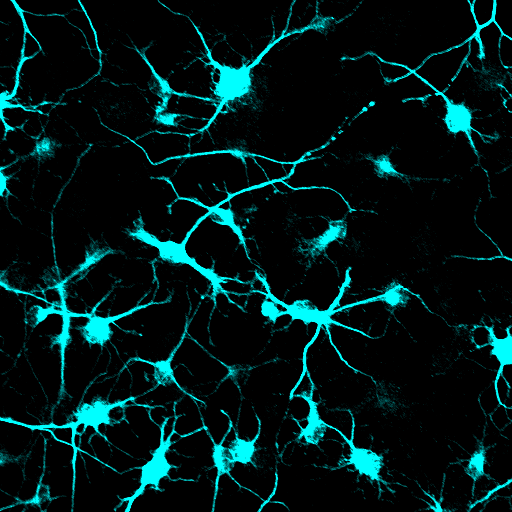

Supplement: Supplementary file 6 — Source data Fig. 6 [file 44318_2026_752_MOESM6_ESM.zip › Source Data for Figure 6/6J/WT-DMSO.tif]

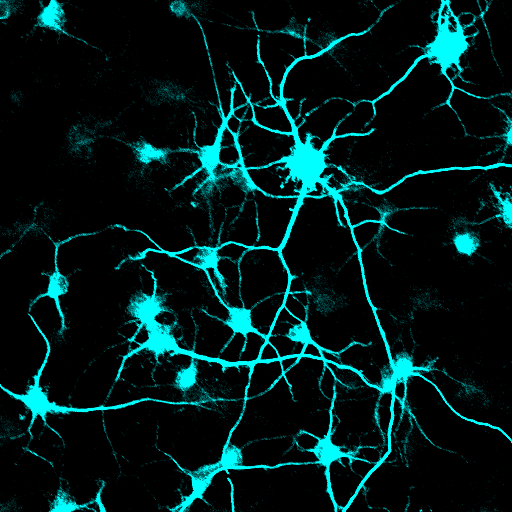

Supplement: Supplementary file 6 — Source data Fig. 6 [file 44318_2026_752_MOESM6_ESM.zip › Source Data for Figure 6/6J/WT-Flavo.tif]

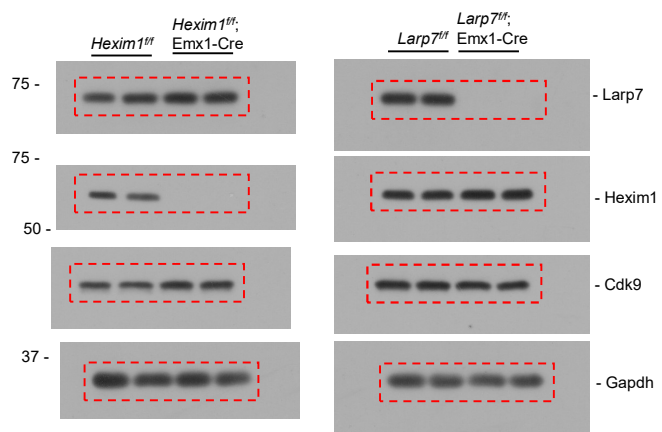

Supplement: Supplementary file 7 — Source data Fig. 7 [file 44318_2026_752_MOESM7_ESM.zip › Source Data for Figure 7/7O/western for Figure 7O.pdf]

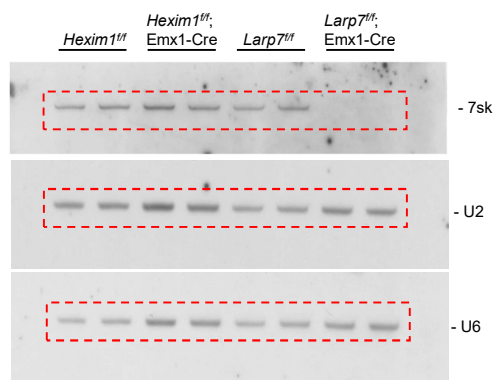

Supplement: Supplementary file 7 — Source data Fig. 7 [file 44318_2026_752_MOESM7_ESM.zip › Source Data for Figure 7/7P/northern for Figure 7P.pdf]

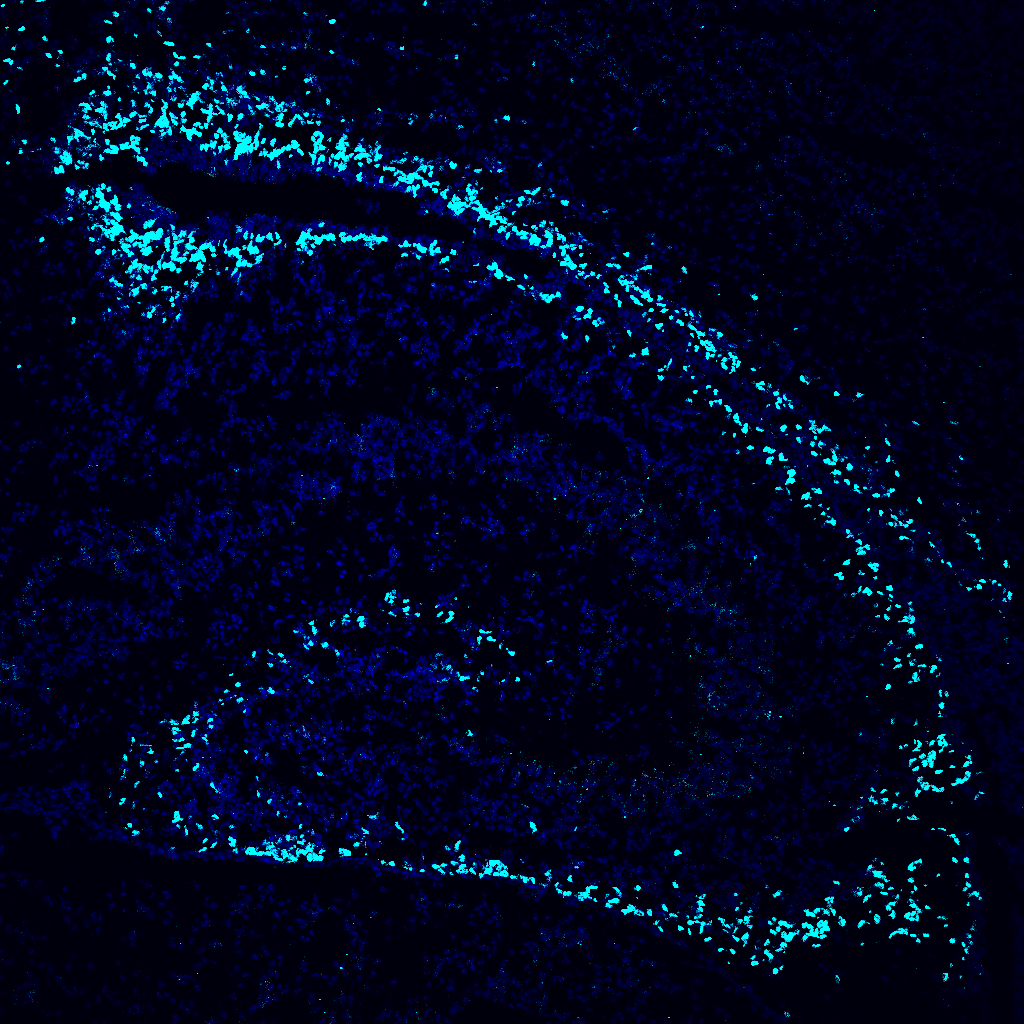

Supplement: Supplementary file 8 — Appendix Figure Source Data [file 44318_2026_752_MOESM8_ESM.zip › EMBOJ-2025-122043-Appendix source data/Appendix Figure S1/Larp7ff;nestin-Cre.tif]

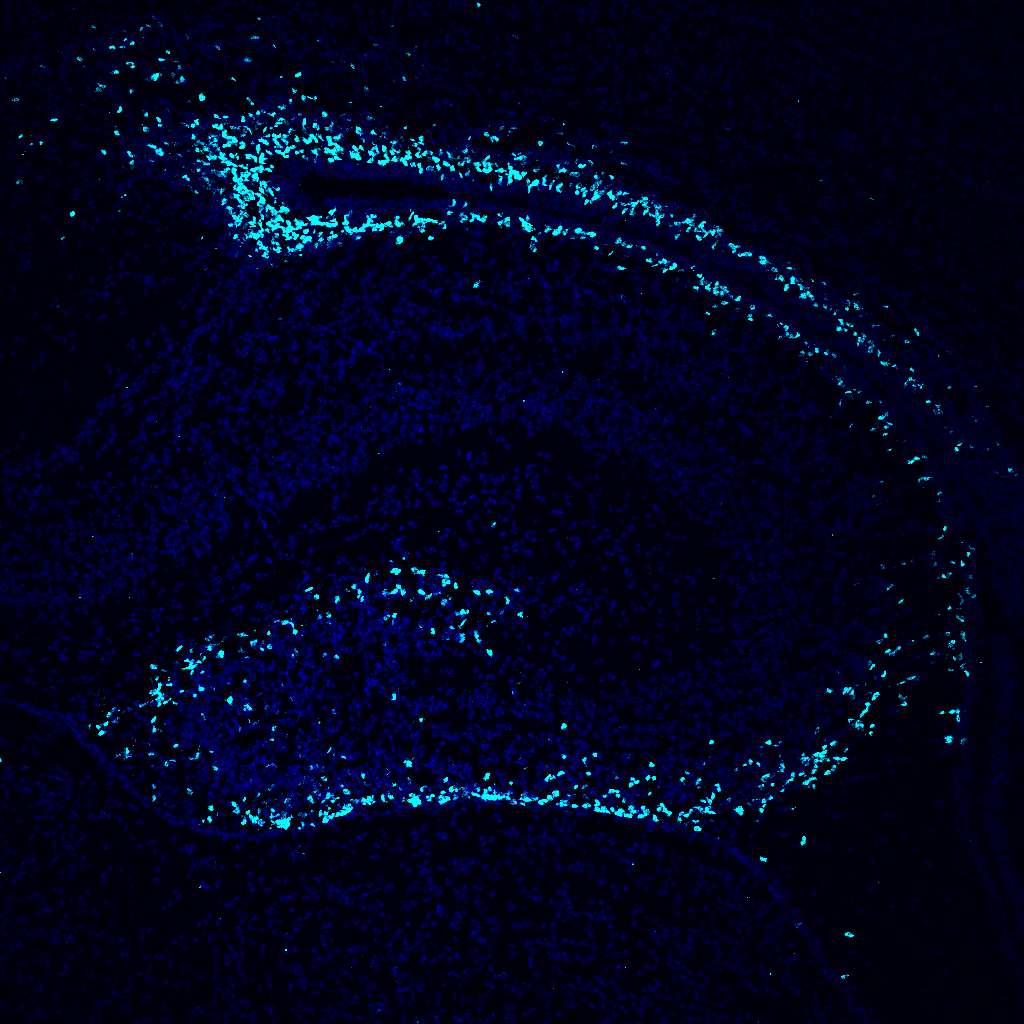

Supplement: Supplementary file 8 — Appendix Figure Source Data [file 44318_2026_752_MOESM8_ESM.zip › EMBOJ-2025-122043-Appendix source data/Appendix Figure S1/WT.tif]

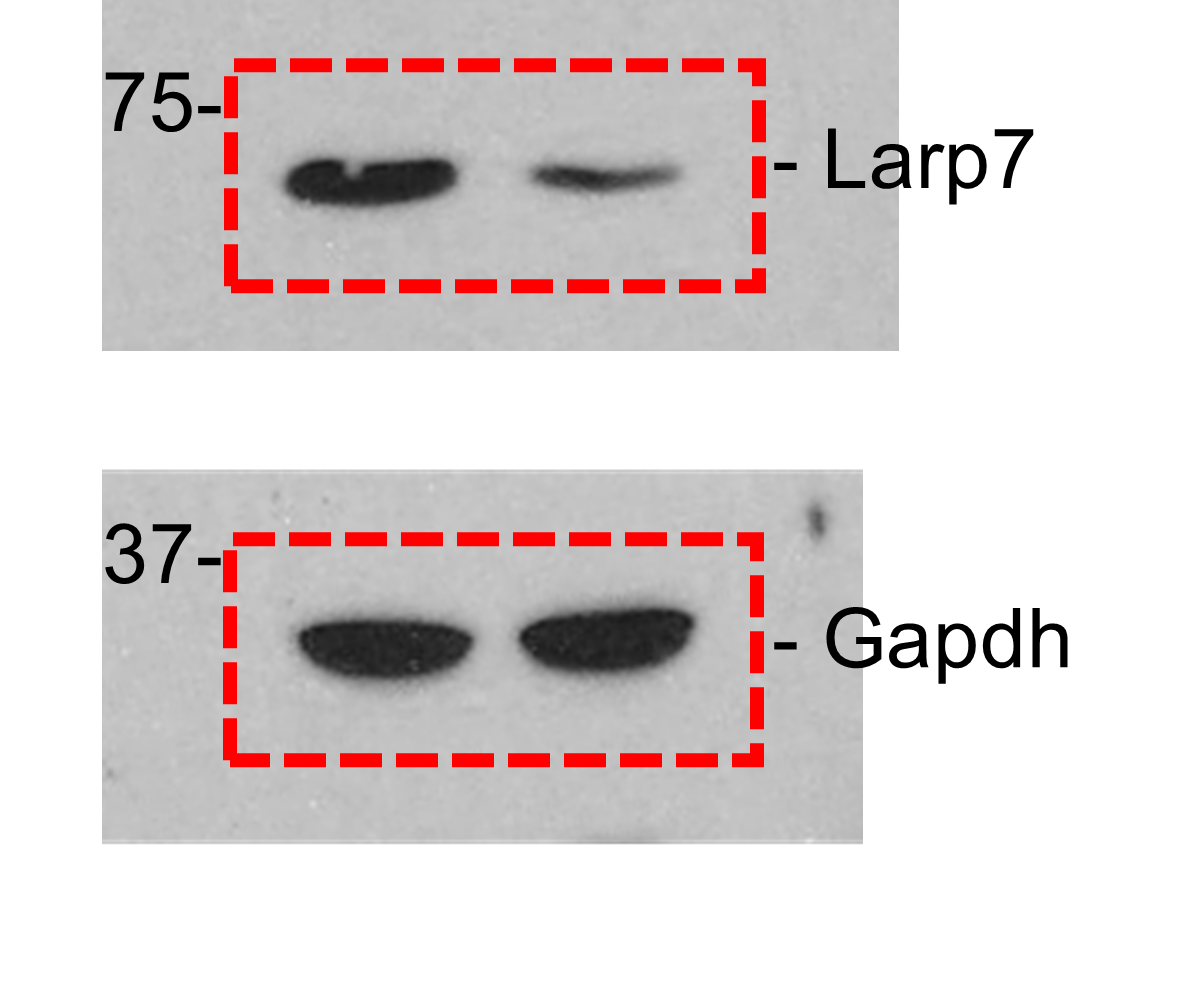

Supplement: Supplementary file 8 — Appendix Figure Source Data [file 44318_2026_752_MOESM8_ESM.zip › EMBOJ-2025-122043-Appendix source data/Appendix Figure S3/S3 B.tif]

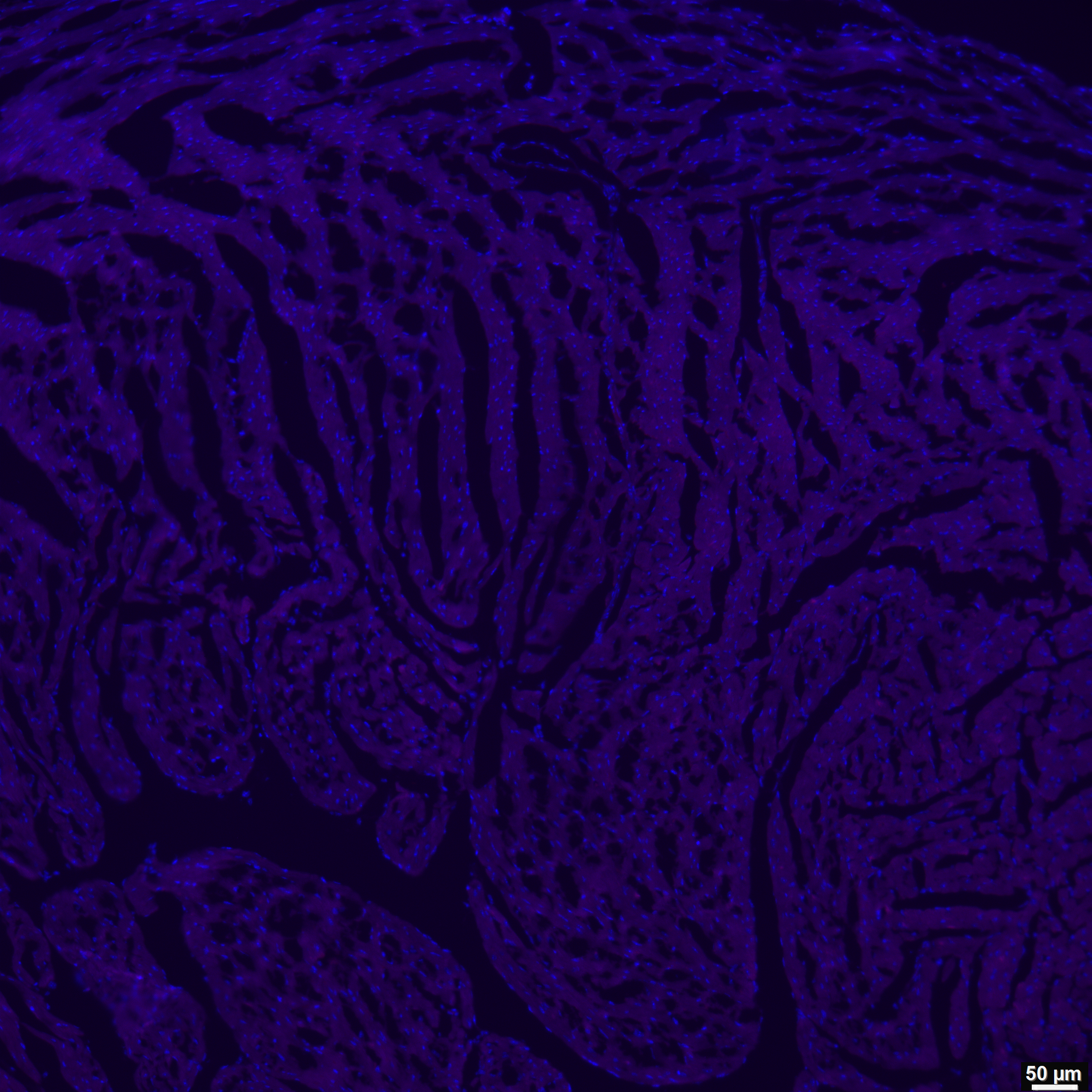

Supplement: Supplementary file 8 — Appendix Figure Source Data [file 44318_2026_752_MOESM8_ESM.zip › EMBOJ-2025-122043-Appendix source data/Appendix Figure S3/S3A/Ai14-Heart.tif]

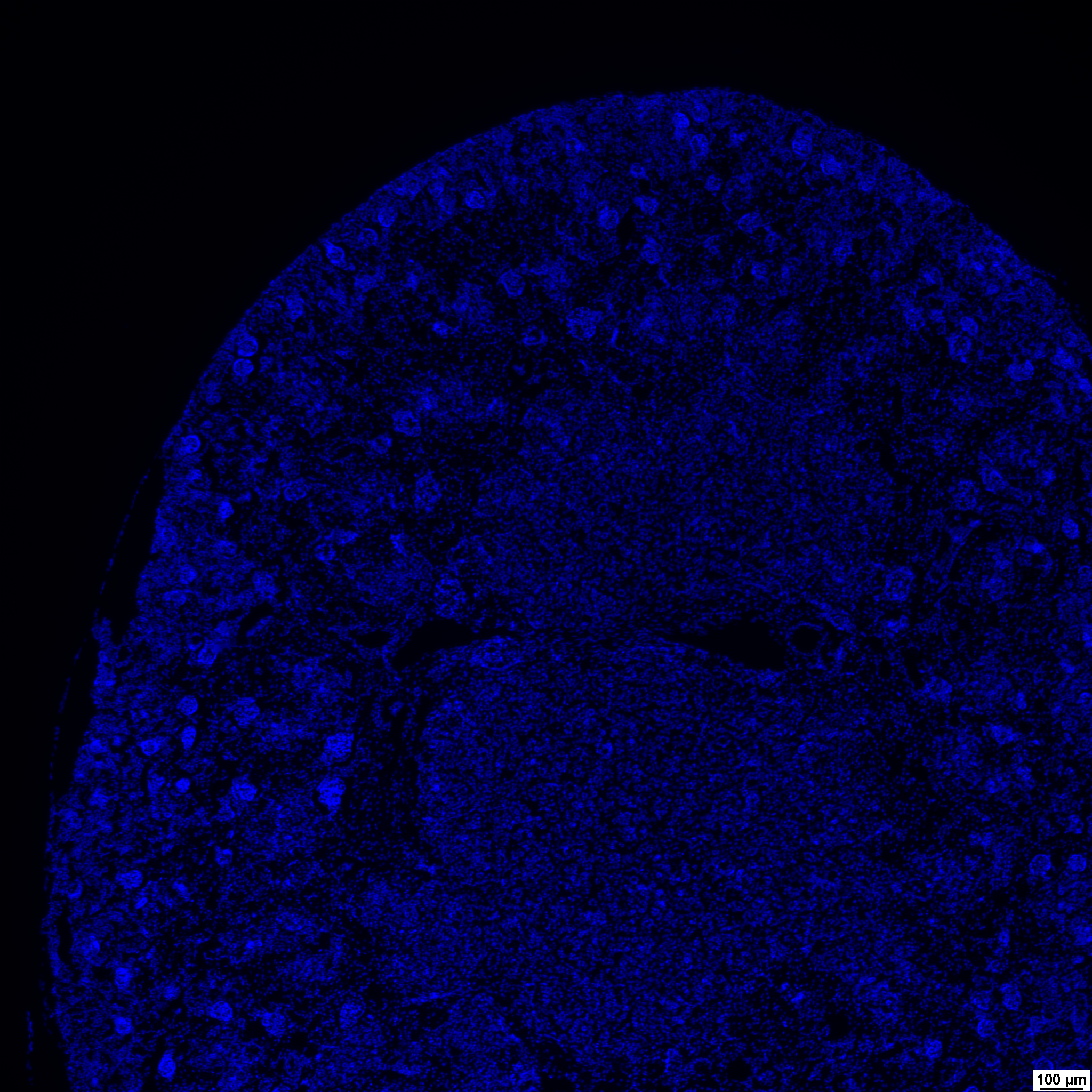

Supplement: Supplementary file 8 — Appendix Figure Source Data [file 44318_2026_752_MOESM8_ESM.zip › EMBOJ-2025-122043-Appendix source data/Appendix Figure S3/S3A/Ai14-Kidney.tif]

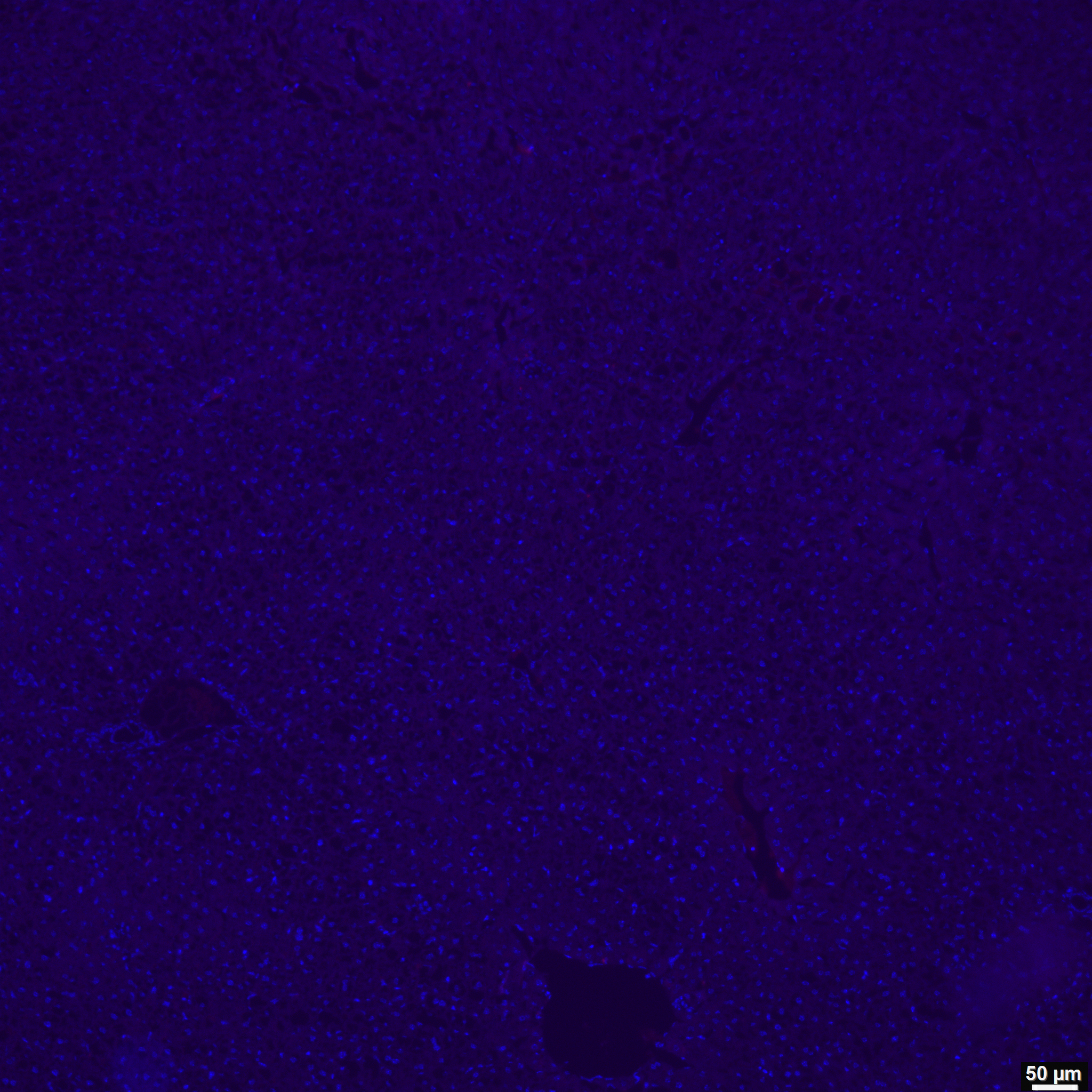

Supplement: Supplementary file 8 — Appendix Figure Source Data [file 44318_2026_752_MOESM8_ESM.zip › EMBOJ-2025-122043-Appendix source data/Appendix Figure S3/S3A/Ai14-Liver.tif]

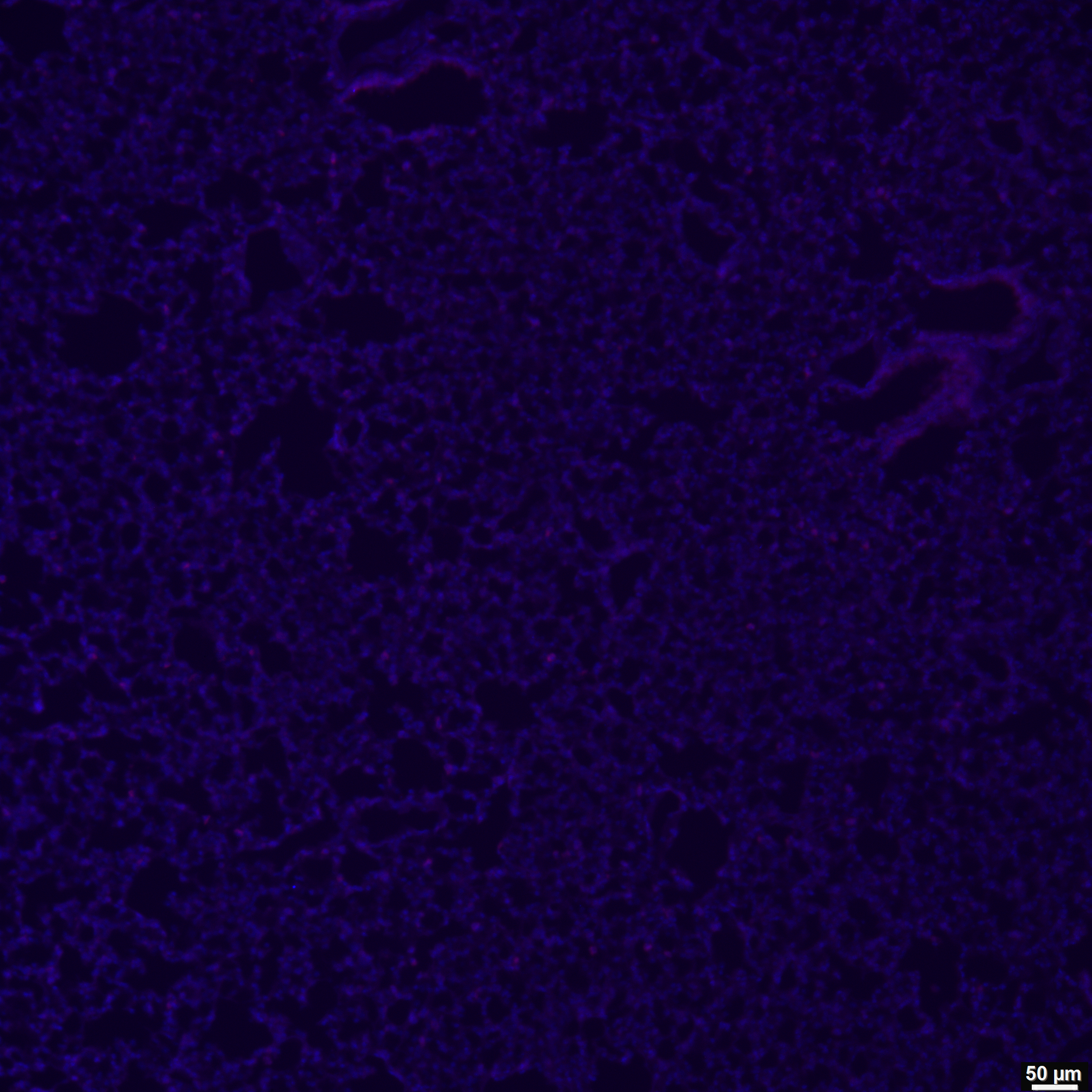

Supplement: Supplementary file 8 — Appendix Figure Source Data [file 44318_2026_752_MOESM8_ESM.zip › EMBOJ-2025-122043-Appendix source data/Appendix Figure S3/S3A/Ai14-LUNG.tif]

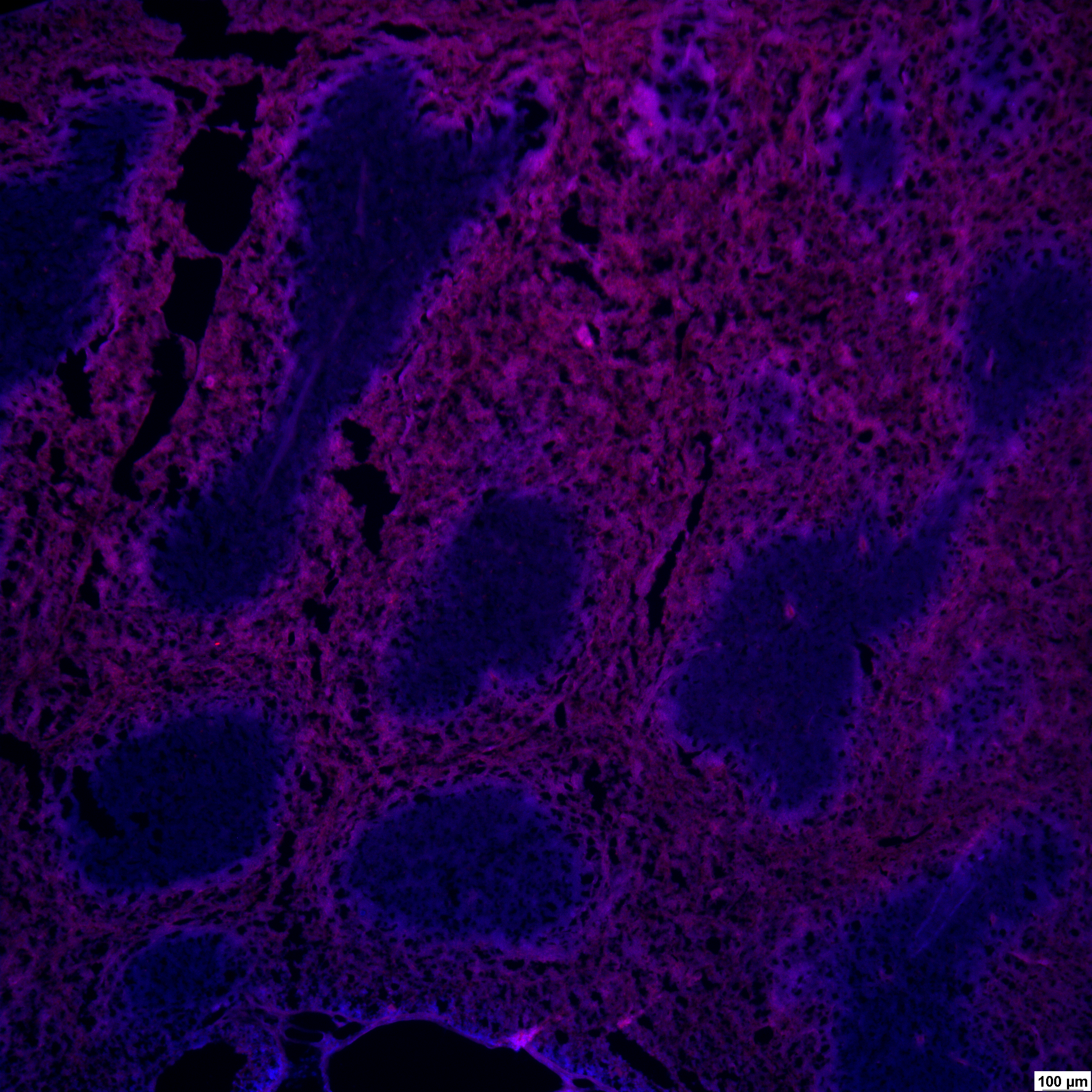

Supplement: Supplementary file 8 — Appendix Figure Source Data [file 44318_2026_752_MOESM8_ESM.zip › EMBOJ-2025-122043-Appendix source data/Appendix Figure S3/S3A/Ai14-Spleen.tif]

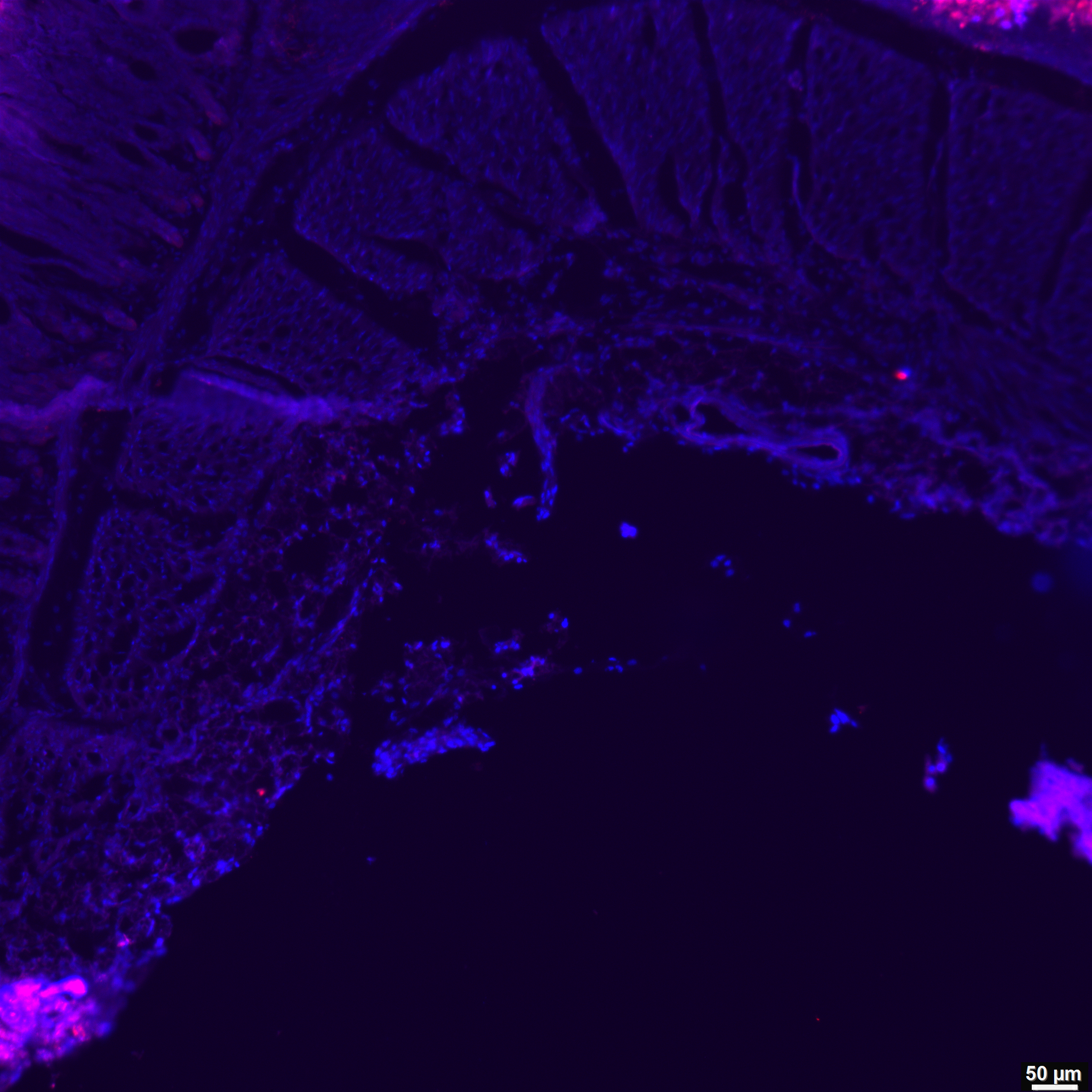

Supplement: Supplementary file 8 — Appendix Figure Source Data [file 44318_2026_752_MOESM8_ESM.zip › EMBOJ-2025-122043-Appendix source data/Appendix Figure S3/S3A/Ai14-Stomach.tif]

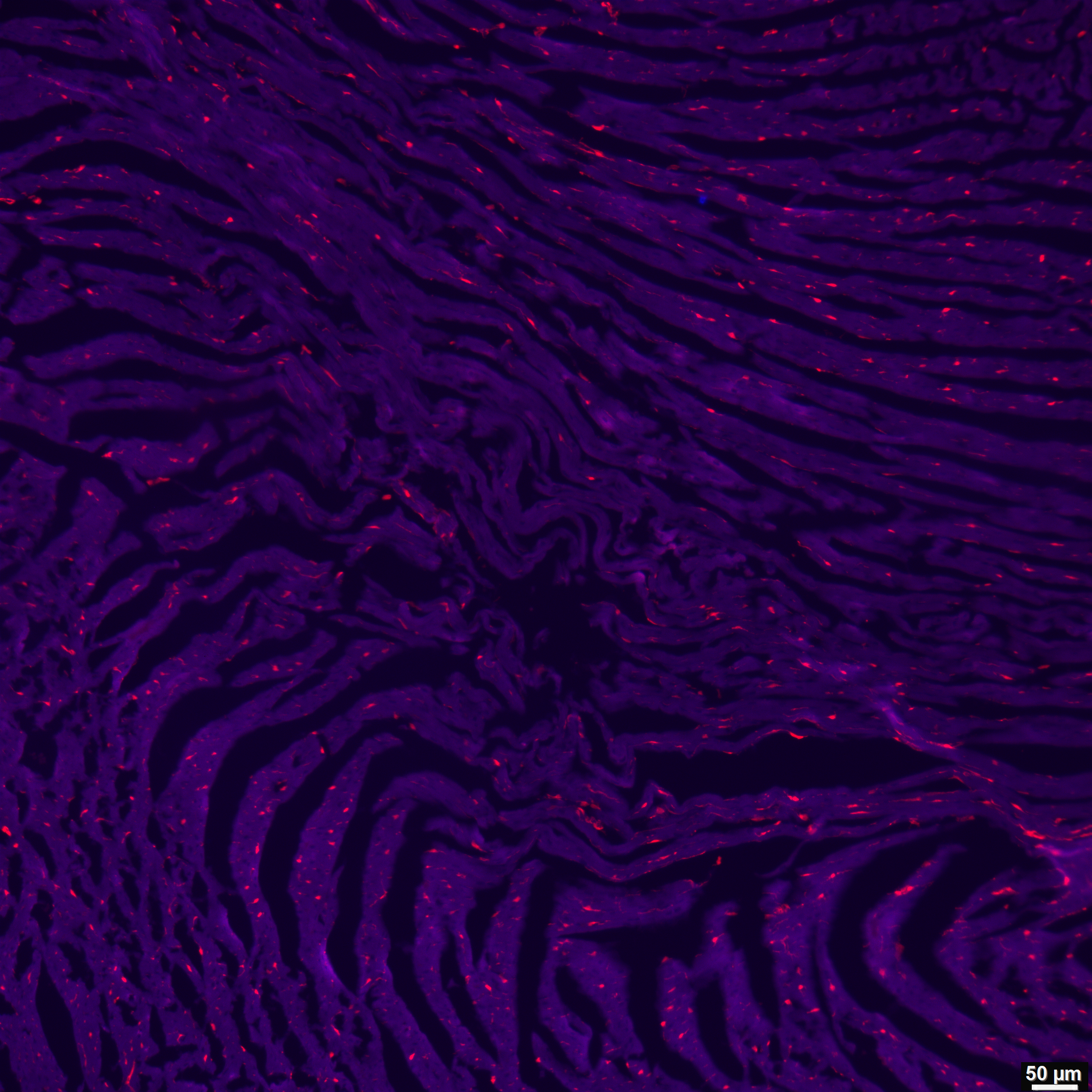

Supplement: Supplementary file 8 — Appendix Figure Source Data [file 44318_2026_752_MOESM8_ESM.zip › EMBOJ-2025-122043-Appendix source data/Appendix Figure S3/S3A/Ai14;nestin-Cre-Heart.tif]

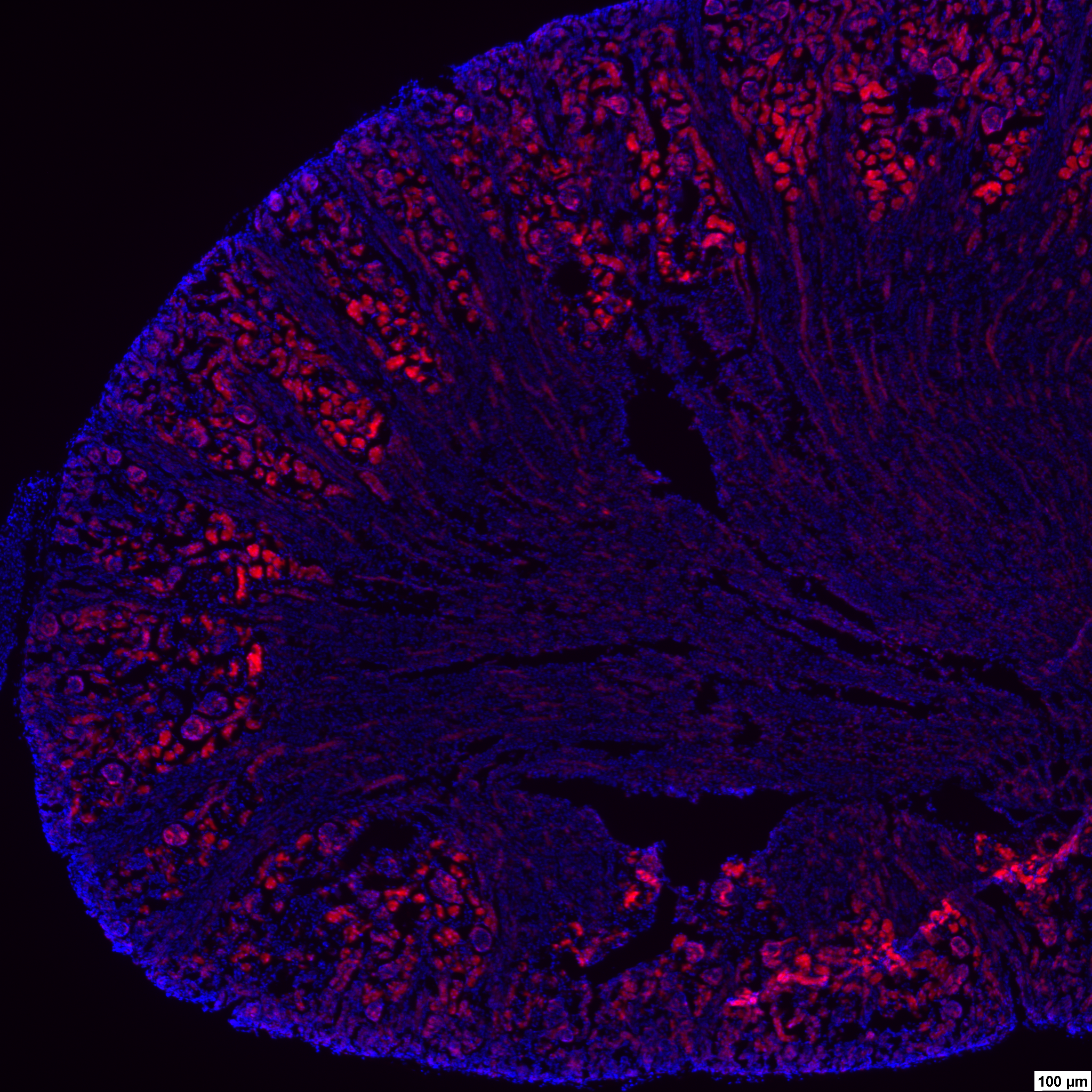

Supplement: Supplementary file 8 — Appendix Figure Source Data [file 44318_2026_752_MOESM8_ESM.zip › EMBOJ-2025-122043-Appendix source data/Appendix Figure S3/S3A/Ai14;nestin-Cre-Kidney.tif]

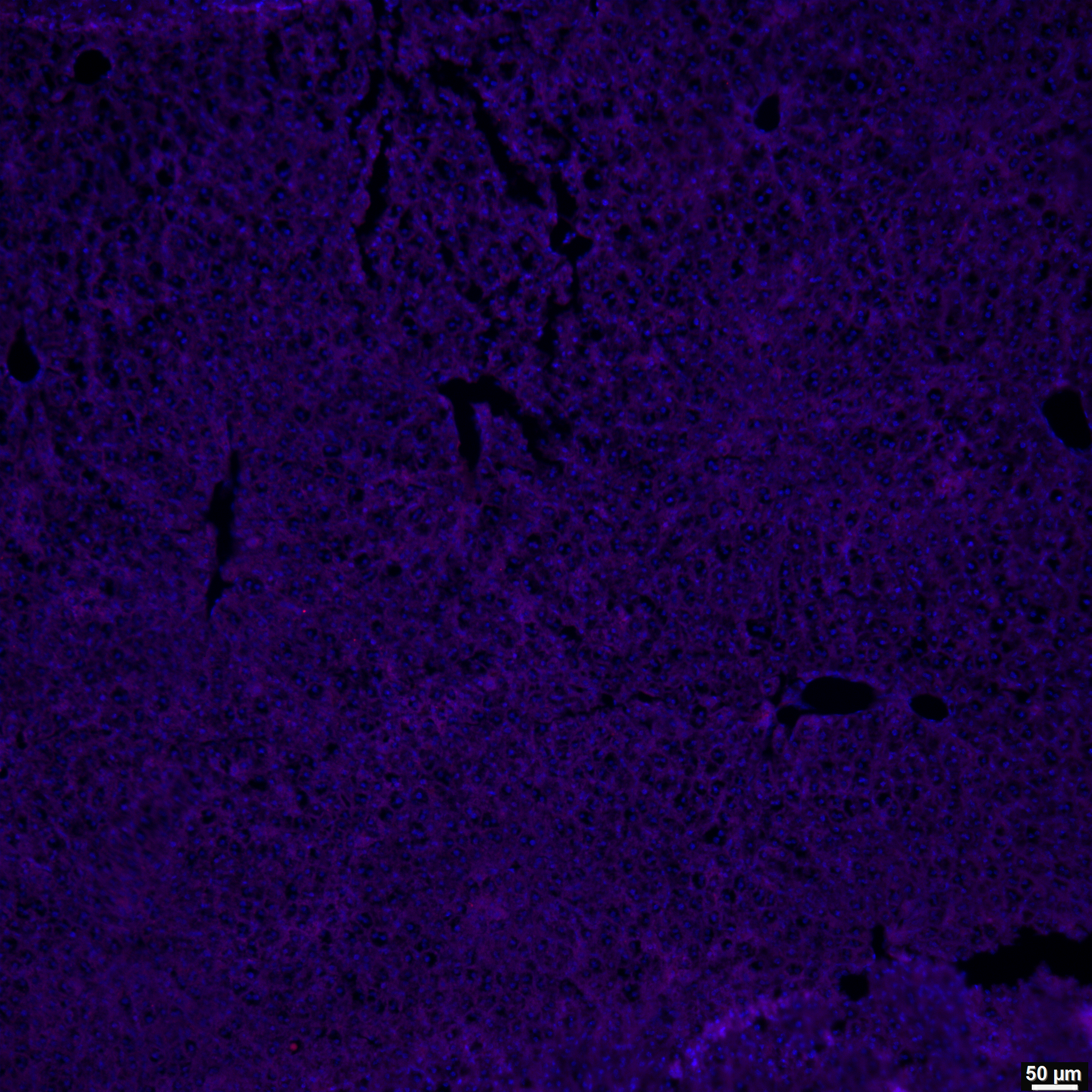

Supplement: Supplementary file 8 — Appendix Figure Source Data [file 44318_2026_752_MOESM8_ESM.zip › EMBOJ-2025-122043-Appendix source data/Appendix Figure S3/S3A/Ai14;nestin-Cre-Liver.tif]

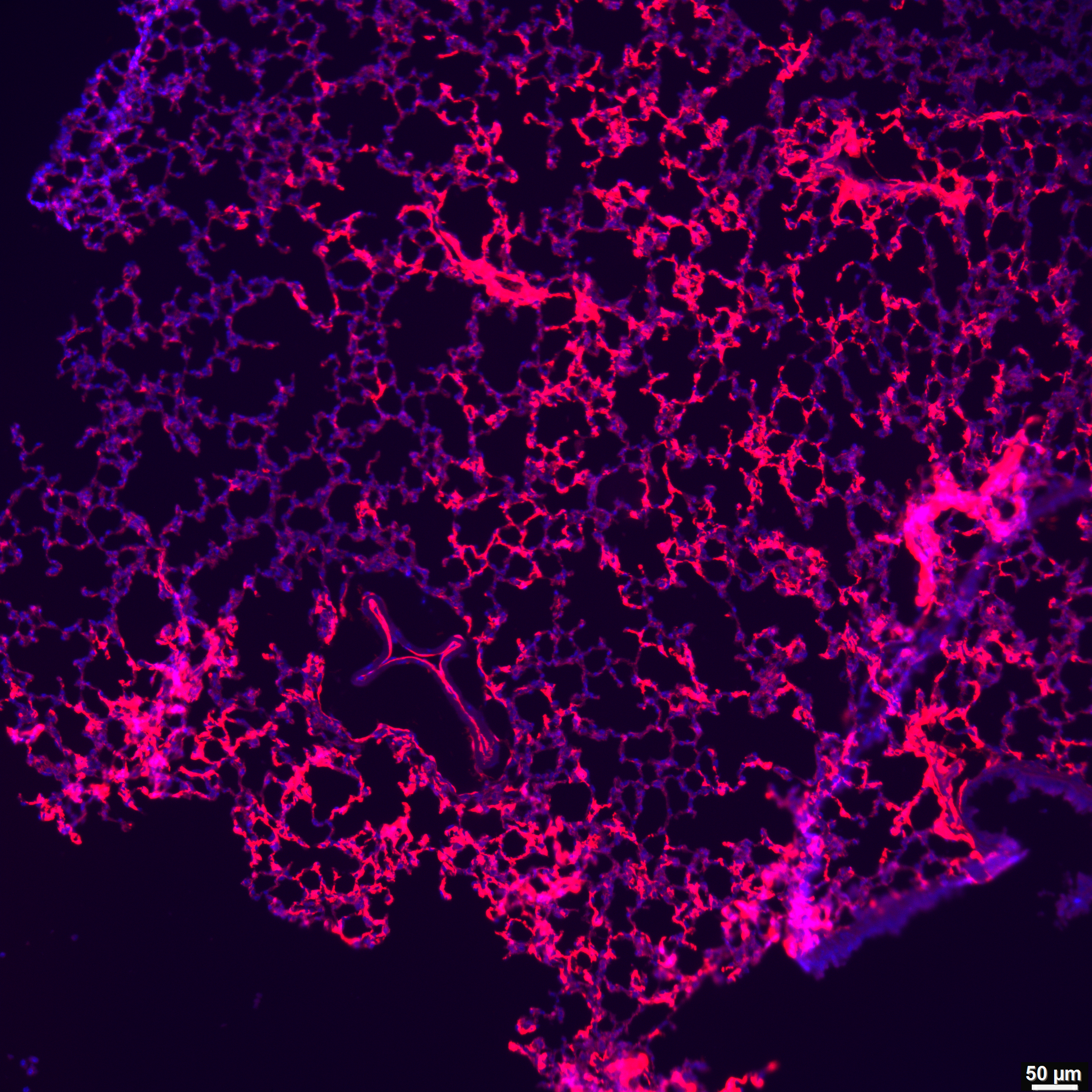

Supplement: Supplementary file 8 — Appendix Figure Source Data [file 44318_2026_752_MOESM8_ESM.zip › EMBOJ-2025-122043-Appendix source data/Appendix Figure S3/S3A/Ai14;nestin-Cre-LUNG.tif]

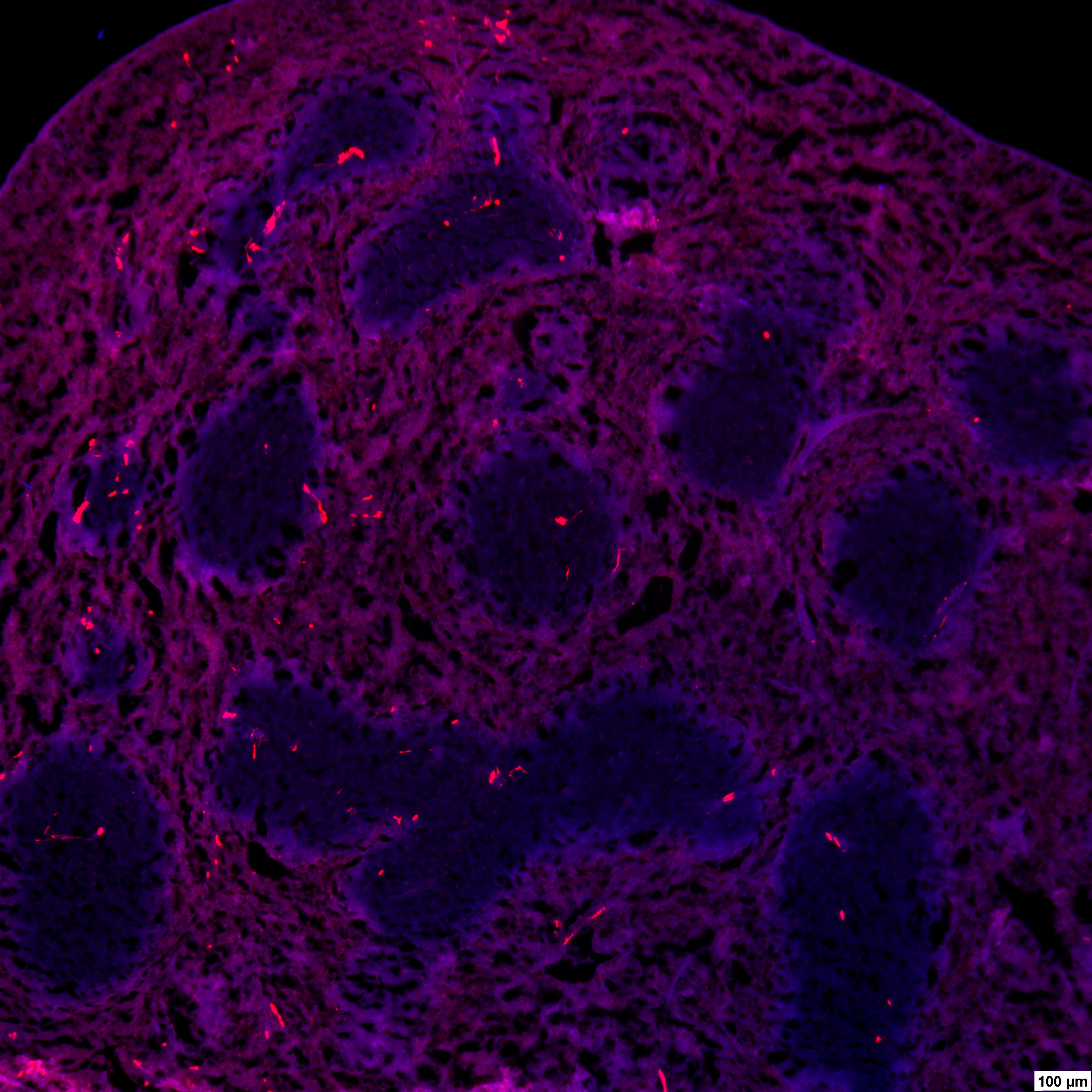

Supplement: Supplementary file 8 — Appendix Figure Source Data [file 44318_2026_752_MOESM8_ESM.zip › EMBOJ-2025-122043-Appendix source data/Appendix Figure S3/S3A/Ai14;nestin-Cre-Spleen.tif]

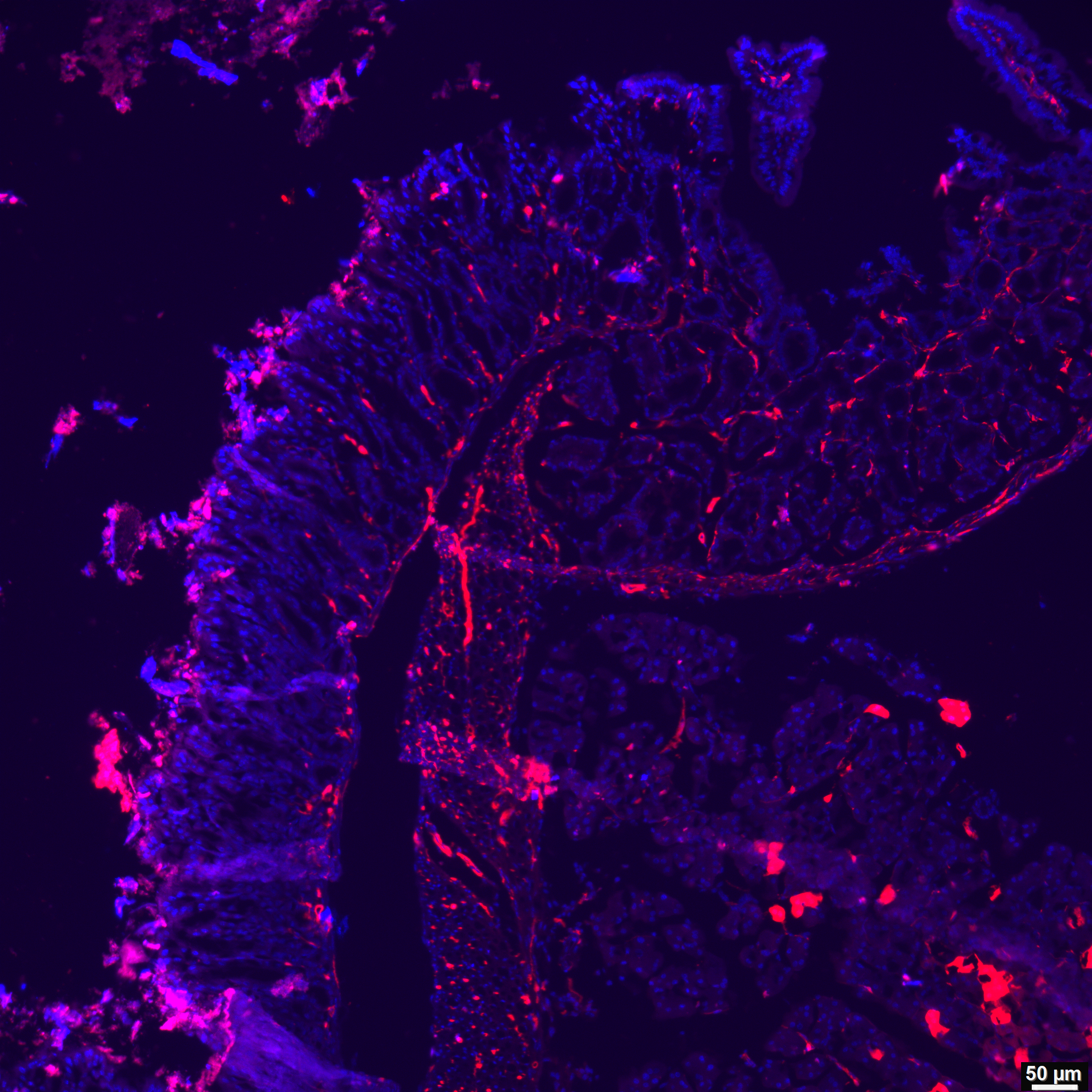

Supplement: Supplementary file 8 — Appendix Figure Source Data [file 44318_2026_752_MOESM8_ESM.zip › EMBOJ-2025-122043-Appendix source data/Appendix Figure S3/S3A/Ai14;nestin-Cre-Stomach.tif]

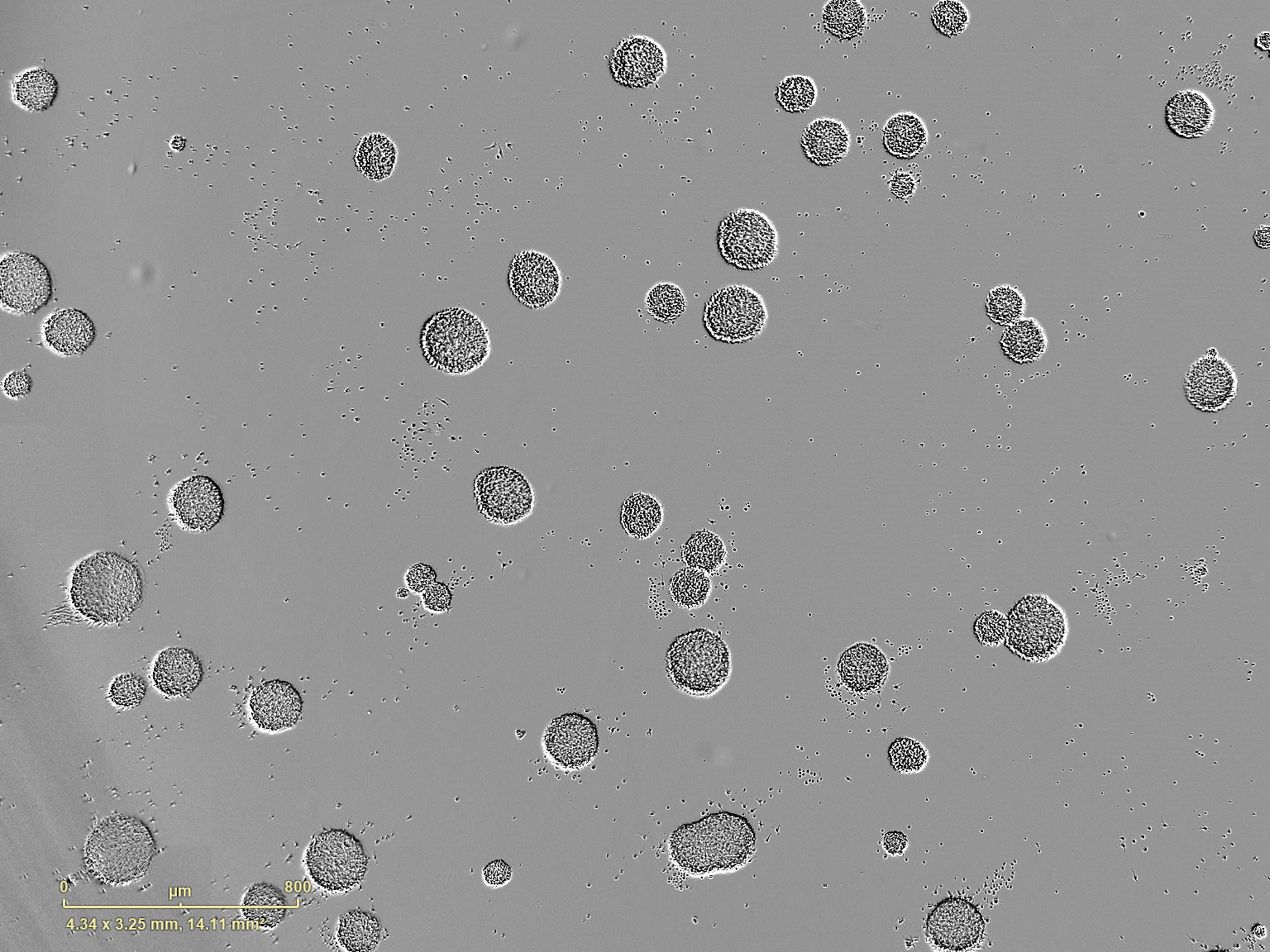

Supplement: Supplementary file 8 — Appendix Figure Source Data [file 44318_2026_752_MOESM8_ESM.zip › EMBOJ-2025-122043-Appendix source data/Appendix Figure S7/A/Larp7 ff;nestin-Cre.tif]

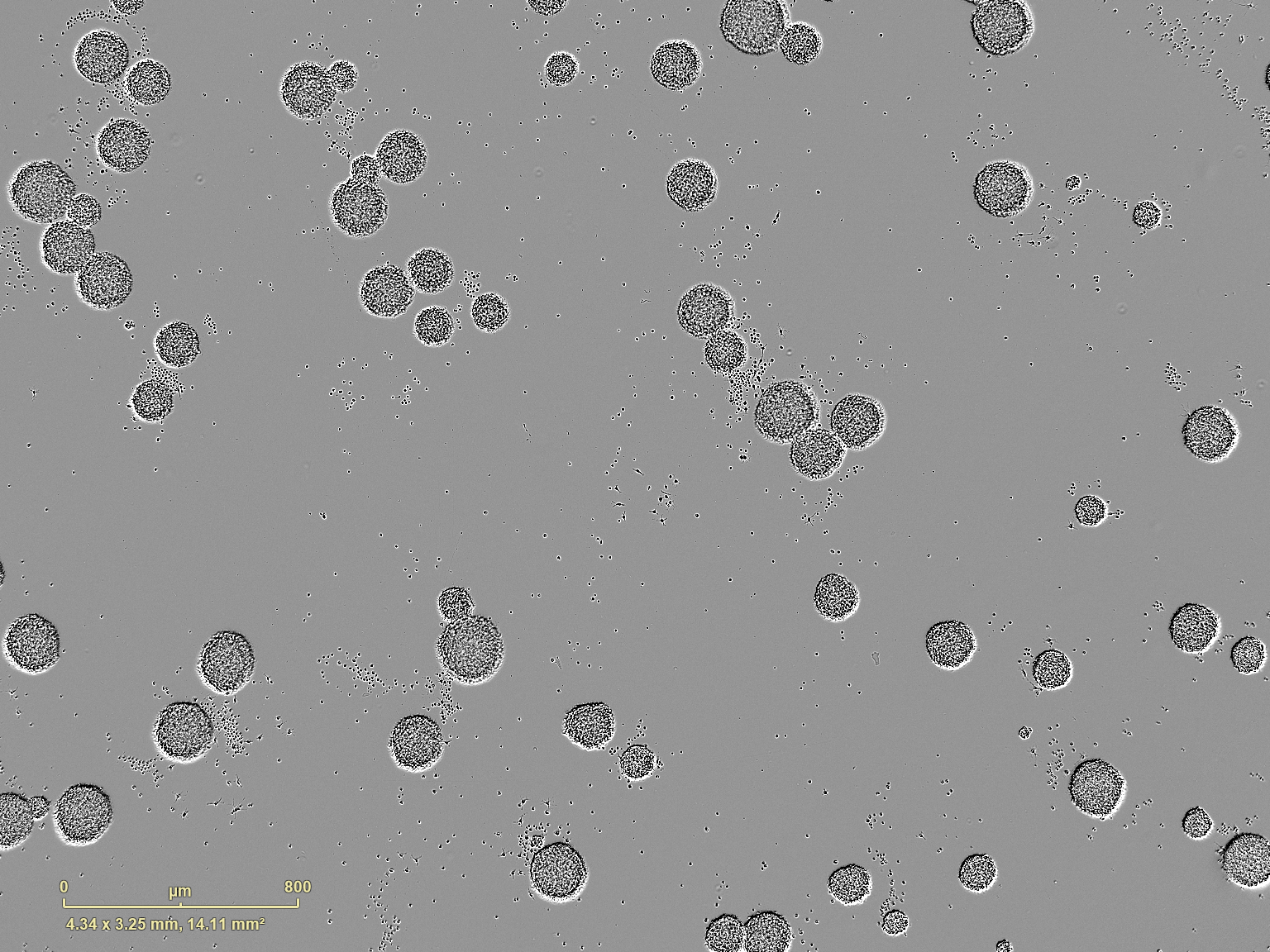

Supplement: Supplementary file 8 — Appendix Figure Source Data [file 44318_2026_752_MOESM8_ESM.zip › EMBOJ-2025-122043-Appendix source data/Appendix Figure S7/A/WT.tif]

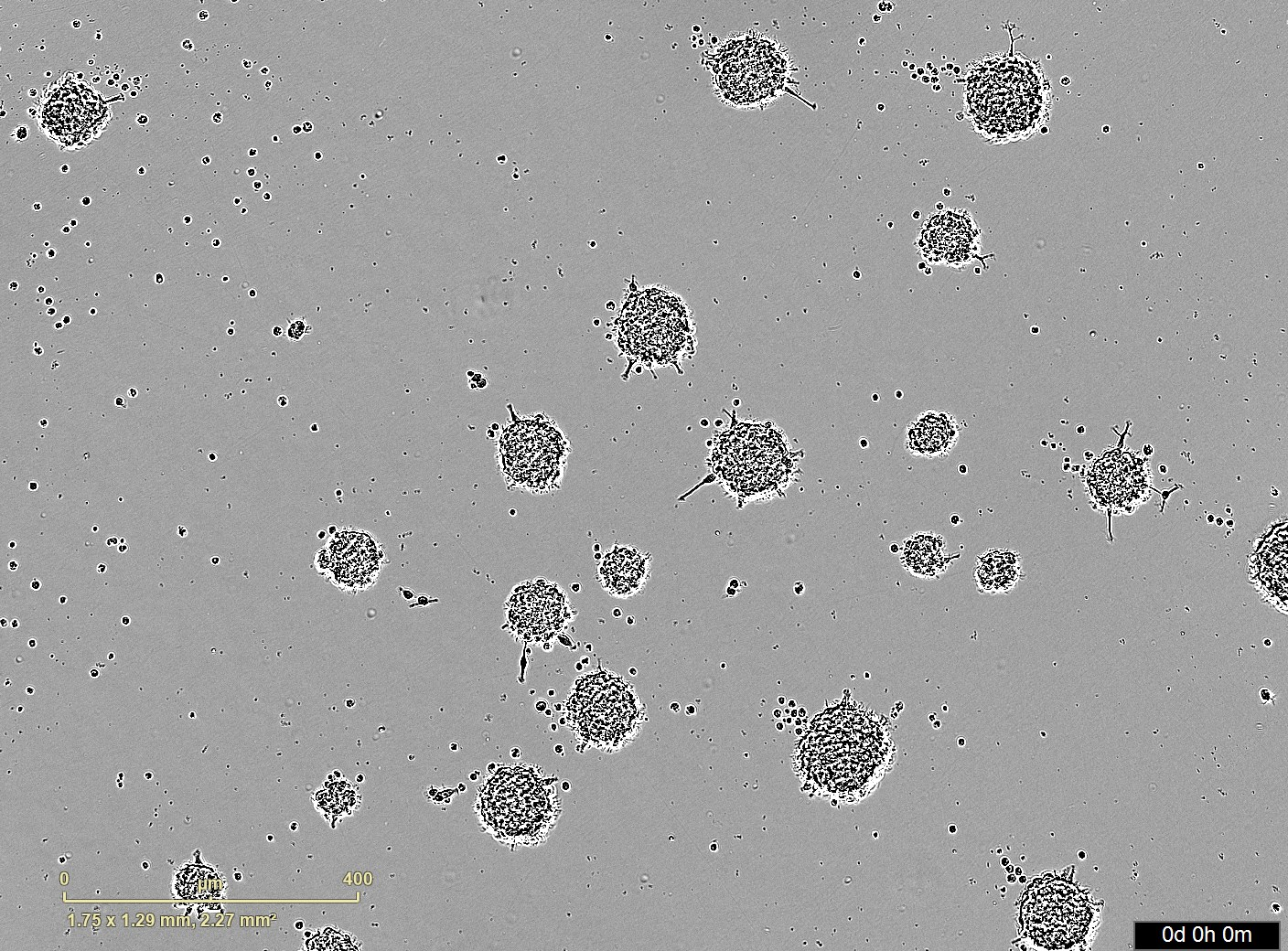

Supplement: Supplementary file 8 — Appendix Figure Source Data [file 44318_2026_752_MOESM8_ESM.zip › EMBOJ-2025-122043-Appendix source data/Appendix Figure S7/B/Larp7 ff ;nestin-Cre.tif]

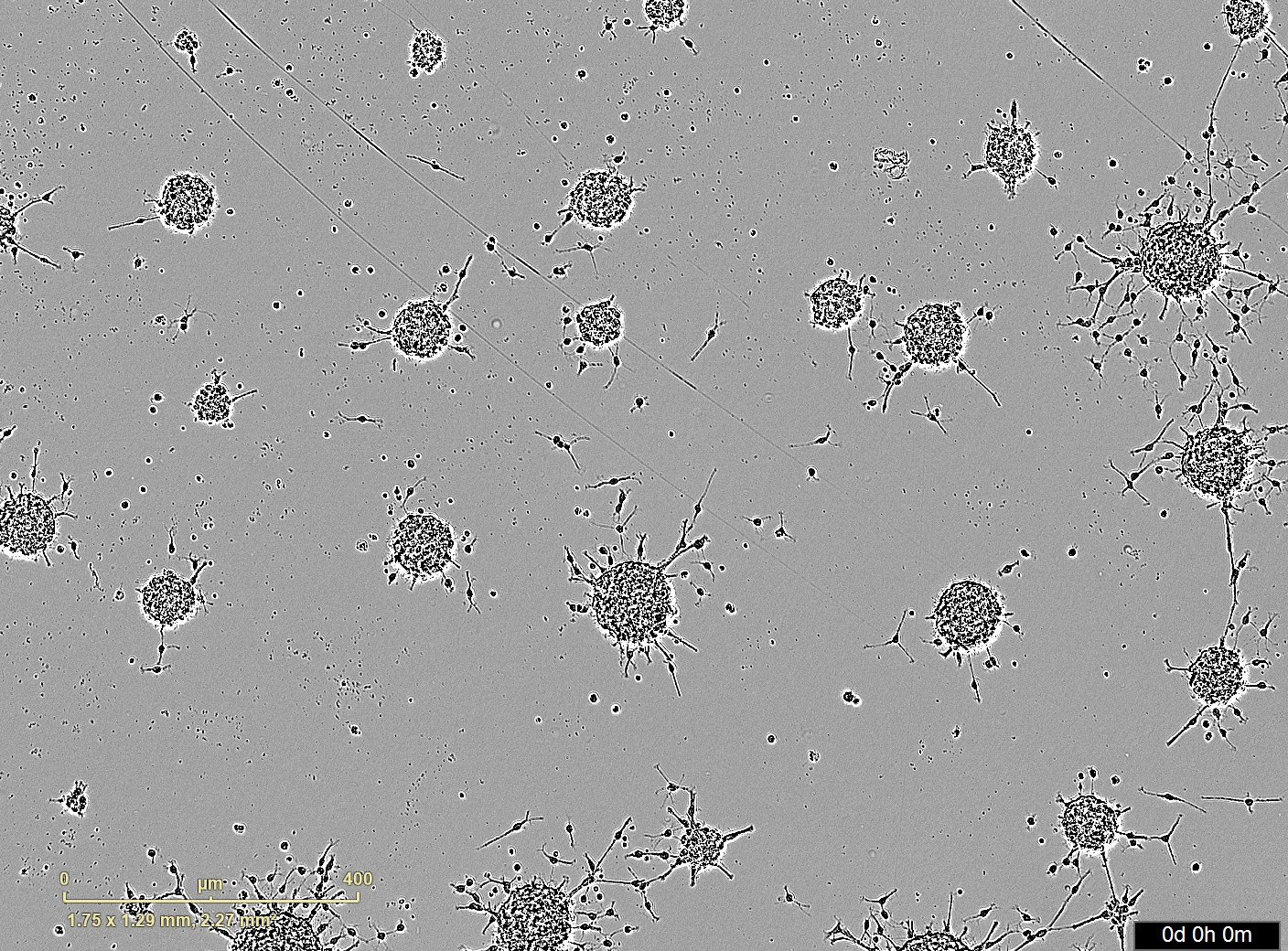

Supplement: Supplementary file 8 — Appendix Figure Source Data [file 44318_2026_752_MOESM8_ESM.zip › EMBOJ-2025-122043-Appendix source data/Appendix Figure S7/B/Larp7 ff.tif]

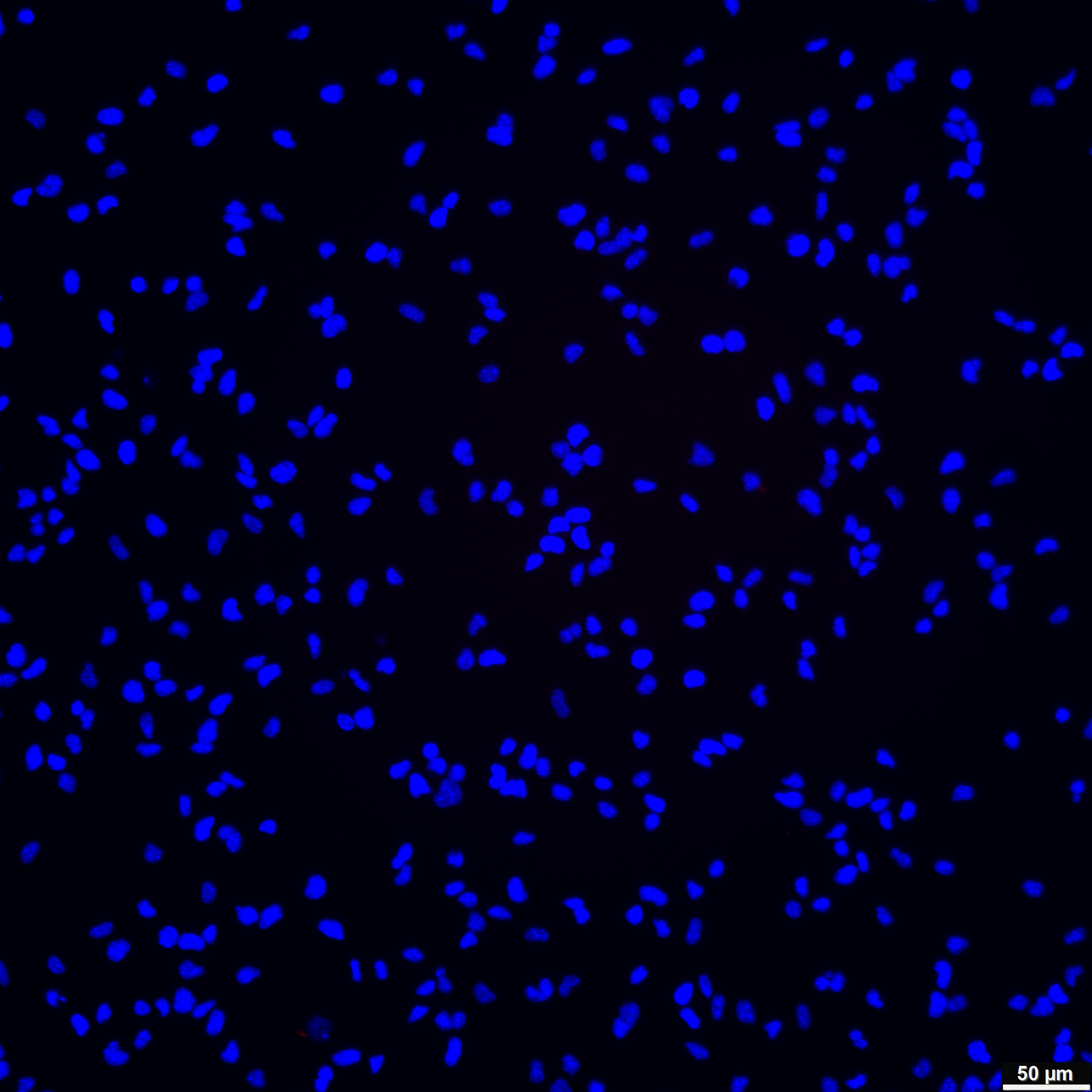

Supplement: Supplementary file 8 — Appendix Figure Source Data [file 44318_2026_752_MOESM8_ESM.zip › EMBOJ-2025-122043-Appendix source data/Appendix Figure S7/C/Larp7 ff;nestin-Cre Day 0.tif]

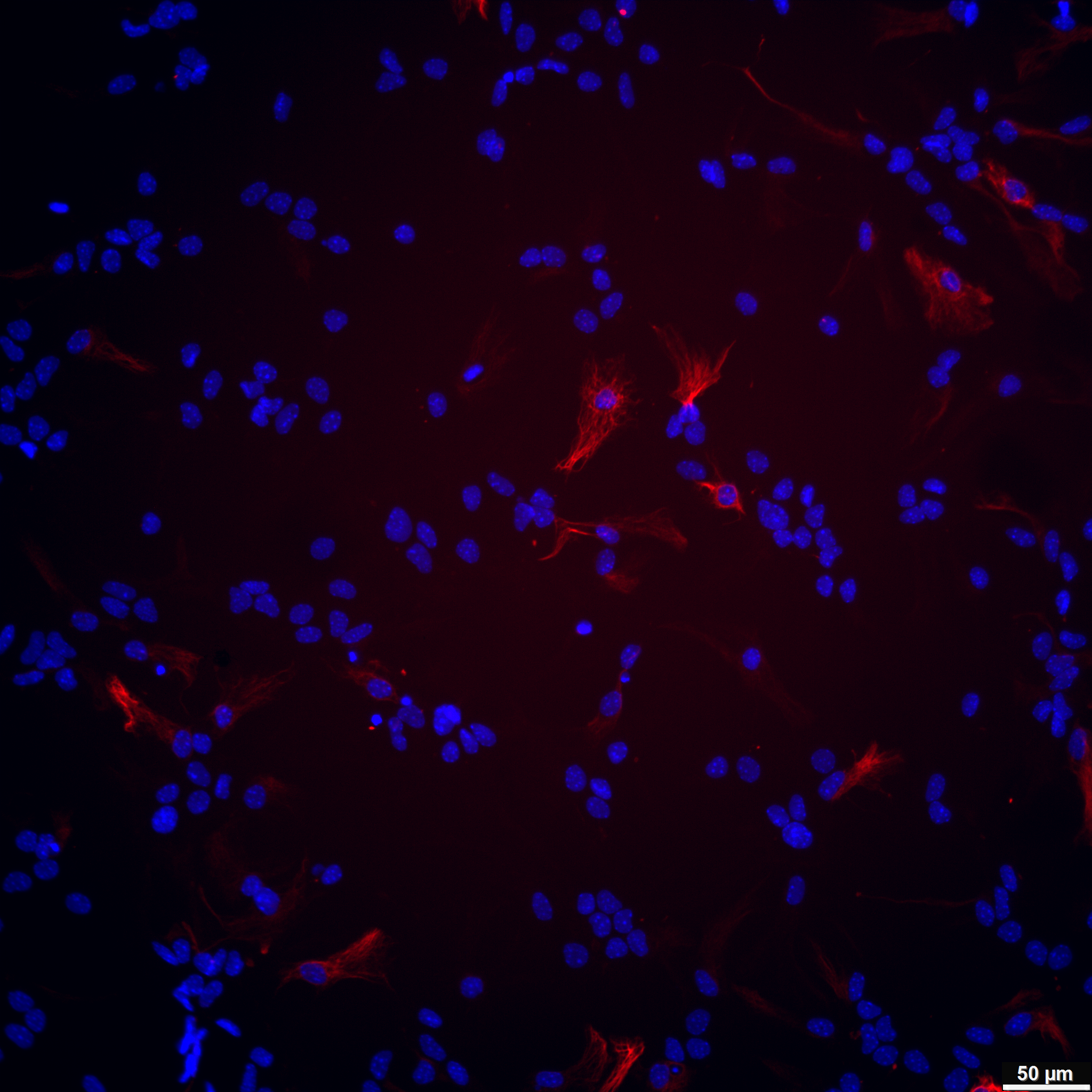

Supplement: Supplementary file 8 — Appendix Figure Source Data [file 44318_2026_752_MOESM8_ESM.zip › EMBOJ-2025-122043-Appendix source data/Appendix Figure S7/C/Larp7 ff;nestin-Cre Day 6 (DMSO).tif]

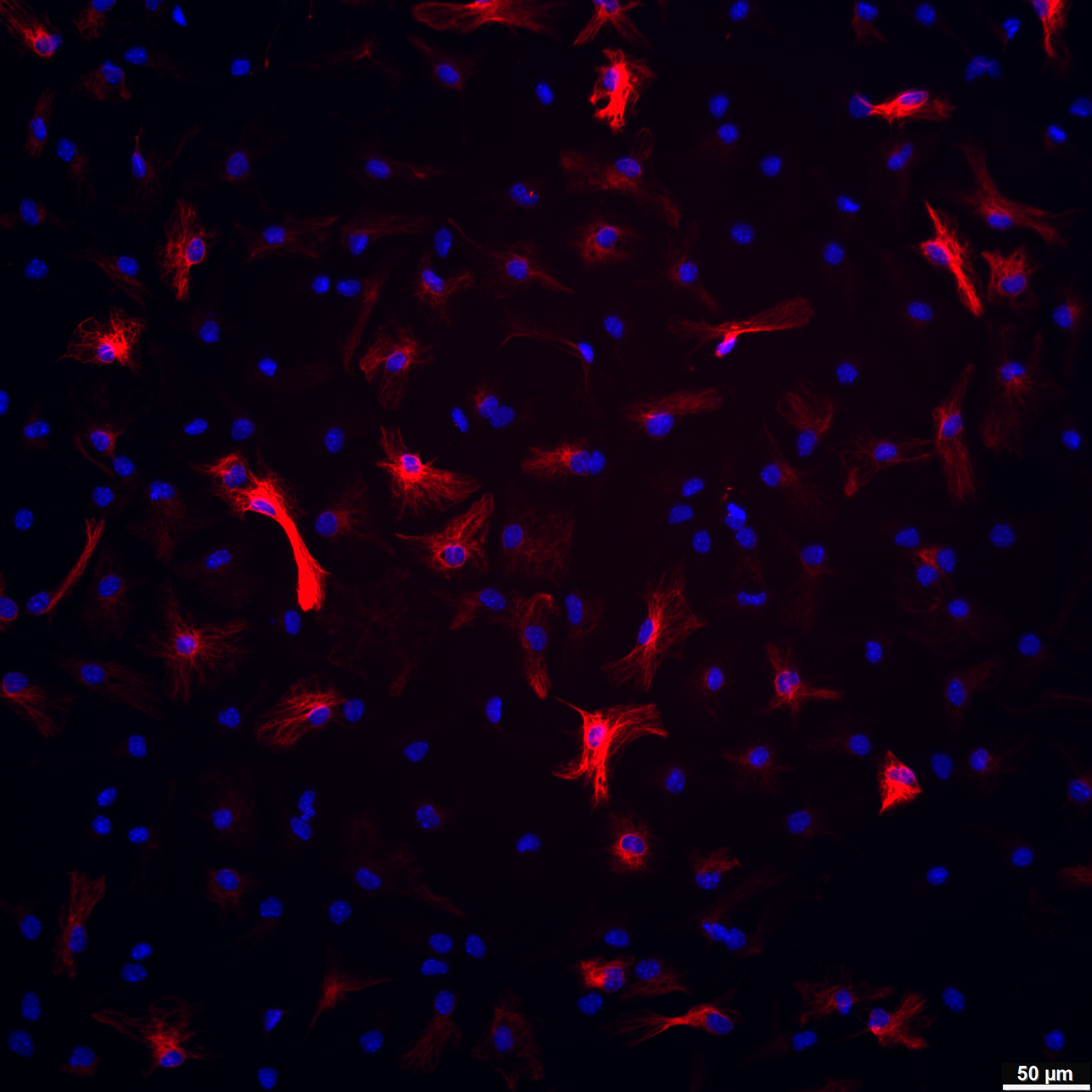

Supplement: Supplementary file 8 — Appendix Figure Source Data [file 44318_2026_752_MOESM8_ESM.zip › EMBOJ-2025-122043-Appendix source data/Appendix Figure S7/C/Larp7 ff;nestin-Cre Day 6 (Flavo).tif]

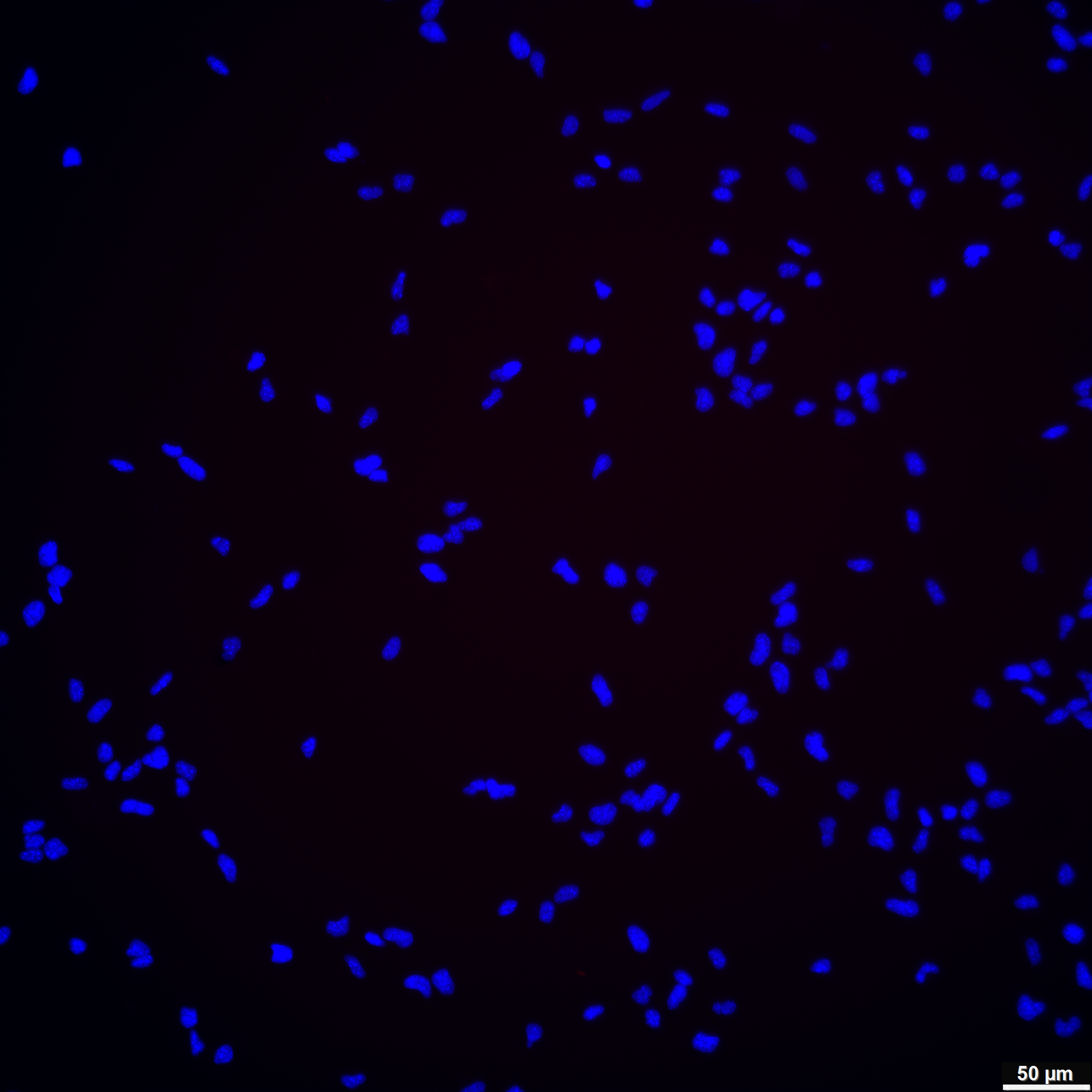

Supplement: Supplementary file 8 — Appendix Figure Source Data [file 44318_2026_752_MOESM8_ESM.zip › EMBOJ-2025-122043-Appendix source data/Appendix Figure S7/C/WT Day 0.tif]

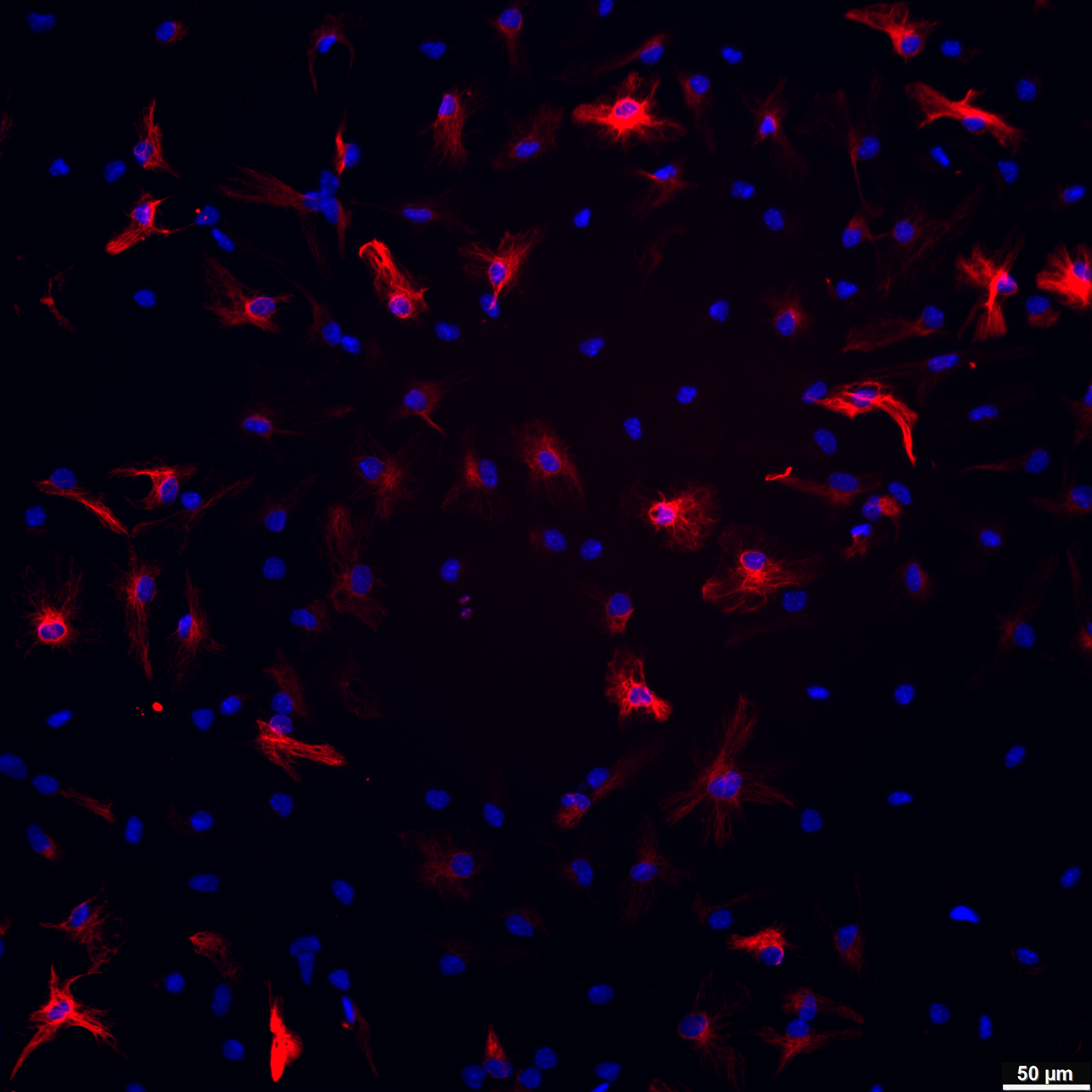

Supplement: Supplementary file 8 — Appendix Figure Source Data [file 44318_2026_752_MOESM8_ESM.zip › EMBOJ-2025-122043-Appendix source data/Appendix Figure S7/C/WT Day 6 (DMSO).tif]

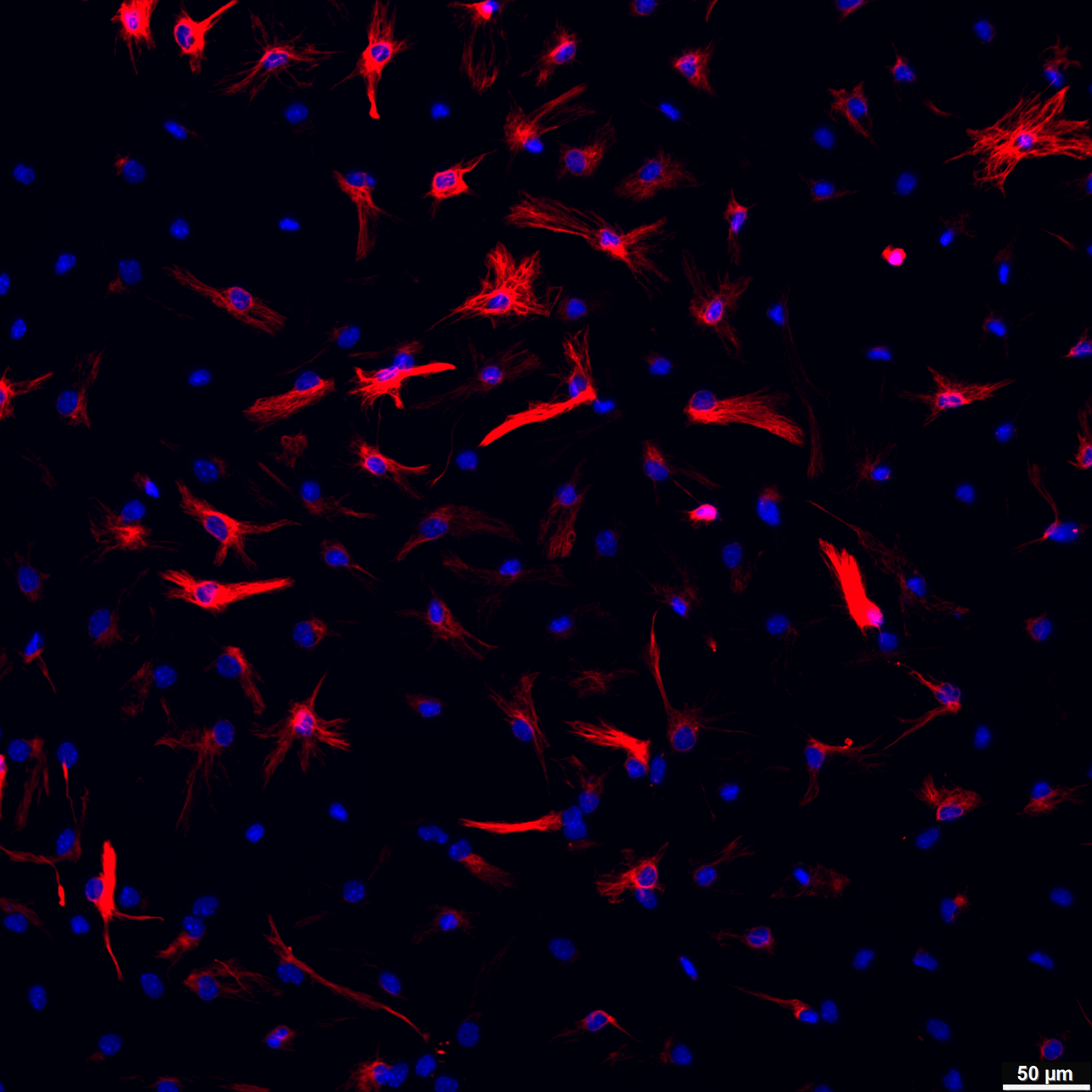

Supplement: Supplementary file 8 — Appendix Figure Source Data [file 44318_2026_752_MOESM8_ESM.zip › EMBOJ-2025-122043-Appendix source data/Appendix Figure S7/C/WT Day 6 (Flavo).tif]
